# G protein-coupled receptor kinase 5 (GRK5) contributes to impaired cardiac function and immune cell recruitment in post-ischemic heart failure

**DOI:** 10.1093/cvr/cvab044

**Published:** 2021-02-09

**Authors:** Claudio de Lucia, Laurel A Grisanti, Giulia Borghetti, Michela Piedepalumbo, Jessica Ibetti, Anna Maria Lucchese, Eric W Barr, Rajika Roy, Ama Dedo Okyere, Haley Christine Murphy, Erhe Gao, Giuseppe Rengo, Steven R Houser, Douglas G Tilley, Walter J Koch

**Affiliations:** 1 Center for Translational Medicine, Lewis Katz School of Medicine, Temple University, Philadelphia, PA, USA; 2 Department of Biomedical Sciences, College of Veterinary Medicine, University of Missouri, Columbia, MO, USA; 3 Cardiovascular Research Center, Lewis Katz School of Medicine, Temple University, Philadelphia, PA, USA; 4 Department of Medical, Surgical, Neurological, Metabolic and Aging Sciences, University of Campania “Luigi Vanvitelli”, Naples, Italy; 5 Department of Translational Medical Sciences, Division of Geriatrics, Federico II University, Via S. Pansini, 5, Naples, Italy; 6 Laboratory of neurovegetative system pathophysiology, Istituti Clinici Scientifici ICS Maugeri, IRCCS Istituto Scientifico di Telese Terme, Benevento, Italy

**Keywords:** Ischemic heart failure, Myocardial ischemia, Left ventricle, Immune system, Cardiac remodeling

## Abstract

**Aims:**

Myocardial infarction (MI) is the most common cause of heart failure (HF) worldwide. G protein-coupled receptor kinase 5 (GRK5) is upregulated in failing human myocardium and promotes maladaptive cardiac hypertrophy in animal models. However, the role of GRK5 in ischemic heart disease is still unknown. In this study, we evaluated whether myocardial GRK5 plays a critical role post-MI in mice and included the examination of specific cardiac immune and inflammatory responses.

**Methods and results:**

Cardiomyocyte-specific GRK5 overexpressing transgenic mice (TgGRK5) and non-transgenic littermate control (NLC) mice as well as cardiomyocyte-specific GRK5 knockout mice (GRK5cKO) and wild type (WT) were subjected to MI and, functional as well as structural changes together with outcomes were studied. TgGRK5 post-MI mice showed decreased cardiac function, augmented left ventricular dimension and decreased survival rate compared to NLC post-MI mice. Cardiac hypertrophy and fibrosis as well as fetal gene expression were increased post-MI in TgGRK5 compared to NLC mice. In TgGRK5 mice, GRK5 elevation produced immuno-regulators that contributed to the elevated and long-lasting leukocyte recruitment into the injured heart and ultimately to chronic cardiac inflammation. We found an increased presence of pro-inflammatory neutrophils and macrophages as well as neutrophils, macrophages and T-lymphocytes at 4-days and 8-weeks respectively post-MI in TgGRK5 hearts. Conversely, GRK5cKO mice were protected from ischemic injury and showed reduced early immune cell recruitment (predominantly monocytes) to the heart, improved contractility and reduced mortality compared to WT post-MI mice. Interestingly, cardiomyocyte-specific GRK2 transgenic mice did not share the same phenotype of TgGRK5 mice and did not have increased cardiac leukocyte migration and cytokine or chemokine production post-MI.

**Conclusions:**

Our study shows that myocyte GRK5 has a crucial and GRK-selective role on the regulation of leucocyte infiltration into the heart, cardiac function and survival in a murine model of post-ischemic HF, supporting GRK5 inhibition as a therapeutic target for HF.

## Graphical Abstract

**Figure:**
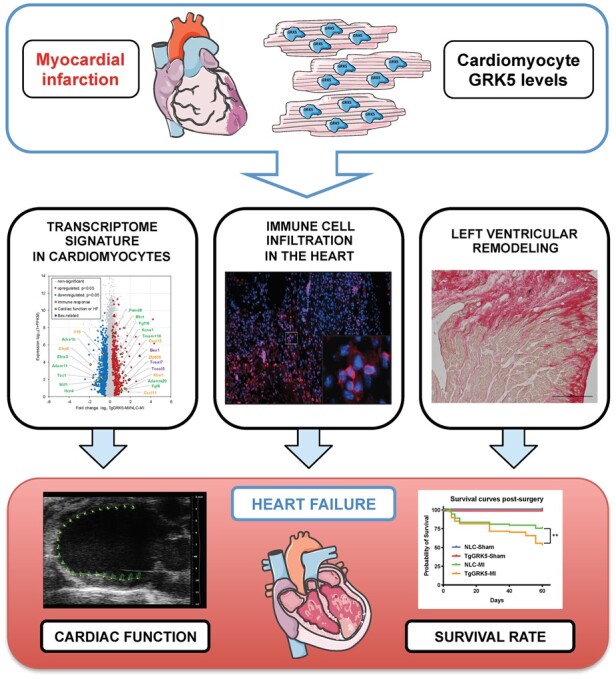

## 1. Introduction

Heart failure (HF) is the leading cause of morbidity, mortality and hospitalization worldwide, with myocardial infarction (MI) representing the most common cause.^1^ Even though survival rates have improved, HF still represents a major clinical, social, and economic burden. Hence, new therapeutic targets urgently need to be identified. Recent reports have shown that cardiac inflammation and immune cell recruitment are key players in left ventricular (LV) remodeling and can influence cardiac function/outcomes associated with the development of ischemic HF.^2^^,^^3^

It has also been described that G protein-coupled receptors (GPCRs) play a fundamental pathophysiological role in the heart.^4^ Both GPCR Kinase 2 (GRK2) and GRK5 are highly expressed in the heart and both are up-regulated in failing human myocardium and shown to be involved in HF pathogenesis in some animal models.^4–8^ While the canonical role of GRKs is to phosphorylate and induce receptor desensitization/recycling, it has been shown that GRK5 can also translocate to the nucleus of cardiomyocytes where it can exert GPCR-independent effects promoting maladaptive cardiac hypertrophy and dysfunction.^9^ Particularly, after pro-hypertrophic stimulation, GRK5 can translocate to the nucleus where it acts as a histone deacetylase 5 (HDAC5) kinase to enhance hypertrophic gene-transcription through MEF2 de-repression, is part of a DNA-binding complex and acts in a kinase-independent manner as a nuclear facilitator of the nuclear factor of activated T-cells (NFAT) activity promoting hypertrophic gene-transcription.^9–11^ Of note, we have previously shown transgenic mice with cardiomyocyte-specific GRK5 overexpression (TgGRK5) to have increased cardiac hypertrophy and functional impairments, while cardiomyocyte-specific GRK5 knockout mice (GRK5cKO) develop less hypertrophy and maintain better cardiac function after transverse aortic constriction (TAC).^9^^,^^12^ However, it is still unknown if GRK5 is involved in the establishment and development of ischemic heart disease. Recently, GRK5 has been also described as a regulator of Lipopolysaccharide (LPS)- and Toll-like receptor (TLR)-induced inflammatory cytokine/chemokine production and immune cell tissue infiltration *in vivo.*^9^^,^^13^ Thus, the aim of the present study was to assess whether myocardial GRK5 levels can affect immune cell recruitment to the heart following MI and ultimately influence cardiac function and survival in a murine model of post-ischemic HF.

## 2. Methods

### 2.1 Animal models and experimental procedures

All animal procedures were approved by the Institutional Animal Care and Use Committee of Temple University and were performed in accordance with the NIH Guide for the Care and Use of Laboratory Animals. We used previously generated TgGRK5, TgGRK2 and appropriate NLC (non-transgenic littermate control) mice as well as GRK5cKO and relative WT mice.^12^^,^^14^^,^^15^

MI was induced as previously described.^16^ Mice were anesthetized with 3% isoflurane inhalation (both induction and maintenance of anaesthesia). A small skin incision was made, and the pectoral muscles were retracted to expose the fourth intercostal space. A small hole was made, and the heart was popped out. Left main descending coronary artery (LCA) was sutured ≈3 mm from its origin to assure a permanent occlusion, and the heart was placed back into the intrathoracic space, followed by the closure of muscle and skin. The sham group underwent the same surgical procedure except that the LCA was not occluded. Animals were randomly assigned to MI or sham operation and, received a single dose (0.1 mg/kg) of buprenorphine immediately after surgery.

Echocardiography and hemodynamics were performed as described.^17^^,^^18^ For the echocardiography, mice were anesthetized with isoflurane inhalation (induction 3% and maintenance 1.5–2%). For the hemodynamic analysis *in vivo*, mice were anesthetized with Tribromoethanol (Avertin, intraperitoneal dose of 2.5%, 20 μl/g body weight; used only for terminal hemodynamics). Avertin was chosen for anesthesia over other compounds because it has been shown to have less side effects on cardiovascular and hemodynamic stability and, therefore interferes less with the analysis of heart function and inotropic reserve during in vivo hemodynamic analysis.^19^^,^^20^ A recent review of the literature indicates that Avertin exerts less depressant effects on LV function and heart rate (HR) compared to isoflurane.^20^ Moreover, compared with Avertin, a combination of xylazine and ketamine was demonstrated to produce profound bradycardia with effects on loading conditions and ventricular function.^19^ At LV catheterization, systolic and diastolic function was particularly impaired in mice that showed HR <300 beats/min after xylazine and ketamine anesthesia.^19^

Mice were euthanized with the following methods: carbon dioxide 100% (route: inhalation), followed by cervical dislocation; exsanguination under isoflurane 3% anesthesia (route: inhalation); isoflurane 3% (route: inhalation) followed by heart excision.

### 2.2 Histology and isolation of adult cardiac myocytes

Cardiac apoptosis, fibrosis, infarct size and infarct length evaluation, immunohistochemistry and isolation of adult cardiomyocytes were performed as previously described.^18^^,^^21^

### 2.3 RT-PCR, Western blotting, and RNA-seq

Real-time polymerase chain reaction (RT-PCR), Western blotting, and RNA-seq were performed as previously described^10^^,^^21^^,^^22^

### 2.4 FACS analysis

Fluorescence activated cell sorting (FACS) analysis of immune cell populations was performed on cells isolated from heart, as previously described^23^^,^^24^

### 2.5 Statistical analysis

Mean values for individual experiments are expressed as means ± standard error of the mean (S.E.M.). Shapiro–Wilk test was used to test normality. Comparisons between two groups were assessed by unpaired *t*-test if normally distributed or by Mann–Whitney test if not normally distributed. Comparisons between multiple groups were performed by one-way or two-way ANOVA with Tukey’s multiple comparison tests. Log-rank (Mantel–Cox) test has been used to compare survival curves. Prism 8.4 software (GraphPad Software) has been used for statistical analysis. A *P*-value < 0.05 was considered statistically significant.

## 3. Results

### 3.1 TgGRK5 mice showed reduced cardiac function and increased hypertrophy after MI

**Figure 1:**
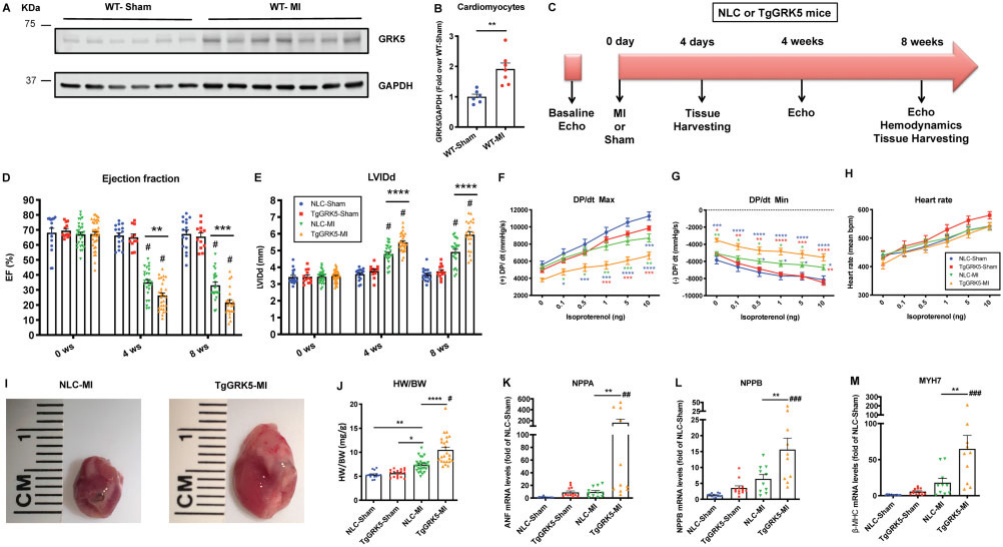
Cardiomyocyte-specific GRK5 overexpression impairs cardiac function after myocardial infarction. Representative Western blot (*A*) and quantification (*B*) showing GRK5 protein levels in cardiomyocytes isolated from WT mice at 4-days post-Sham operation or post–myocardial infarction (MI). GAPDH used as loading control (*n* = 6–7). Overall design of the 8-week study in NLC and TgGRK5 mice subjected to Sham-operation or MI (*C*). Ejection fracti) (*D*) on (EFand left ventricular internal diameter in diastole (LVIDd) (*E*) as measured by echocardiography at 0- (baseline), 4- and 8-weeks post-surgery in Sham and MI groups (*n* = 11–38). Quantification of LV +dP/dt Max (*F*), −dP/dt Min (*G*) and heart rate (HR) (*H*) performed at baseline and with increasing doses of Isoproterenol. Different color asterisks have been used to show significant differences between groups (*n* = 7–18). Representative images of hearts from MI groups (8 weeks post-MI) (*I*). Measures of heart weight to body weight ratio (HW/BW) at 8 weeks post-surgery (*J*) (*n* = 15–27). Quantification of RT-PCR data showing fold change of NLC-Sham for NPPA (*K*), NPPB (*L*) and MYH7 (*M*) in LV samples at 8-weeks post-surgery (*n* = 10–14). *P < 0.05, **P < 0.01, ***P < 0.001, ****P < 0.0001, #*P* < 0.0001 vs. NLC-Sham and TgGRK5-Sham, ##*P* < 0.01 vs. NLC-Sham and TgGRK5-Sham, ###*P* < 0.0001 vs. NLC-Sham and *P* < 0.001 vs. TgGRK5-Sham. Two- or one-way ANOVA with Tukey’s post hoc test or, unpaired *t*-test were used between groups.

Previous studies have reported upregulated myocardial GRK5 expression in human HF biopsies.^5–7^ Hence, we evaluated LV GRK5 protein levels 4-days post-MI or sham operation in WT mice. We found LV GRK5 protein levels to be increased in WT-MI compared to WT-Sham mice (Supplementary material online, *Figure S1A, B*). More interestingly, we discovered that GRK5 was upregulated in cardiomyocytes isolated from WT-MI compared to WT-Sham mice at 4 days post-surgery (*Figure 1A,B*). Importantly, after subcellular fractionation of cardiomyocytes isolated from WT mice at 3 days post-MI or -Sham, we found that GRK5 levels were equally increased in the cytosolic, membrane and nuclear fractions post-MI (Supplementary material online, *Figure S1C, D*).

In order to investigate the impact of myocardial GRK5 on cardiac function in ischemic HF *in vivo*, we utilized TgGRK5 mice compared to NLC mice. To study ischemic heart disease, 8 to 12-week-old TgGRK5 and NLC mice of both male and female sexes, underwent surgical MI or Sham operation (Study design in *Figure 1C*). Serial measurements of cardiac function were collected for all mice at 0 (baseline), 4-, and 8-weeks post-surgery using echocardiography (*Figure 1D,E*; Supplementary material online, *Figure S2A–D* and *Table S1*). No differences in LV function or dimension were found between groups at baseline (*Figure 1D,E* and Supplementary material online, *Table S1*). At both 4- and 8-weeks post-MI, LV ejection fraction (EF) and fractional shortening (FS) were significantly reduced in post-MI groups compared to Sham animals, while LV internal diameters in diastole and systole (LVIDd and LVIDs) and LV volumes were significantly higher in post-MI compared to Sham mice, which was expected (*Figure 1D,E* and Supplementary material online, *Figure S2A–D* and *Table S1*). Interestingly, our data show significant detrimental effects of cardiomyocyte-specific overexpression of GRK5 in ischemic HF (4- and 8-weeks post-MI) both in terms of contractility and LV dimension (*Figure 1D,E* and Supplementary material online, *Figure S2C, D* and *Table S1*). LV function and diameter were impaired in both male and female TgGRK5 mice when compared to sex-matched NLC mice during the progression of post-MI HF (Supplementary material online, *Figure S3A–D*). In line with diminished LV function, radial and longitudinal strain/strain rate were also impaired in TgGRK5 post-MI mice (Supplementary material online, *Figure S4A–F*). In fact, TgGRK5 post-MI mice showed impaired global (average of 6 LV segments) and anterior-apical longitudinal strain compared to NLC-MI mice while the main difference in radial contractility between these groups lies in the anterior-basal segment (Supplementary material online, *Figure S4C, D*).

**Figure 2:**
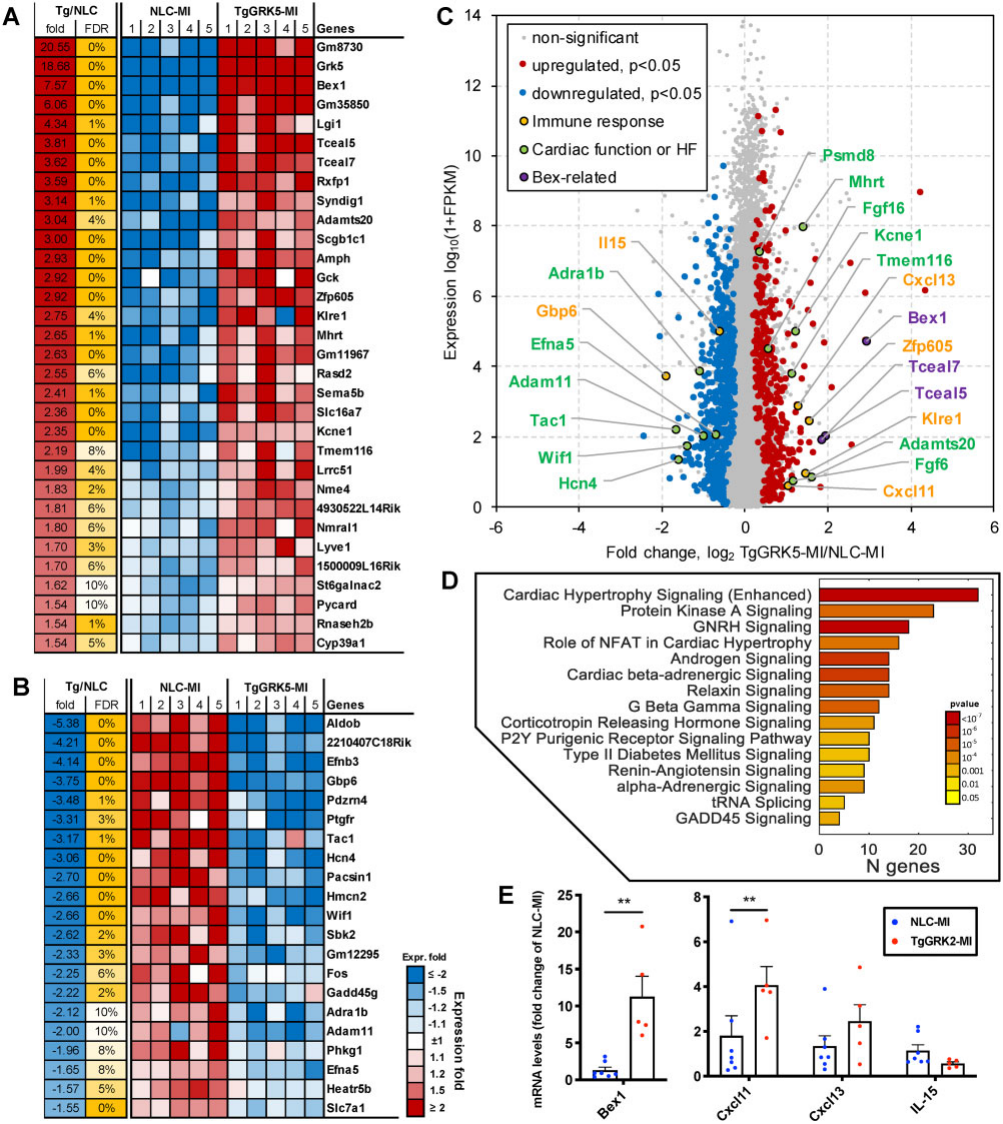
Effect of GRK5 expression on transcriptome in cardiomyocyte after myocardial infarction. Transcriptome evaluation in cardiomyocytes isolated at 4-days post-myocardial infarction (MI) from TgGRK5 mice compared to NLC mice (5 mice/group). Heat map illustrating top genes upregulated (*A*) and top genes downregulated (*B*) in TgGRK5-MI (compared to NCL-MI) that pass FDR < 10% threshold. Volcano plot showing upregulated and downregulated genes in TgGRK5-MI compared to NLC-MI (*C*). Highlighted significantly affected genes. Pathways enriched among genes significantly affected by GRK5-overexpression in cardiomyocytes (*D*). Quantification of RT-PCR data showing fold change of NLC-MI for Bex1, Cxcl11, Cxcl13 and IL-15 in cardiomyocytes isolated at 4 days post-MI (*E*) (*n* = 5–7). *P < 0.05, **P < 0.01. Mann–Whitney test was used among groups.

**Figure 3:**
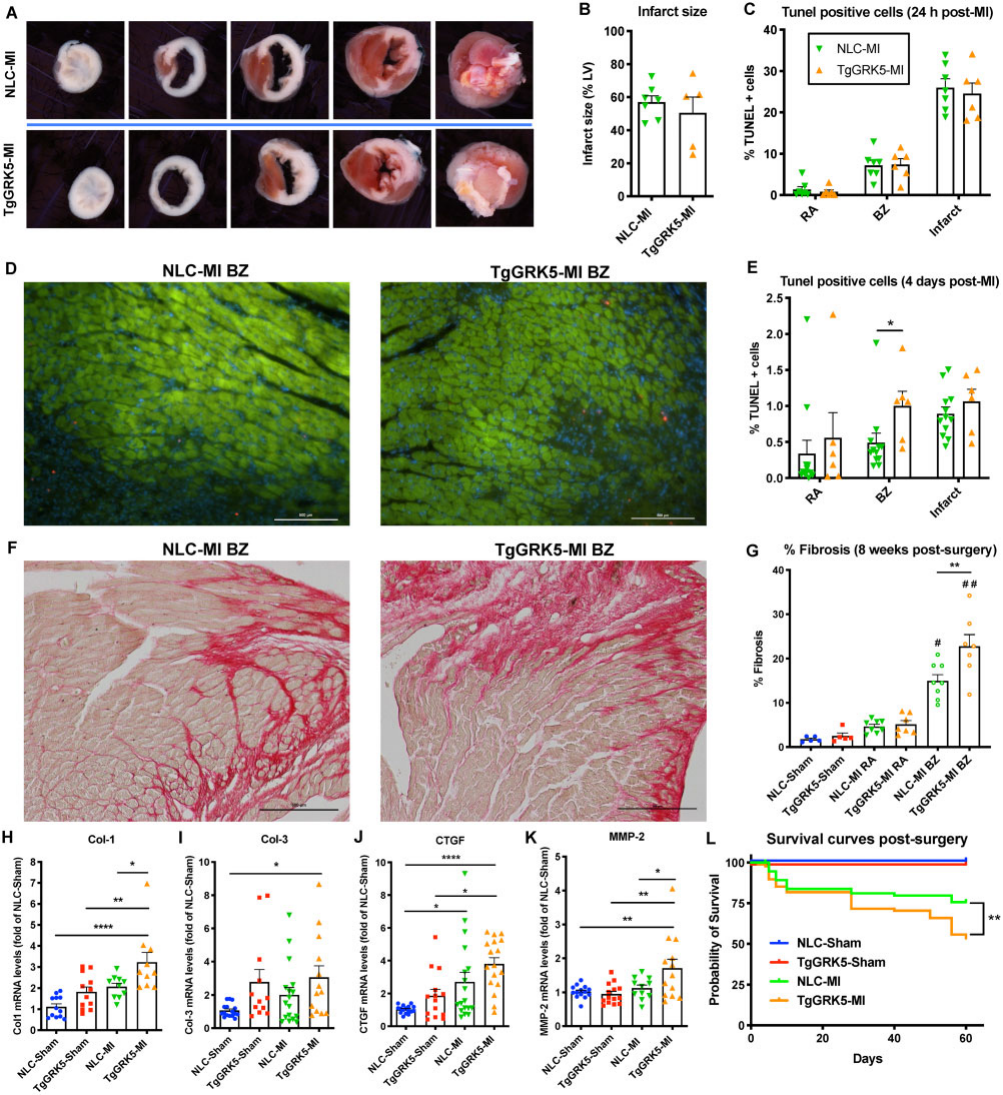
Transgenic cardiomyocyte GRK5 overexpression impacts cardiac remodeling and survival in ischemic heart failure. Representative TTC staining of hearts at 24 h post-myocardial infarction (MI) of NLC and TgGRK5 groups (*A*) as well as quantification of infarct size (% of LV) (*B*) (*n* = 5–7). TUNEL quantification of images of left ventricular (LV) tissue from NLC and TgGRK5 mice at remote area (RA), border zone (BZ), and infarct area at 24 h post-MI (*C*) (*n* = 6–7). Representative TUNEL staining (red) of LV tissue from NLC and TgGRK5 mice at the BZ counterstained with Troponin T (green) to visualize cardiomyocytes, at 4-days post-MI; DAPI was used as a nuclear marker (blue) (*D*). Scale bar in white (500 μm). TUNEL quantification of images of LV tissue from NLC and TgGRK5 mice at the RA, BZ, and infarct area, at 4-days post-MI (*E*) (*n* = 6–12). Representative images (*F*) and quantitative data (*G*) showing the percentage of fibrosis via Picrosirius red staining in LV tissue from NLC and TgGRK5 at 8-weeks post-Sham operation or MI (*n* = 5–8). Scale bar in black (500 μm). Quantification of RT-PCR data showing fold change of NLC-Sham for Col-1 (*H*), Col-3 (*I*), CTGF (*J*) and MMP-2 (*K*) in LV samples collected at 8-weeks post-surgery (*n* = 10–18). Kaplan–Meier survival curves of Sham and MI groups (*L*) (*n* = 8–89). Log-rank (Mantel–Cox) test has been used between groups. *P < 0.05, **P < 0.01, ****P < 0.0001, #*P* < 0.0001 vs. NLC-Sham, TgGRK5-Sham and NLC-MI RA and *P* < 0.001 vs. TgGRK5-MI RA, ##*P* < 0.0001 vs. NLC-Sham, TgGRK5-Sham, NLC-MI RA and TgGRK5-MI RA. One-way ANOVA with Tukey’s post hoc test, *t*-test or Mann–Whitney test were used between groups.

**Figure 4:**
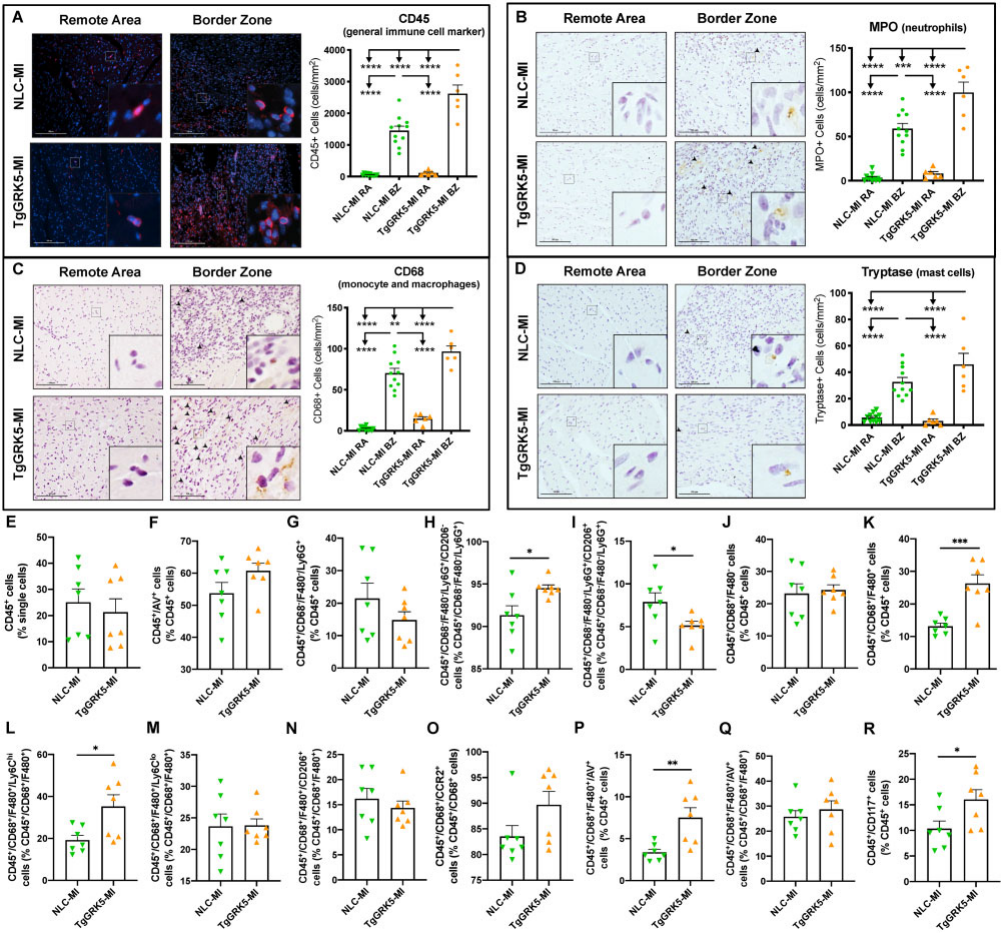
TgGRK5 showed increased early immune cell recruitment to the heart post-myocardial infarction. Representative images and quantification of CD45 (*A*), MPO (*B*), CD68 (*C*) and tryptase (*D*) stainings for remote area (RA) and border zone (BZ) of hearts from NLC and TgGRK5 mice at 4-days post-myocardial infarction (MI) (*n* = 6–11). Scale bar in white or black (100 μm). Arrowheads indicate positive staining. Insets show higher magnification at ×250. Flow-cytometric quantification of immune cells isolated from NLC and TgGRK5 hearts at 4 days post-MI (*E*–*R*) (*n* = 7 per group). AV= Annexin V. *P < 0.05, **P < 0.01, ***P < 0.001, ****P < 0.0001. One-way ANOVA with Tukey’s post hoc test, *t*-test or Mann–Whitney test were used between groups.

At the end of the study, LV catheterization and hemodynamic analysis showed a significant reduction in LV contractility and relaxation while no difference in HR at both baseline and after increasing doses of Isoproterenol in the post-MI compared to Sham groups (*Figure 1F–H*). Interestingly, TgGRK5 mice subjected to ischemic injury showed a significant impairment in LV +dP/dt and −dP/dt compared to NLC post-MI mice (no difference in HR) at baseline and after infusion of Isoproterenol (*Figure 1F–H*).

To assess cardiac hypertrophy, we evaluated heart weight (HW), HW to body weight ratio (HW/BW), and HW to tibia length ratio (HW/TL) at the end of the study in all groups. HW, HW/BW, and HW/TL were increased in NLC ischemic hearts compared to Shams and significantly further increased in TgGRK5 post-MI hearts (representative pictures of post-MI groups at the end of the study have been showed) regardless of sex (*Figure 1I,J* and Supplementary material online, *Figures S5A,B* and *S6A–F*).

**Figure 5:**
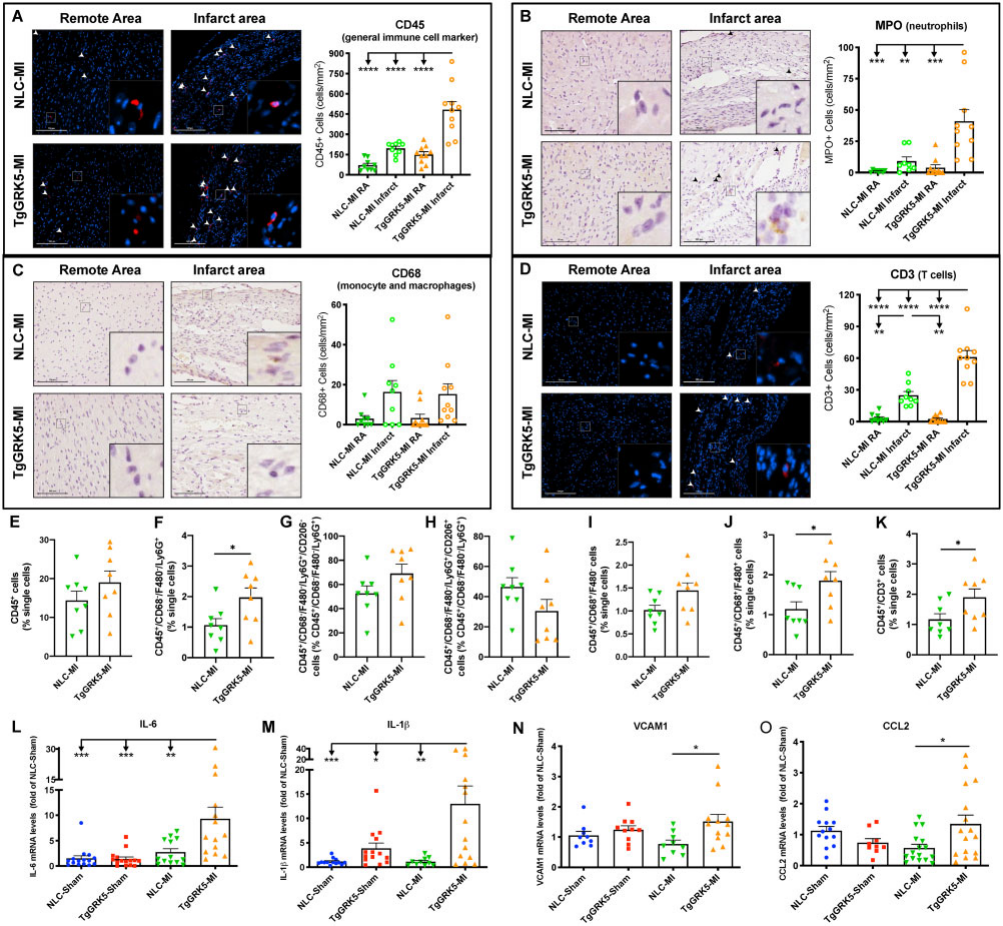
TgGRK5 mice showed increased late immune cell infiltration in the heart and chronic cardiac inflammation post-myocardial infarction. Representative images and quantification of CD45 (*A*), MPO (*B*), CD68 (*C*) and CD3 (*D*) stainings for remote area (RA) and infarct area of hearts from NLC and TgGRK5 mice at 8 weeks post-myocardial infarction (MI) (*n* = 8–10). Scale bar in white or black (100 μm). Arrowheads indicate positive staining. Insets show higher magnification at ×250. Flow-cytometric quantification of immune cells isolated from NLC and TgGRK5 hearts at 4 weeks post-MI (*E*–*K*) (*n* = 8 per group). Quantification of RT-PCR data showing fold change of NLC-Sham for IL-6, IL-1β, VCAM1, CCL2, (*L*–*O*) in left ventricular samples collected at 8-weeks post-surgery (*n* = 8–16). *P < 0.05, **P < 0.01, ***P < 0.001, ****P < 0.0001. One-way ANOVA with Tukey’s post hoc test or *t*-test were used between groups.

**Figure 6:**
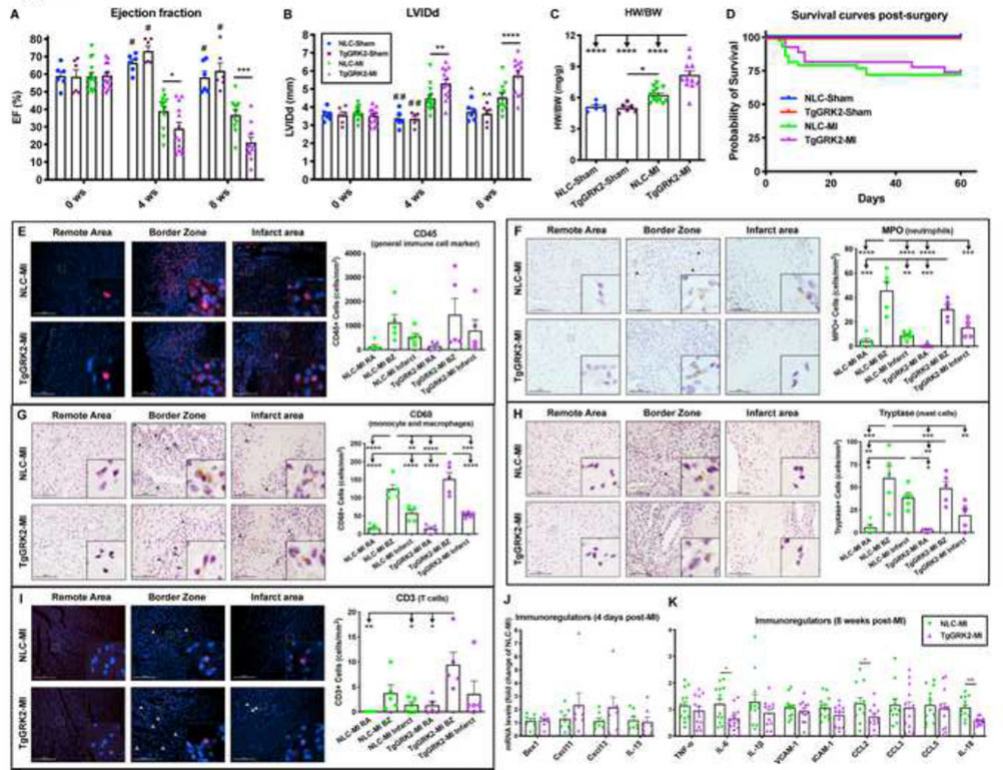
Cardiomyocyte-specific GRK2 overexpression did not alter immune cell recruitment to the heart post-myocardial infarction. Ejection fraction (EF) (*A*) and left ventricular internal diameter in diastole (LVIDd) (*B*) as measured by echocardiography at 0 (baseline), 4- and 8-weeks post-Sham operation or myocardial infarction (MI) in NLC and TgGRK2 groups (*n* = 6–14). Measures of heart weight to body weight ratio (HW/BW) at 8 weeks post-surgery (*C*) (*n* = 6–13). Kaplan–Meier survival curves of Sham and MI groups (*D*) (*n* = 8–43). Log-rank (Mantel–Cox) test has been used between groups. Representative images and quantification of CD45 (*E*), MPO (*F*), CD68 (*G*), tryptase (*H*) and CD3 (*I*) stainings for remote area (RA), border zone (BZ), and infarct area of hearts from NLC and TgGRK2 mice at 4-days post-MI. Scale bar in white or black (100 μm). Arrowheads indicate positive staining. Insets show higher magnification at ×250 (*n* = 5 per group). Quantification of RT-PCR data showing fold change of NLC-MI for Bex1, Cxcl11, Cxcl13 and IL-15 (*J*) in LV samples collected at 4 days post-MI (*n* = 7 per group). Quantification of RT-PCR data showing fold change of NLC-MI for TNFα, IL-6, IL-1β, VCAM1, ICAM1, CCL2, CCL3, CCL5, and IL-18 (*K*) in LV samples collected at 8-weeks post-MI (*n* = 11–13). *P < 0.05, **P < 0.01, ***P < 0.001, ****P < 0.0001, #*P* < 0.0001 vs. NLC-MI and TgGRK2-MI, ##*P* < 0.01 vs. NLC-MI and *P* < 0.0001 vs. TgGRK2-MI, ^*P* < 0.0001 vs. TgGRK2-MI, ^^*P* < 0.01 vs. NLC-MI *P* < 0.0001 vs. TgGRK2-MI. Two- or one-way ANOVA with Tukey’s post hoc test, t-test or Mann–Whitney test were used between groups.

Consistent with functional and gravimetric data, atrial natriuretic factor (NPPA), brain natriuretic peptide (NPPB), and β-Myosin heavy chain (MYH7) gene expression was markedly increased in TgGRK5 failing hearts compared with NLC failing hearts at 8-weeks post-MI (*Figure 1K–M*). As expected, GRK2 protein levels were increased 4-days post-MI in the NLC hearts compared to NLC sham hearts (Supplementary material online, *Figure S7A, B*).^4^ Interestingly, we found that GRK2 protein levels were further upregulated in TgGRK5-MI compared to NLC-MI hearts (Supplementary material online, *Figure S7A, B*).

**Figure 7:**
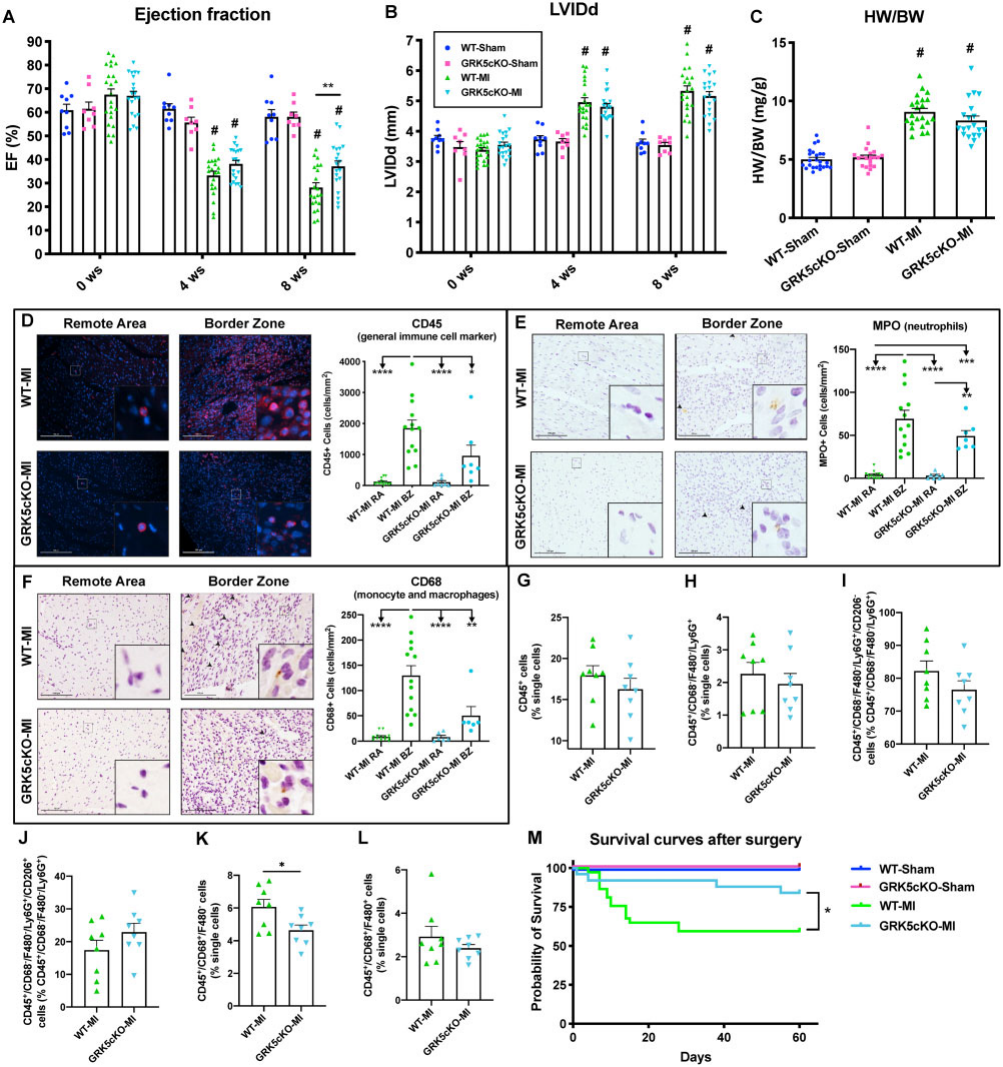
Cardiomyocyte-GRK5 deletion improved contractility, diminished early immune cell recruitment to the heart and increased survival rate post-myocardial infarction. Ejection fraction (EF) (*A*) and left ventricular internal diameter in diastole (LVIDd) (*B*) as measured by echocardiography at 0 (baseline), 4- and 8-weeks post-Sham operation or myocardial infarction (MI) in WT and GRK5cKO groups (*n* = 8–23). Measures of heart weight to body weight ratio (HW/BW) at 8 weeks post-surgery (*C*) (*n* = 20–24). Representative images and quantification of CD45 (*D*), MPO (*E*) and CD68 (*F*) stainings for remote area (RA) and border zone (BZ) of hearts from WT and GRK5cKO mice at 4-days post-MI. Scale bar in white or black (100 μm). Arrowheads indicate positive staining. Insets show higher magnification at ×250 (*n* = 6–13). Flow-cytometric quantification of immune cells isolated from WT and GRK5cKO hearts at 4 days post-MI (*G*–*L*) (*n* = 8 per group). Kaplan–Meier survival curves of Sham and MI groups (*M*) (*n* = 11–37). Log-rank (Mantel–Cox) test has been used between groups. *P < 0.05, **P < 0.01, ***P < 0.001, ****P < 0.0001; #*P* < 0.0001 vs. WT-Sham and GRK5cKO-Sham. Two- or one-way ANOVA with Tukey’s post hoc test, or *t*-test were used between groups.

### 3.2 Transcriptome analysis in TgGRK5 cardiomyocytes subjected to ischemic injury

It is well established that ischemic injury activates various signaling pathways, however, it is still unknown whether overexpression of GRK5 in cardiomyocytes leads to changes in gene expression patterns post-MI.^25^ We used RNA-Seq technology to assess whole transcriptome expression in cardiomyocytes isolated 4-days post-MI from TgGRK5 mice compared to NLC. We found 32 top upregulated genes (that passed FDR < 10% threshold) (*Figure 2A*) and 21 top downregulated genes (*Figure 2B*) in TgGRK5 cardiomyocytes (complete list of upregulated and downregulated genes in TgGRK5 compared to NLC post-MI cardiomyocytes in Supplementary material online, *Tables S2* and *S3*). These genes are predominantly known as regulators of the immune system as well as of cardiac function, remodeling or HF-related (*Figure 2C*). Enrichment analysis of the significantly differentially expressed genes using Ingenuity Pathway Analysis (IPA) demonstrated several pathways and interaction networks (related to cardiovascular function and immune-inflammatory response) being affected post-MI by GRK5 overexpression in cardiomyocytes (*Figure 2D* and Supplementary material online, *Figure S8*).

Cardiac pro-hypertrophic pathways including NFAT-signaling as well as Gβγ, Protein Kinase A and β-AR and α-AR signaling were triggered. Moreover, numerous hormonal pathways were also activated such as Renin–Angiotensin, GNRH, androgen, and CRH signaling. Consistent with NFAT-activation, expression levels of the NFAT-target gene RCAN was increased in post-MI LV tissue from TgGRK5 compared to NLC (Supplementary material online, *Figure S9A*).

We confirmed altered expression of immunomodulatory factors (Bex1, Cxcl11, Cxcl13, and IL-15) in cardiomyocytes isolated 4-days post-MI from TgGRK5 mice compared to NCL-MI via RT-PCR (*Figure 2E*). Interestingly, Bex1 and Cxcl11 expression was also increased in LV samples from TgGRK5-Sham compared to NLC-Sham mice (Supplementary material online, *Figure S9B*). Of note, 2 other genes from the Bex family were upregulated in TgGRK5 cardiomyocytes as shown by our transcriptome analysis: Tceal7 (also called Bex4; a microtubule-associated protein that regulates tubulin dynamics) and Tceal5 (gene containing Bex domain) (*Figure 2C*; Supplementary material online, *Table S2*). Moreover, gene expression of HDAC5 and HDAC6 was reduced in post-MI cardiomyocytes when GRK5 was upregulated (Supplementary material online, *Table S3*).

### 3.3 Cardiomyocyte overexpression of GRK5 impacts cardiac remodeling and survival in ischemic HF

Infarct size, measured by TTC staining at 24 h post-MI, revealed that TgGRK5 mice had infarcts of comparable size to NLC mice, suggesting that cardiomyocyte-specific overexpression of GRK5 does not alter the response to cardiac injury and served as verification that the surgical technique used was consistent across groups (*Figure 3A,B*). In addition, TUNEL staining showed that TgGRK5 hearts had comparable apoptosis to NLC hearts post-ischemic injury (24 h post-MI) in the remote area (RA), border zone (BZ), and infarct area (*Figure 3C* and Supplementary material online, *Figure S10A–F*). However, TUNEL staining demonstrated that TgGRK5 hearts had significantly higher apoptotic cells in the BZ compared to NLC hearts at 4-days post-MI but these cells were not found to be cardiomyocytes (*Figure 3D,E* and Supplementary material online, *Figure S10K*). No differences were found between TgGRK5 and NLC post-MI hearts in terms of apoptosis in the RA and infarct area at 4-days post-MI (*Figure 3E* and Supplementary material online, *Figure S10G–J*). Moreover, cardiac fibrosis in the BZ (*Figure 3F,G*), as well as mRNA levels of Collagen-1 (Col-1), CTGF, and MMP2, were higher in TgGRK5 compared to NLC mice in a chronic phase of HF (8-weeks post-MI) (*Figure 3H,J,K*). No differences in the development of fibrosis were found between sham or failing hearts in the RA (*Figure 3G* and Supplementary material online, *Figure S10L–O*); Col-3 expression levels were also similar between NLC and TgGRK5 failing hearts (*Figure 3I*).

Moreover, TgGRK5 hearts showed a reduction in vessels/cardiomyocyte ratio in the RA compared to NLC hearts while no difference was found between these groups in terms of infarct length at 8 weeks post-MI (Supplementary material online, *Figures S11* and *S12*). Importantly, survival rate was decreased in TgGRK5 post-MI mice compared to NLC post-MI mice (*Figure 3L*). Myocardial overexpression of GRK5 was affecting post-MI mortality in both sexes (Supplementary material online, *Figure S13A, B*).

### 3.4 Cardiomyocyte GRK5 overexpression increased leukocyte recruitment to the heart and chronic cardiac inflammation following MI

Based on our transcriptome data showing the impact of GRK5 on the production of immunomodulatory factors by cardiomyocytes, we sought to determine whether cardiomyocyte-specific GRK5 upregulation *in vivo* could impair the leukocyte infiltration phenotype at either early or late time-points post-MI. To assess this, immunohistochemistry was performed on heart sections at 4-days and 8-weeks post-surgery to quantify infiltration of immune cell populations in sham hearts and in specific areas of MI hearts (RA, BZ, and infarct areas) in both TgGRK5 and NLC groups (*Figures 4A–D* and *5A–D*; Supplementary material online, *Figures S14 and S20*). Furthermore, FACS analysis (gating strategy shown in Supplementary material online, *Figure S15*) was performed to quantify immune cells (and their sub-populations) in TgGRK5 and NLC hearts at 4 days and 4 weeks post-MI (*Figures 4E–R* and *5E–K*; Supplementary material online, *Figures S16–18, S21–S23*). In particular we have evaluated: neutrophils (CD45^+^/CD68^−^/F480^−^/Ly6G^+^) and their sub-populations (CD206^+^ or CD206^−^); monocytes (CD45^+^/CD68^+^/F480^−^) and their sub-populations (Ly6C^low^, Ly6C^high^, CD206^+^), macrophages (CD45^+^/CD68^+^/F480^+^) and their sub-populations (Ly6C^low^, Ly6C^high^, CD206^+^), mast cells (CD45^+^/CD117^+^), B cells (CD45^+^/CD19^+^), T cells (CD45^+^/CD3^+^). CCR2^+^ cells were also evaluated within the CD68^+^ monocyte/macrophage population. Immune cell apoptosis has been evaluated using an Annexin V (AV) antibody.

TgGRK5 overexpression did not affect leukocyte recruitment to the heart in either sham or RA of post-MI mice at 4-days and 8-weeks post-surgery. However, TgGRK5 overexpression significantly enhanced the infiltration of CD45^+^ leukocytes to the heart at both 4-days and 8-weeks post-MI in the BZ and infarct area compared to NLC-MI hearts (*Figures 4A* and *5A* and Supplementary material online, *Figures S14A* and *S20A*). In particular, TgGRK5 mice had increased infiltration of MPO^+^ neutrophils and CD68^+^ monocytes/macrophages into the BZ and infarct area of the heart when compared to NLC-HF mice at 4-days post-MI (*Figure 4B,C* and Supplementary material online, *Figure S14B, C*). Infiltration of tryptase^+^ mast cells and CD3^+^ T cells was not different between MI groups at 4-days post-surgery (*Figure 4D* and Supplementary material online, *Figure S14D–F*). FACS analysis did not show differences in leukocyte (*Figure 4E*) or neutrophil (*Figure 4G* and Supplementary material online, *Figure S16B*) levels in the hearts of TgGRK5 mice compared to NLC mice at 4 days post-MI. However, TgGRK5-MI hearts showed increased level of CD206^−^ neutrophils and decreased level of CD206^+^ neutrophils compared to NLC-MI hearts (*Figure 4H,I* and Supplementary material online, *Figures S16D* and *S17B*). Flow-cytometric quantification also highlighted the increase in macrophages (mainly Ly6C^hi^ macrophages) (*Figure 4K–N* and Supplementary material online, *Figures S16I–L* and *S17F–H*) and mast cells (*Figure 4R* and Supplementary material online, *Figure S16N*) but no difference in monocytes (as well as their sub-populations) (*Figure 4J* and Supplementary material online, *Figures S16E–H, S17C–E, S18A–C*), B cells (Supplementary material online, *Figures S16O* and *S17I*) and T cells (Supplementary material online, *Figures S16P* and *S17J*) in TgGRK5-MI hearts compared to NLC-MI hearts at an early time-point post-ischemic injury. CCR2^+^ monocyte/macrophages were trending towards increase in TgGRK5-MI hearts (*Figure 4O*). As TgGRK5 hearts showed increased cell death as well as leukocyte recruitment within the BZ compared NLC hearts at 4 days post-MI, we decided to evaluate whether immune cell apoptosis was affected by myocardial GRK5 overexpression. We discovered a trend towards increase in apoptotic leukocyte and increased apoptotic macrophages (when normalized for single cells or leukocytes) in TgGRK5 hearts compared to NLC post-MI hearts at 4 days post-MI (*Figure 4F,P* and Supplementary material online, *Figure S16T*). However, our data suggest this finding to be mainly attributable to the augmented number of macrophages within TgGRK5 heart rather then increased apoptotic rate in the macrophages. In fact, no difference was found between TgGRK5 and NLC post-MI hearts when the number of apoptotic macrophages was normalized over the number of macrophages (*Figure 4Q*). No difference in terms of apoptosis was found for other immune cells when we compared TgGRK5 and NLC hearts at 4 days post-MI (Supplementary material online, *Figure S16R–S, S16U–W, S17L–P, S18D–H*). Interestingly, GRK5 overexpression did not impact the production of cytokines, adhesion molecules or chemokines in either the BZ or scar area 4-days post-MI (Supplementary material online, *Figure S19*).

At a chronic phase of HF (8-weeks post-MI), TgGRK5 mice still displayed increased accumulation of leukocytes in both the infarcted area and BZ of the heart compared to NLC-MI heart (*Figure 5A* and Supplementary material online, *Figure S20A*). At this later time-point, mainly neutrophils and T-cells were highly recruited to the infarct area/BZ of TgGRK5 post-MI mice compared to NLC post-MI mice, while no changes were found in mast cell and monocyte/macrophage infiltration (*Figure 5B–D* and Supplementary material online, *Figure S20B–F*). As expected, FACS analysis showed reduced leukocyte levels and peculiar immune cell proportions within the cardiac tissue during the chronic compared to the acute phase post-MI in NLC mice (*Figures 4E–R* and *5E–K*; Supplementary material online, *Figures S16–S18, S21–S23*).^3^ Flow-cytometric quantification suggested a trend towards increase in CD45^+^ leukocyte (*Figure 5E*) levels and significantly increased neutrophil levels (*Figure 5F* and Supplementary material online, *Figure S22A*) in TgGRK5 hearts compared to NLC hearts at 4 weeks post-MI. Moreover, TgGRK5-MI hearts showed increased level of CD206^-^ neutrophils and decreased level of CD206^+^ neutrophils compared to NLC-MI hearts (*Figure 5G,H*; Supplementary material online, *Figures S21B* and *S22B*) as well as augmented levels of macrophages and monocytes (similarly Ly6C^hi^, Ly6C^hi^, and CD206^+^ sub-types) (*Figure 5I,J*; Supplementary material online, *Figures S21D–I* and *S23A–F*) at a chronic time-point post-ischemic injury. Flow-cytometric quantification confirmed the increase in T cells (*Figure 5K*) but no difference in mast cell and B cell levels (Supplementary material online, *Figure S21K, L*) or CCR2^+^ monocyte/macrophages (Supplementary material online, *Figure S23G*) in TgGRK5-MI hearts compared to NLC-MI hearts at 4 weeks post-MI. We found increased apoptotic leukocytes (mainly macrophages) in TgGRK5 hearts compared to NLC hearts at 4 weeks post-MI (Supplementary material online, *Figure S21M, P*). No difference was found between TgGRK5 and NLC post-MI hearts when the number of apoptotic leukocytes was normalized over the number of leukocytes or the number of apoptotic macrophages was normalized over the number of macrophages (Supplementary material online, *Figures S22P* and *S23J*).

Expression of inflammatory cytokines (IL-6 and IL-1β), VCAM1, and chemokines (CCL2, CCL3, CCL5) was increased in the post-MI LV of TgGRK5 mice compared to NLC mice at the end of the study, which could contribute at least in part to the long-lasting immune cell recruitment in the heart (*Figure 5L–O* and Supplementary material online, *Figure S24E, F*). Of note, CCL11 expression was downregulated in TgGRK5 hearts post-sham operation (at 4-days and 8-weeks post-MI) and 8-weeks post-MI compared to NLC groups, suggesting that myocardial GRK5 overexpression specifically reduces the levels of this chemokine in the LV (Supplementary material online, *Figures S19K* and *S24G*).

### 3.5 TgGRK2 mice did not show altered cardiac inflammation or immune cell recruitment in ischemic HF

Our lab has previously shown that cardiomyocyte overexpression of GRK2 has a detrimental effect on cardiac function, hypertrophy, and remodeling in ischemic heart disease.^4^^,^^18^ We wanted to assess whether myocardial transgenic overexpression of GRK2 affected the recruitment of leukocytes to the heart following MI. Accordingly, TgGRK2 and NLC (male and female) mice underwent surgical MI or sham operation and were followed for 8 weeks (Study design in Supplementary material online, *Figure S25A*). Consistent with previous studies, cardiac function was significantly impaired (reduced EF and FS as well as increased LV dimension) in TgGRK2 post-MI mice compared to NLC post-MI mice (*Figure 6A,B*; Supplementary material online, *Figure 25D,E* and *Table S4*).^18^ We found in the current study that myocardial GRK2 overexpression was particularly deleterious for the cardiac function and LV dimension of male mice while showing lower impact on female mice post-MI (Supplementary material online, *Figure S26A–D*). In line with diminished LV function, radial and longitudinal strain/strain rate were also impaired in TgGRK2 post-MI mice compared to NLC post-MI animals (Supplementary material online, *Figure S27A–F*). In fact, TgGRK2 post-MI mice showed impaired global as well as segmental radial and longitudinal strain and strain rate (Supplementary material online, *Figure S27A–F*). Cardiac hypertrophy (evaluated as HW, HW/BW, and HW/TL) was increased regardless of sex in TgGRK2 mice compared to NLC mice at 8 weeks post-MI (*Figure 6C*; Supplementary material online, *Figures S27G–H* and *S28A–F*).

As expected, cardiomyocyte-specific overexpression of GRK2 did not influence LV function and dimension as well as cardiac hypertrophy in sham mice compared to NLC sham animals (*Figure 6A–C*; Supplementary material online, *Figures S25B–C, S27G–H* and *Table S4*). GRK2 overexpression did not affect long-term survival rate in both male and female mice subjected to either MI or sham-operation (*Figur 6D*, Supplementary material online, *Figure S28G, H*).

Interestingly, GRK2 overexpression did not impact leukocyte infiltration in the heart at 4-days post-MI (*Figure 6E*). Hearts from TgGRK2mice did not have any changes in the recruitment of neutrophils, monocyte/macrophages, mast cells and T-cells in the RA, BZ and infarct area compared to NLC hearts at 4-days post-MI (*Figure 6F–I*). In contrast with our findings in TgGRK5 mice, no changes were found in Bex1, Cxcl11, Cxcl13 and Il-15 expression in the LV of TgGRK2 mice compared to NLC at 4 days post-MI (*Figure 6J*). Remarkably, we found that GRK2 overexpression led to reduced expression of IL-6, CCL2, and IL-18 in the LV samples at 8-weeks post-MI (*Figure 6K*). We believe that decreased chronic cardiac inflammation may counteract other deleterious mechanisms activated in TgGRK2 post-MI hearts, such as impaired β-AR and insulin signaling.^4^

### 3.6 GRK5cKO showed reduced immune cell recruitment to the heart, improved contractility and decreased mortality post-MI

As above, we subjected GRK5cKO mice and WT control (both male and female) mice to MI and studied these groups alongside sham-operated mice for 8 weeks (Supplementary material online, *Figure S29A*). Serial echocardiography showed gradual decrease of cardiac contractility post-MI in WT mice over the time while GRK5cKO mice did not display any further drop in contractility after 4 weeks post-MI and demonstrated significantly higher EF and FS compared to WT mice at 8 weeks post-MI (*Figure 7A* and Supplementary material online, *Figure S29D–E, Table S5*). Cardiomyocyte-specific deletion of GRK5 did not influence LV diameters/volumes in systole or diastole both in sham and post-MI mice compared to surgery-matched WT groups (*Figure 7B* and Supplementary material online,*Table S5*). We found in the current study that myocardial GRK5 deletion was particularly beneficial for the cardiac contractility of female mice post-MI while male GRK5cKO mice showed a trend towards increase in the EF and towards decrease in the LVIDd when compared to WT mice post-ischemic injury (Supplementary material online, *Figure S30*). No difference in cardiac function between sham groups was found at any time-point assessed (*Figure 7A*, Supplementary material online, *Figure 29B–C*, *Table S5*). In vivo speckle-tracking echocardiography confirmed enhanced LV function in GRK5cKO compared to WT mice post-MI and demonstrated that the myocyte GRK5 loss mainly preserved radial contractility post-MI (globally and more specifically in the posterior segments) (Supplementary material online, *Figure S31A–F*). Cardiomyocyte-specific deletion of GRK5 did not influence cardiac hypertrophy in post-sham or -MI mice from both sexes compared to surgery-matched WT groups (*Figure 7C*; Supplementary material online, *Figures S31G–H* and *32A–F*). We have evaluated the apoptosis in GRK5cKO hearts at an early time-point post-ischemic injury and have found a trend towards decrease in the number of TUNEL positive cardiomyocytes in the BZ of GRK5cKO hearts compared to WT hearts at 4 days post-MI (Supplementary material online, *Figure S33*). Moreover, GRK5cKO hearts showed an increase in vessels/cardiomyocyte ratio in the RA compared to WT hearts while no difference was found between these groups in terms of infarct length at 8 weeks post-MI (Supplementary material online, *Figures S34 and S35*).

Interestingly, myocardial GRK5 deletion reduced the infiltration of CD45^+^ leukocytes to the heart at 4-days post-MI in the BZ compared to WT-MI hearts (*Figure 7D*). GRK5cKO mice showed decreased infiltration of CD68^+^ monocytes/macrophages in the BZ and a trend towards decrease in MPO^+^ neutrophils infiltration in the BZ when compared to WT mice at 4-days post-MI (*Figure 7E,F*). Differences between MI groups in terms of immune cell recruitment into the infarct area were less pronounced (Supplementary material online, *Figure S36A–C*). Infiltration of tryptase^+^ mast cells and CD3^+^ T cells was not different between GRK5cKO and WT mice subjected either to MI or sham groups at 4-days post-surgery (Supplementary material online, *Figure S36D–G*).

Our FACS analysis did not show differences in leukocytes, neutrophils, macrophages, mast cells, B cells and T cells but displayed significantly decreased monocyte levels within the cardiac tissue in GRK5cKO mice compared to WT mice at 4 days post-MI (*Figure 7G,H,K,L*; Supplementary material online, *Figures S37* and *S38*). This finding may suggest monocytes as the CD68^+^ immune cell population that was found to be less recruited in the BZ of GRK5cKO mice. Interestingly, monocyte sub-populations were proportionally reduced while macrophages sub-populations as well as CCR2^+^ monocyte/macrophages were not changed in GRK5cKO hearts compared to WT hearts at 4 days post-MI (Supplementary material online, *Figures S37D–I* and *S39A–G*). Moreover, we discovered a trend towards decrease in CD206^−^ neutrophils and towards increase in the levels of CD206^+^ neutrophils in GRK5cKO hearts compared to WT hearts at an early time-point post-ischemic injury (*Figure 7I,J*). Finally, we found a trend towards decrease in the number of apoptotic T cells while no difference in the apoptosis of other immune cells in GRK5cKO hearts compared to WT hearts at 4 days post-MI (Supplementary material online, *Figures S37–S39*).

We evaluated the expression of immunomodulatory factors (Bex1, Cxcl11, Cxcl13, and IL-15) in post-MI cardiomyocytes and LV-sham samples from GRK5cKO mice compared to WT mice at 4 days post-surgery (Supplementary material online, *Figure S40*). A trend towards reduction in Cxcl13 while no difference in the other immune factors was found in GRK5cKO samples compared to NLC samples in both post-MI and sham mice. In line with improved global/segmental LV contractility and reduced leukocyte migration to the heart post-MI, GRK5cKO mice displayed decreased long-term mortality (with this difference being manifest in male but not female mice) when compared to WT mice post-MI (*Figure 7M* and Supplementary material online, *Figure S41*).

## 4. Discussion

The data described in this study greatly enhance the knowledge regarding the role of GRK5 in the pathogenesis of HF as it uncovers a new mechanism upon which regulation of GRK5 during myocardial ischemic injury affects ventricular function and survival (summary in Graphical Abstract). Cardiomyocyte-specific GRK5 expression not only influences global and segmental cardiac contractility but also effects cardiac immune cell recruitment in a mouse model of ischemic HF. GPCRs act as regulators of cardiac function in both physiology and pathology, and are widely recognized as one of the primary targets for current and future HF therapies.^4^^,^^26^ The canonical role of GRKs is to phosphorylate activated receptors inducing their desensitization, downregulation, and recycling.^4^^,^^26^ Interestingly, GRK5 has also the peculiar ability to move to the nucleus, where it induces hypertrophic gene expression and contributes to maladaptive hypertrophy.^9^^,^^10^^,^^12^ Both GRK5 and GRK2 have been found to be upregulated in human HF biopsies and a recent study has identified GRK5 as being crucial in the development of HF.^5^^,^^8^

We discovered that GRK5 is not only upregulated in the LV of mice post-MI, but is also specifically increased in cardiomyocytes at cytosolic, membrane and nuclear levels after ischemic injury. Hence, in order to understand whether myocardial GRK5 levels have a critical role in the development of ischemic HF, we studied the effects of MI in both TgGRK5 and GRK5cKO mice. We found that myocardial GRK5 upregulation severely impaired LV function and worsened LV remodeling, which can be partially attributed to the exaggerated early and late leukocyte infiltration to the damaged areas of the heart. Of note, TgGRK5 hearts not only showed increased levels of specific immune cell populations (macrophages and mast cells in the acute phase while neutrophils, macrophages and T cells in the chronic phase post-MI) but also peculiar levels of immune cell sub-populations. In particular, TgGRK5 hearts showed altered neutrophil sub-types (increased CD206^−^ and decreased CD206^+^ neutrophils compared to NLC hearts). This latest finding is in line with the worst cardiac phenotype in TgGRK5 mice as CD206^−^ neutrophils have been shown as pro-inflammatory while CD206^+^ neutrophils as anti-inflammatory during ischemic injury.^27^ Commonly described pro-inflammatory M1 macrophages (Ly-6C^hi^) were largely increased compared to reparative M2 (Ly-6C^lo^ or CD206^+^) in TgGRK5 hearts during the early phase post-MI.^24^^,^^27^ However, these latest data have to be carefully interpreted as the M1–M2 paradigm in the ischemic cardiomyopathy context has been challenged by recent evidences.^28^ No difference was found in terms of classical (Ly-6C^hi^) or non-classical monocytes (Ly-6C^lo^) in TgGRK5 hearts.

Our data show that TgGRK5 mice have reduced LV EF and segmental contractility as well as increased LV dimension. These results were corroborated by invasive hemodynamics. Of note, it has been previously shown that the GRK5-Leu41 mutation can affect β1-AR desensitization and impact the survival rate of African American HF patients by acting as a “genetic β-blockade”.^29^ Cardiac β-adrenergic inotropic reserve was not further compromised post-MI in TgGRK5 compared to NLC mice, suggesting that GRK5, unlike GRK2,^4^ is not interfering with β-AR downregulation and perhaps only the mutant form of GRK5 affects β-ARs in HF setting.^29^ In the current study, cardiac GRK5 overexpression led to the development of exaggerated hypertrophy, fibrosis, increased fetal gene expression, and reduced survival rate, which is the most important outcome in clinical studies. In agreement with the RNA-Seq pathway analysis and RCAN upregulation, cardiac hypertrophy in post-MI TgGRK5 mice may be the result of NFAT activation, as we have shown in a pressure-overload model.^10^ Of note, it might be also possible that NFAT (transcriptional factor controlling a wide range of genes) regulates fetal gene expression in TgGRK5-MI and -Sham hearts.^10^^,^^30^^,^^31^ Moreover, transcriptome unbiased analysis of post-MI cardiomyocytes with GRK5 overexpression showed altered levels of several genes previously indicated to be involved in cardiac hypertrophy, remodeling or HF such as Kcne1, Efna5, Psmd8 and several regulators of the extracellular matrix.^32–35^ Of note, we also found TgGRK5 post-MI cardiomyocytes to have downregulated expression of HDAC5, which could partially contribute to the cardiac hypertrophy/dysfunction as shown by HCAD5KO mice during stress.^36^

It has been reported that MMP2 activity in mice promptly increases within 4 days, peaks at day 7, and remains augmented until day 14 post-MI.^37^^,^^38^ However, TgGRK5 mice still show high LV MMP2 mRNA levels at 8 weeks post-MI and this may explain at least in part the increased mortality rate in TgGRK5 mice as MMP-2 has been linked to impaired ECM degradation and increased risk of cardiac rupture.^37^ Moreover, cardiac-specific constitutively active MMP2 expression has been shown to lead to impaired LV function and reduced inotropic response.^39^ Interestingly, infarct size/length was not changed, while apoptosis was only increased in the BZ (not in cardiomyocytes but most probably in infiltrating immune cells as apoptotic macrophages were particularly abundant) of TgGRK5 post-MI hearts. These data suggest cardiomyocyte GRK5 is acting differently from GRK2, which has been shown to also act as a pro-death kinase.^40^

Our results demonstrate for the first time that cardiomyocyte-specific transgenic overexpression of GRK5 not only enhanced the acute immune cell recruitment response post-MI to the heart (macrophages, mast cells, and pro-inflammatory neutrophils) but also led to chronic immune cell infiltration after the establishment of HF (neutrophils, macrophages, and T-cells). The altered expression of several regulators of the immune system/inflammation in TgGRK5 cardiomyocytes may have played a critical role in the exaggerated leukocyte accumulation into the injured heart. In particular, Bex1 may have crucially contributed as it was found as the top upregulated gene in TgGRK5 cardiomyocytes and has been recently described to act as chemoattractor of immune cells during cardiac injury.^41^

Bex1 expression is epigenetically regulated and its overexpression in TgGRK5 hearts may be at least in part related to HDAC downregulation (TgGRK5 cardiomyocytes showed reduced expression of both HDAC5 and HDAC6 post-MI) as previously shown for Bex family genes in a different setting.^42^^,^^43^ Interestingly, TgGRK5 cardiomyocytes also showed dysregulated expression of several immune-related genes, including Cxcl11, Cxcl13, and Il-15, that have been previously associated to leukocyte migration and to cardiac dysfunction and remodeling during heart disease.^44–46^ A long-lasting dysregulated immune response in the heart promotes adverse cardiac remodeling, which eventually progresses to HF.^2^ This was the case in TgGRK5 mice that had persistent leukocyte infiltration and chronic inflammation. Our results are also in agreement with previous studies showing that GRK5 acts as regulator of inflammation/immune cell migration in response to LPS- and TLR-stimulation.^9^^,^^13^

TgGRK2 mice did not present with the same phenotype and outcome as TgGRK5 mice, which is interesting. In fact, TgGRK2 failing (8 weeks post-MI) mice had decreased cardiac function but lower cardiac hypertrophy compared to TgGRK5 failing mice, and no changes in survival compared to NLC failing mice. Our study also indicates that the pathology evoked by GRK2 upregulation is different than the pathology induced by GRK5. In fact, immune cell migration to the heart and LV levels of several immuno-related genes were not increased in TgGRK2 post-MI. As we have previously shown, the detrimental effects of cardiac GRK2 upregulation on cardiac contractile function after ischemic injury rely on dysfunctional β-AR signaling, increased myocyte death, and impaired insulin signaling and glucose uptake.^4^^,^^40^

Being of the male sex has been shown to be an independent risk factor for HF with reduced EF, however women are also affected by heart disease despite the protective role of circulating estrogens.^47^ Our study suggests myocardial GRK5 but not GRK2 overexpression to cause dramatic cardiac dysfunction and mortality not only in male but also in female mice post-MI.

Importantly, GRK5cKO mice were protected from ischemic injury in terms of both global and segmental contractility as well as long-term mortality. Deleting GRK5 gene in cardiomyocytes reduced early leukocyte migration (mainly monocytes) to the heart with CD68^+^ cells significantly less infiltrating in the BZ post-MI. The minor reduction in Cxcl13 levels may contribute to the blunted cardiac immune phenotype in GRK5cKO mice post-MI but we cannot exclude other factors being involved. Moreover, GRK5cKO hearts did not show differences in infarct length but displayed a trend towards decrease in apoptotic cardiomyocyte and improved angiogenesis post-ischemic injury compared to WT hearts.

Our findings support the potential benefit of GRK modulation in clinical studies and selective small molecule inhibitors of GRK5 have begun to emerge as novel therapeutic treatment in HF.^48–50^ However, further in-deep studies must be performed to assess if GRK5 pharmacological inhibition can ameliorate cardiac function, remodeling and outcomes in ischemic HF. In addition, our data suggest that it would be also interesting to test pan-GRK2/5 inhibitors in the ischemic HF phenotype as both GRKs are upregulated and deleterious in HF and we have found GRK2 to be even further upregulated in TgGRK5 post-MI mice.^51^

Our study shows for the first time that myocardial GRK5 has a crucial role on the regulation of cardiac function and remodeling as well as survival in a murine model of ischemic HF. Importantly, we discovered that cardiomyocyte GRK5 levels can affect immune cell recruitment to the heart after ischemic injury. Our results indicate that GRK5 may be a potential therapeutic target for limiting the development of ischemic HF.

## 5. Study limitations

Our study did not evaluate if the detrimental role of cardiomyocyte GRK5 in ischemic HF is related to its canonical vs. non-canonical activity or kinase vs. non-kinase activity. Further studies must be performed to clarify the specific cellular mechanisms involved.

## Supplementary material

Supplementary material is available at *Cardiovascular Research* online.

## Authors’ contribution

C.d.L. and W.J.K. designed the study, developed the experimental design, interpreted the experiments and wrote the manuscript. C.d.L., L.A.G., G.B., M.P., J.I., A.M.L., E.W.B., R.R., A.D.O., and H.C.M. performed the experiments. E.G. performed the surgeries. C.d.L., L.A.G., and MP performed data analyses. G.R., S.R.H., and DGT edited the manuscript. All authors discussed the results and approved the manuscript.

## Supplementary Material

cvab044_Supplementary_Data

## Notes

Translational perspective GRK5 is upregulated in failing human myocardium and associated with heart failure development. In this study, we evaluated whether cardiomyocyte GRK5 plays a critical role during ischemic heart disease in a mouse animal model. We discovered that GRK5 overexpression in cardiomyocyte affects cardiac function, remodeling, immune cell recruitment, and ultimately survival in ischemic heart failure. Conversely, cardiomyocyte-specific GRK5 ablation diminished the early immune cell infiltration in the heart, improved contractility and reduced mortality post-myocardial infarction. The overall translational significance of these findings is substantial, as selective small molecule inhibitors of GRK5 have begun to emerge as novel therapeutic treatment in heart disease.
