# Supplementary material for: G protein-coupled receptor kinase 5 (GRK5) contributes to impaired cardiac function and immune cell recruitment in post-ischemic heart failure
Source: Cardiovasc Res. 2021 Feb 9;118(1):169–83. doi: 10.1093/cvr/cvab044 (PMC8752360; doi:10.1093/cvr/cvab044)

SUPPLEMENTARY DATA

GRK5 contributes to impaired cardiac function and immune cell recruitment in post-ischemic heart failure.

Short title: Myocardial GRK5 in post-ischemic heart failure.

Claudio de Lucia, MD, PhD[a], Laurel A. Grisanti, PhD[b], Giulia Borghetti, PhD[c], Michela Piedepalumbo, MD[a,d], Jessica Ibetti, PhD[a], Anna Maria Lucchese, MSc[a], Eric W. Barr, BS[a], Rajika Roy, PhD[a], Ama Dedo Okyere, MPH[a], Haley Christine Murphy, BS[a], Erhe Gao MD, PhD[a], Giuseppe Rengo MD, PhD[e,f], Steven R. Houser, PhD[c], Douglas G. Tilley, PhD[a], and Walter J. Koch, PhD[a]

[a]Center for Translational Medicine, Lewis Katz School of Medicine, Temple University, Philadelphia, PA, USA; [b]Department of Biomedical Sciences, College of Veterinary Medicine, University of Missouri, Columbia, Missouri, USA; [c]Cardiovascular Research Center, Lewis Katz School of Medicine, Temple University, Philadelphia, PA, USA; [d]Department of Medical, Surgical, Neurological, Metabolic and Aging Sciences, University of Campania "Luigi Vanvitelli", Naples, Italy; [e]Department of Translational Medical Sciences, Division of Geriatrics, Federico II University, Via S. Pansini, 5, Naples 80131, Italy; [f]Laboratory of neurovegetative system pathophysiology, Istituti Clinici Scientifici ICS Maugeri, IRCCS Istituto Scientifico di Telese Terme, Benevento, Italy.

Address for correspondence:
Walter J. Koch, Ph.D.
Center for Translational Medicine
Lewis Katz School of Medicine,
Temple University
3500 N. Broad Street; 940-MERB
Philadelphia, PA, US, 19140.
Phone: +1-215-707-9820;
Fax: +1-215.707.9890
Email: walter.koch@temple.edu

Co-corresponding author (address):
Claudio de Lucia, M.D., Ph.D.
Center for Translational Medicine
Lewis Katz School of Medicine,
Temple University
3500 N. Broad Street; 980-MERB
Philadelphia, PA, US, 19140.
Phone: +1- 215-707-5045
Fax: +1-215.707.9890
Email: claudio.de.lucia@temple.edu

SUPPLEMENTARY FIGURE LEGENDS:

Suppl. Figure 1. Representative Western blot **(A)** and quantification **(B)** showing GRK5 protein levels from left ventricular lysates in WT mice at 4 days post-Sham operation or myocardial infarction (MI). GAPDH was used as loading control. n=7 per group. Representative Western blot **(C)** and quantification **(D)** showing GRK5 protein levels in different cell fractions (C=cytosolic fraction; M=membrane fraction; N=nuclear fraction) from cardiomyocytes isolated from WT mice at 3-days post-Sham or -MI. Total protein stain used as loading control. GAPDH, Na^+/K^+-ATPase-α and Histone H1 used as fraction purity control (n=3-5). *p<0.05, **p<0.01, ***p<0.001. Unpaired T-test or Mann-Whitney test were used between groups.

Suppl. Figure 2. Representative parasternal short axis echocardiograms in M-mode at 8-weeks post-surgery for each study group: NLC-Sham **(A)**, TgGRK5-Sham **(B)**, NLC-MI **(C)** and TgGRK5-MI **(D)**. X-axis = seconds; y-axis = millimeters.

Suppl. Figure 3. Ejection fraction (EF) **(A and C)** and left ventricular internal diameter in diastole (LVIDd) **(B and D)** as measured by echocardiography at 0 (baseline), 4 and 8 weeks post-sham operation or myocardial infarction (MI) in both male and female mice from NLC and TgGRK5 groups (n=4-24). #p<0.0001 vs NLC-Sham and TgGRK5-Sham, ^p<0.0001 vs NLC-Sham and p<0.001 TgGRK5-Sham, ^^p<0.001 vs NLC-Sham and TgGRK5-Sham, *p<0.05, **p<0.01,***p<0.001, ****p<0.0001. Two-way ANOVA with Tukey's post hoc test was used between groups.

Suppl. Figure 4. Representative pictures of left ventricular (LV) deformation (green lines) during the cardiac cycle along the radial and longitudinal axes (parasternal long axis view) at 8 weeks post-myocardial infarction (MI) for NLC **(A)**, and TgGRK5 **(B)** groups. Y-axis = millimeters. Radial and longitudinal strain or strain rate measured at six LV segments as well as

their average at 8 weeks post-MI in NLC and TgGRK5 groups **(C-F)** (n= 26-31). *p<0.05, **p<0.01. T-test or Mann-Whitney test were used between groups.

Suppl. Figure 5. Measures of heart weight (HW) **(A)** and HW to tibia length ratio (HW/TL) **(B)** (n= 15-27) in NLC and TgGRK5 mice at 8 weeks post-Sham operation or myocardial infarction (MI). *p<0.05 **p<0.01, ****p<0.0001, #p<0.0001 vs NLC-Sham and TgGRK5-Sham. One-way ANOVA with Tukey's post hoc test.

Suppl. Figure 6. Measures of heart weight (HW) **(A and D)**, HW to body weight ratio (HW/BW) **(B and E)** and HW to tibia length ratio (HW/TL) **(C and F)** at 8 weeks post-sham operation or myocardial infarction (MI) in both male and female mice from NLC and TgGRK5 groups (n=6-16). *p<0.05, **p<0.01, ***p<0.001, ****p<0.0001. One-way ANOVA with Tukey's post hoc test was used between groups.

Suppl. Figure 7. Quantification **(A)** and representative Western blot **(B)** showing GRK2 protein levels from left ventricular samples in mice from NLC and TgGRK5 groups at 4 days post-Sham or post-MI. GAPDH used as loading control (n=7 per group). **p<0.01, ***p<0.001. One-way ANOVA with Tukey's post hoc test.

Suppl. Figure 8. Transcriptome evaluated in cardiomyocytes isolated 4 days post-myocardial infarction from TgGRK5 mice compared to NLC mice (5 mice per group). Nine selected networks found by Ingenuity Pathway Analysis among genes significantly affected by Grk5 overexpression. **A**: Cellular Development, Cellular Growth and Proliferation, Embryonic Development. **B:** Small Molecule Biochemistry, Humoral Immune Response, Protein Synthesis. **C:** Cellular Compromise, Hypersensitivity Response, Inflammatory Response. **D:** Bradycardia, Cardiac Arrhythmia, Cardiovascular Disease. **E:** Lipid Metabolism, Molecular Transport, Small Molecule Biochemistry. **F:** Cell Cycle, Cellular Movement, Carbohydrate Metabolism. **G:**

Cellular Movement, Hematological System Development and Function, Immune Cell Trafficking. **H:** Metabolic Disease, Organismal Injury and Abnormalities, Amino Acid Metabolism. **I:** Cardiovascular Disease, Cell Death and Survival, Cellular Assembly and Organization.

Suppl. Figure 9. Quantification of RT-PCR data showing fold change of NLC-Sham for Regulator of calcineurin (RCAN) in left ventricular (LV) samples from NLC and TgGRK5 groups at 4 days post-sham operation or myocardial infarction (MI) **(A)** (n=6-11). Quantification of RT-PCR data showing fold change of NLC-Sham for Bex1, Cxcl11, Cxcl13 and IL-15 **(B)** in left ventricular (LV) samples from NLC and TgGRK5 groups at 4 days post-sham operation (n=5-8). *$p<0.05$, **$p<0.01$. One-way ANOVA with Tukey's post hoc test, T-test or Mann Whitney test were used between groups.

Suppl. Figure 10. Representative TUNEL staining (red) of left ventricular (LV) tissue from NLC and TgGRK5 mice at the remote area (RA), border zone (BZ) and infarct area counterstained with Troponin T (green) to visualize cardiomyocytes, at 24 hours post-myocardial infarction (MI) **(A-F)**; DAPI was used as a nuclear marker (blue). Representative TUNEL staining (red) of LV tissue from NLC and TgGRK5 mice at RA and infarct area counterstained with Troponin T (green) to visualize cardiomyocytes, at 4 days post-MI **(G-J)**; DAPI was used as a nuclear marker (blue). Scale bar in white (500µm). Quantification of TUNEL positive cardiomyocytes **(K)** from NLC and TgGRK5 mice at the border zone (BZ), at 4 days post-MI (n=6-12). Mann-Whitney test was used among groups. Representative images of Picrosirius red staining in LV tissue from NLC and TgGRK5 mice at 8-weeks post-Sham operation or MI (at RA) **(L-O)**. Scale bar in black (500µm).

Suppl. Figure 11. Representative CD31 staining (red) of left ventricular tissue from NLC and TgGRK5 mice at the remote area (RA) counterstained with Troponin T (green) to visualize cardiomyocytes, at 8 weeks post-myocardial infarction (MI) **(A)**; DAPI was used as a nuclear marker (blue). Scale bar in white (100μm). Quantification of vessels/cardiomyocytes ratio **(B)** from NLC and TgGRK5 mice at the RA, at 8 weeks post-MI (n=4 per group). Mann-Whitney test was used among groups.

Suppl. Figure 12. Representative Masson's trichrome staining **(A)** and quantification of infarct length (% of left ventricle – LV) **(B)** for hearts from NLC and TgGRK5 mice at 8 weeks post myocardial infarction (MI). Scale bar in black (1000μm). n=4-5. T-test was used among groups.

Suppl. Figure 13. Kaplan-Meier survival curves of male **(A)** and female **(B)** mice from NLC and TgGRK5 groups subjected to sham operation or myocardial infarction (MI) (n=4-73). *$p<0.05$. Log-rank (Mantel-Cox) test has been used between groups.

Suppl. Figure 14. Representative images and quantification of CD45 **(A)** MPO **(B)**, CD68 **(C)**, tryptase **(D)** and CD3 **(E)** stainings in Sham hearts as well as infarct area of hearts post-myocardial infarction (MI). Representative images and quantification of CD3 **(F)** in remote area (RA) and border zone (BZ) of hearts post-MI. NLC and TgGRK5 groups evaluated at 4 days post-surgery (n=5-11). Scale bar in white (100μm). Arrowheads indicate positive staining. Insets show higher magnification at 250×. *$p<0.05$, **$p<0.01$, ***$p<0.001$, ****$p<0.0001$. One-way ANOVA with Tukey's post hoc test was used between groups.

Suppl. Figure 15. Gating strategy for FACS analysis. Representative gating strategy of mouse immune cells, isolated from the heart, is shown. Cells were gated for singlet cells (FSC-H vs. FSC-A) and then analyzed for their uptake of the Live/Dead (L/D) stain to determine live versus dead cells **(A)**. Alive cells were further gated to obtain the CD45+ population **(A)**. CD45+ cells

were then gated to detect the expression of: Annexin V, CD68, F4/80, Ly6G, CD206, Ly6C, CCR2, CD19, CD3, CD117 **(A-G)**.

Suppl. Figure 16. Flow-cytometric quantification of immune cells isolated from NLC and TgGRK5 hearts at 4 days post-MI **(A-W)** (n=7 per group). Specific immune cell populations have been expressed as % of single cells. AV= Annexin V. *p<0.05. T-test or Mann-Whitney test were used between groups.

Suppl. Figure 17. Flow-cytometric quantification of immune cells isolated from NLC and TgGRK5 hearts at 4 days post-MI **(A-P)** (n=7 per group). Specific immune cell populations have been expressed as % of $CD45^+$ cells. AV= Annexin V. *p<0.05, **p<0.01. T-test or Mann-Whitney test were used between groups.

Suppl. Figure 18. Flow-cytometric quantification of immune cells isolated from NLC and TgGRK5 hearts at 4 days post-MI **(A-H)** (n=7 per group). AV= Annexin V. Specific immune cell sub-populations have been analyzed. T-test was used between groups.

Suppl. Figure 19. Quantification of RT-PCR data showing fold change of NLC-Sham for TNFα, IL-6, IL-1β, IL-18, IL-33, ICAM1, VCAM1, CCL2, CCL3, CCL5, CCL11, CXCL1, CXCL2, CXCL5 and IFNγ in left ventricular samples from NLC and TgGRK5 mice collected at 4 days post-surgery (n=5-15). *p<0.05, **p<0.01, ***p<0.001, ****p<0.0001. One-way ANOVA with Tukey's post hoc test was used between groups.

Suppl. Figure 20. Representative images and quantification of CD45 **(A)**, MPO **(B)**, CD68 **(C)**, tryptase **(D)** and CD3 **(E)** stainings in Sham hearts as well as border zone (BZ) of post-myocardial infarction (MI) hearts. Representative images and quantification of tryptase **(F)** staining in remote area (RA) and infarct area of post-MI hearts. NLC and TgGRK5 groups evaluated at 8 weeks post-surgery (n=5-10). Scale bar in black (100μm). Arrowheads indicate

positive staining. Insets show higher magnification at 250×. *p<0.05, **p<0.01, ***p<0.001, ****p<0.0001. One-way ANOVA with Tukey's post hoc test was used between groups.

Suppl. Figure 21. Flow-cytometric quantification of immune cells isolated from NLC and TgGRK5 hearts at 4 weeks post-MI **(A-S)** (n=8 per group). Specific immune cell populations have been expressed as % of single cells. AV= Annexin V. *p<0.05, **p<0.01. T-test or Mann-Whitney test were used between groups.

Suppl. Figure 22. Flow-cytometric quantification of immune cells isolated from NLC and TgGRK5 hearts at 4 weeks post-MI **(A-V)** (n=8 per group). AV= Annexin V. Specific immune cell populations have been expressed as % of $CD45^+$ cells. *p<0.05. T-test or Mann-Whitney test were used between groups.

Suppl. Figure 23. Flow-cytometric quantification of immune cells isolated from NLC and TgGRK5 hearts at 4 weeks post-MI **(A-M)** (n=8 per group). AV= Annexin V. Specific immune cell sub-populations have been analyzed. T-test or Mann-Whitney test were used between groups.

Suppl. Figure 24. Quantification of RT-PCR data showing fold change of NLC-Sham for TNFα, IL-18, IL-33, ICAM1, CCL3, CCL5, CCL11, CXCL1, CXCL2, CXCL5 and IFNγ, **(A-K)** in left ventricular samples collected from NLC and TgGRK5 mice at 8 weeks post-surgery (n=3-18). *p<0.05, ***p<0.001, ****p<0.0001. One-way ANOVA with Tukey's post hoc test was used between groups.

Suppl. Figure 25. A: Overall design of the 8-week study comparing NLC and TgGRK2 mice post-myocardial infarction (MI) or Sham operation. Representative parasternal short axis echocardiograms in M-mode at 8-weeks post-surgery for each study group: NLC-Sham **(B)**, TgGRK2-Sham **(C)**, NLC-MI **(D)** and TgGRK2-MI **(E)**. X-axis = seconds; y-axis = millimeters.

Suppl. Figure 26. Ejection fraction (EF) **(A and C)** and left ventricular internal diameter in diastole (LVIDd) **(B and D)** as measured by echocardiography at 0 (baseline), 4 and 8 weeks post-sham operation or myocardial infarction (MI) in both male and female mice from NLC and TgGR2 groups (n=3-10). #p<0.0001 vs TgGRK2-Sham and p<0.001 vs NLC-Sham, ##p<0.0001 vs NLC-Sham and TgGRK2-Sham, ^p<0.01 vs NLC-Sham and TgGRK2-Sham, ^^p<0.001 vs NLC-Sham and TgGRK2-Sham, &p<0.0001 vs TgGRK2-Sham and p<0.01 vs NLC-Sham, $p<0.01 vs TgGRK2-Sham and p<0.05 vs NLC-Sham, &&p<0.05 vs TgGRK2-Sham and NLC-Sham, $$p<0.05 vs TgGRK2-Sham. *p<0.05, **p<0.01,***p<0.001. Two-way ANOVA with Tukey's post hoc test was used between groups.

Suppl. Figure 27. Representative pictures of left ventricular (LV) deformation (green lines) during the cardiac cycle along the radial and longitudinal axes (parasternal long axis view) at 8 weeks post-myocardial infarction (MI) for NLC **(A)**, and TgGRK2 **(B)** groups. Y-axis = millimeters. Radial and longitudinal strain or strain rate measured at six LV segments as well as their average at 8 weeks post-MI in NLC and TgGRK2 groups **(C-F)** (n=11-12 per group). Measures of heart weight (HW) **(G)** and HW to tibia length ratio (HW/TL) at 8 weeks post-Sham operation or MI **(H)** (n=6-13). *p<0.05, **p<0.01, ***p<0.001. One-way ANOVA with Tukey's post hoc test, T-test or Mann-Whitney test were used between groups.

Suppl. Figure 28. Measures of heart weight (HW) **(A and D)**, HW to body weight ratio (HW/BW) **(B and E)** and HW to tibia length ratio (HW/TL) **(C and F)** at 8 weeks post-sham operation or myocardial infarction (MI) in both male and female mice from NLC and TgGRK2 groups (n=3-7). *p<0.05, **p<0.01,***p<0.001, ****p<0.0001. One-way ANOVA with Tukey's post hoc test was used between groups. Kaplan-Meier survival curves of male **(G)** and

female **(H)** mice from NLC and TgGRK2 groups subjected to sham operation or myocardial infarction (MI) (n=4-36). Log-rank (Mantel-Cox) test was used between groups.

Suppl. Figure 29. A: Overall design of the 8-week study comparing WT and GRK5cKO mice post-myocardial infarction (MI) or Sham operation. Representative parasternal short axis echocardiograms in M-mode at 8-weeks post-surgery for each study group: WT-Sham **(B)**, GRK5cKO-Sham **(C)**, WT-MI **(D)** and GRK5cKO-MI **(E)**. X-axis = seconds; y-axis = millimeters.

Suppl. Figure 30. Ejection fraction (EF) **(A and C)** and left ventricular internal diameter in diastole (LVIDd) **(B and D)** as measured by echocardiography at 0 (baseline), 4 and 8 weeks post-sham operation or myocardial infarction (MI) in both male and female mice from WT and GRK5cKO groups (n=4-13). #p<0.0001 vs WT-Sham and p<0.001 vs GRK5cKO-Sham, ##p<0.01 vs WT-Sham and p<0.001 vs GRK5cKO-Sham, ^p<0.0001 vs WT-Sham and GRK5cKO-Sham, ^^p<0.01 vs WT-Sham and p<0.05 GRK5cKO-Sham, &p<0.05 vs GRK5cKO-Sham, &&p<0.05 vs WT-Sham and GRK5cKO-Sham, $p<0.01 vs WT-Sham and GRK5cKO-Sham, $$p<0.001 vs WT-Sham and GRK5cKO-Sham, **p<0.01. Two-way ANOVA with Tukey's post hoc test was used between groups.

Suppl. Figure 31. Representative pictures of left ventricular deformation (LV) (green lines) during the cardiac cycle along the radial and longitudinal axes (parasternal long axis view) at 8 weeks post-myocardial infarction (MI) for WT **(A)**, and GRK5cKO **(B)** groups. Y-axis = millimeters. Radial and longitudinal strain or strain rate measured at six LV segments as well as their average at 8 weeks post-MI in WT and GRK5cKO groups **(C-F)** (n=17-20). Measures of heart weight (HW) **(G)** and HW to tibia length ratio (HW/TL) at 8 weeks post-Sham operation or

MI **(H)** (n=20-24). *p<0.05, **p<0.01, #p<0.0001 vs WT-Sham and GRK5cKO-Sham. One-way ANOVA with Tukey's post hoc test, T-test or Mann-Whitney test were used between groups.

Suppl. Figure 32. Measures of heart weight (HW) **(A and D)**, HW to body weight ratio (HW/BW) **(B and E)** and HW to tibia length ratio (HW/TL) **(C and F)** at 8 weeks post-sham operation or myocardial infarction (MI) in both male and female mice from WT and GRK5cKO groups (n=6-15). #p<0.0001 vs WT-Sham and GRK5cKO-Sham. One-way ANOVA with Tukey's post hoc test was used between groups.

Suppl. Figure 33. Representative TUNEL staining (red) of left ventricular tissue from WT and GRK5cKO mice at the border zone (BZ) counterstained with Troponin T (green) to visualize cardiomyocytes, at 4 days post-myocardial infarction (MI) **(A)**; DAPI was used as a nuclear marker (blue). Scale bar in white (100μm). Quantification of TUNEL positive cells **(B)** from WT and GRK5cKO mice at the BZ, at 4 days post-MI (n=4-5). T-test was used among groups.

Suppl. Figure 34. Representative CD31 staining (red) of left ventricular tissue from WT and GRK5cKO mice at the remote area (RA) counterstained with Troponin T (green) to visualize cardiomyocytes, at 8 weeks post-myocardial infarction (MI) **(A)**; DAPI was used as a nuclear marker (blue). Scale bar in white (100μm). Quantification of vessels/cardiomyocytes ratio **(B)** from WT and GRK5cKO mice at the RA, at 8 weeks post-MI (n=5-6). *p<0.05. T-test was used among groups.

Suppl. Figure 35. Representative Masson's trichrome staining **(A)** and quantification of infarct length (% of left ventricle – LV) **(B)** for hearts from WT and GRK5cKO mice at 8 weeks post myocardial infarction (MI). Scale bar in black (1000μm). n= 6 per group. T-test was used among groups.

Suppl. Figure 36. Representative images and quantification of CD45 **(A)** MPO **(B)**, CD68 **(C)**, tryptase **(D)** and CD3 **(E)** staining in Sham hearts as well as infarct area of hearts post-myocardial infarction (MI). Representative images and quantification of tryptase **(F)** and CD3 **(G)** staining in remote area (RA) and border zone (BZ) of hearts post-myocardial infarction (MI). WT and GRK5cKO groups evaluated at 4 days post-surgery (n=5-13). Scale bar in white (100μm). Arrowheads indicate positive staining. Insets show higher magnification at 250×. *p<0.05, **p<0.01, ***p<0.001, ****p<0.0001. One-way ANOVA with Tukey's post hoc test was used between groups.

Suppl. Figure 37. Flow-cytometric quantification of immune cells isolated from WT and GRK5cKO hearts at 4 days post-MI **(A-T)** (n=4-8). Specific immune cell populations have been expressed as % of single cells. AV= Annexin V. *p<0.05. T-test or Mann-Whitney test were used between groups.

Suppl. Figure 38. Flow-cytometric quantification of immune cells isolated from WT and GRK5cKO hearts at 4 days post-MI **(A-V)** (n=4-8). Specific immune cell populations have been expressed as % of $CD45^+$ cells. AV= Annexin V. T-test or Mann-Whitney test were used between groups.

Suppl. Figure 39. Flow-cytometric quantification of immune cells isolated from WT and GRK5cKO hearts at 4 days post-MI **(A-M)** (n=4-8). Specific immune cell sub-populations have been analyzed. AV= Annexin V. T-test or Mann-Whitney test were used between groups.

Suppl. Figure 40. Quantification of RT-PCR data showing fold change of WT-MI for Bex1, Cxcl11, Cxcl13 and IL-15 in cardiomyocytes isolated 4 days post-myocardial infarction (MI) from WT and GRK5cKO groups (n=5-8) **(A)**. Quantification of RT-PCR data showing fold change of WT-Sham for Bex1, Cxcl11, Cxcl13 and IL-15 in left ventricular (LV) samples at 4

days post-sham operation (n=5-10) from WT and GRK5cKO groups **(B)**. T-test or Mann Whitney test were used between groups.

Suppl. Figure 41. Kaplan-Meier survival curves of male **(A)** and female **(B)** mice from WT and GRK5cKO groups subjected to sham operation or myocardial infarction (MI) (n=4-26). *p<0.05. Log-rank (Mantel-Cox) test has been used between groups.

Suppl. Figure 1

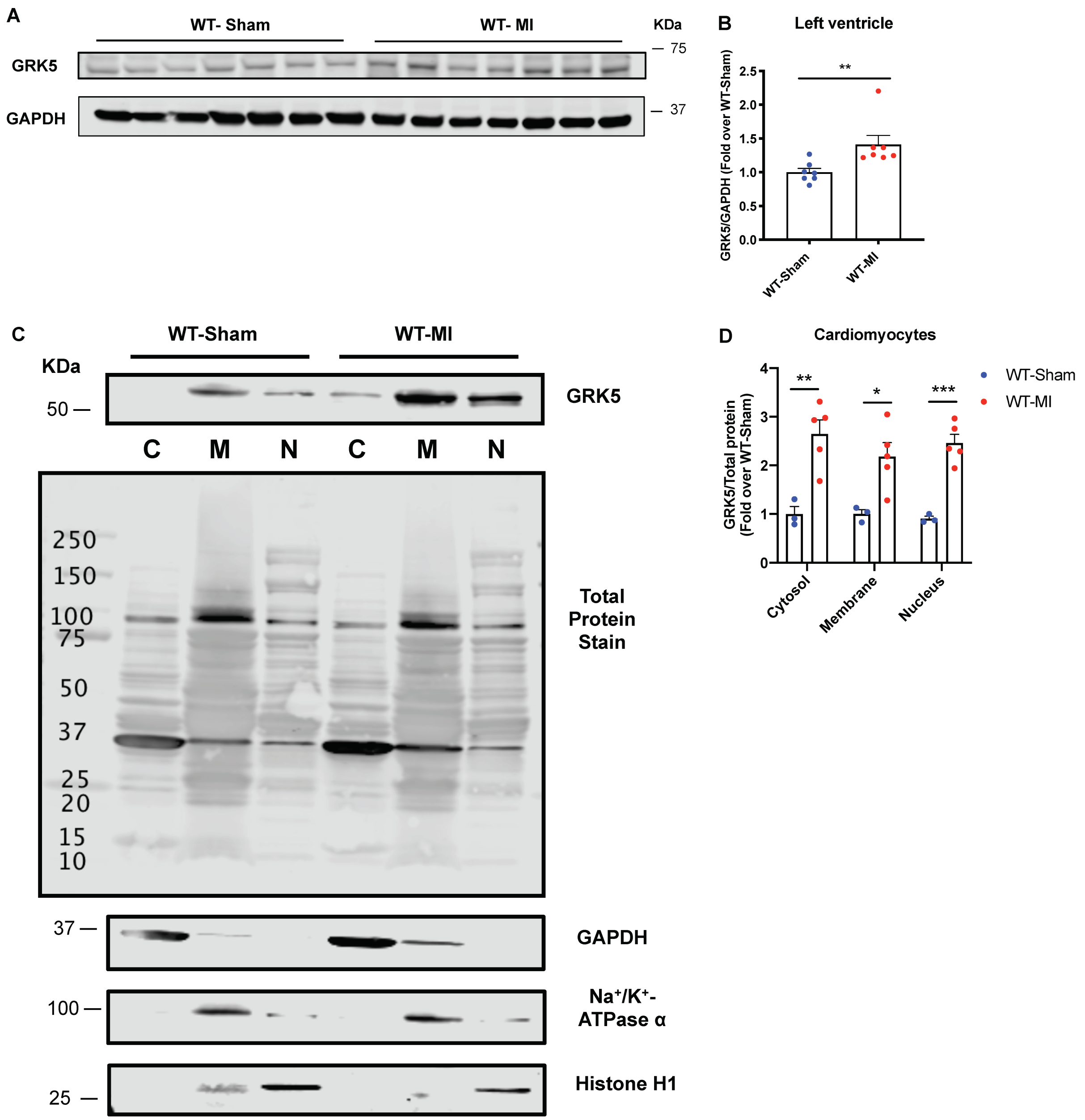

Suppl. Figure 2

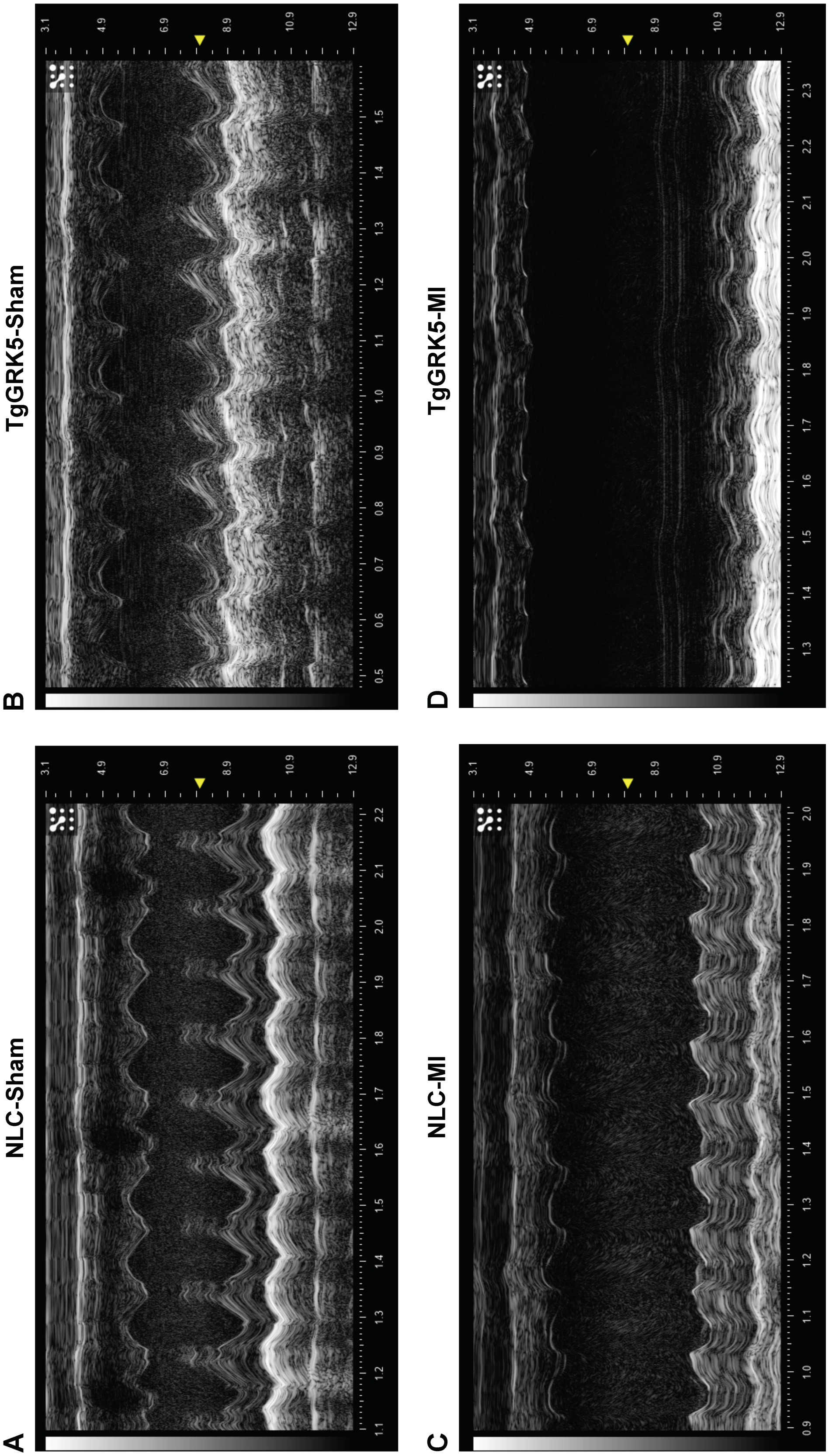

Suppl. Figure 3

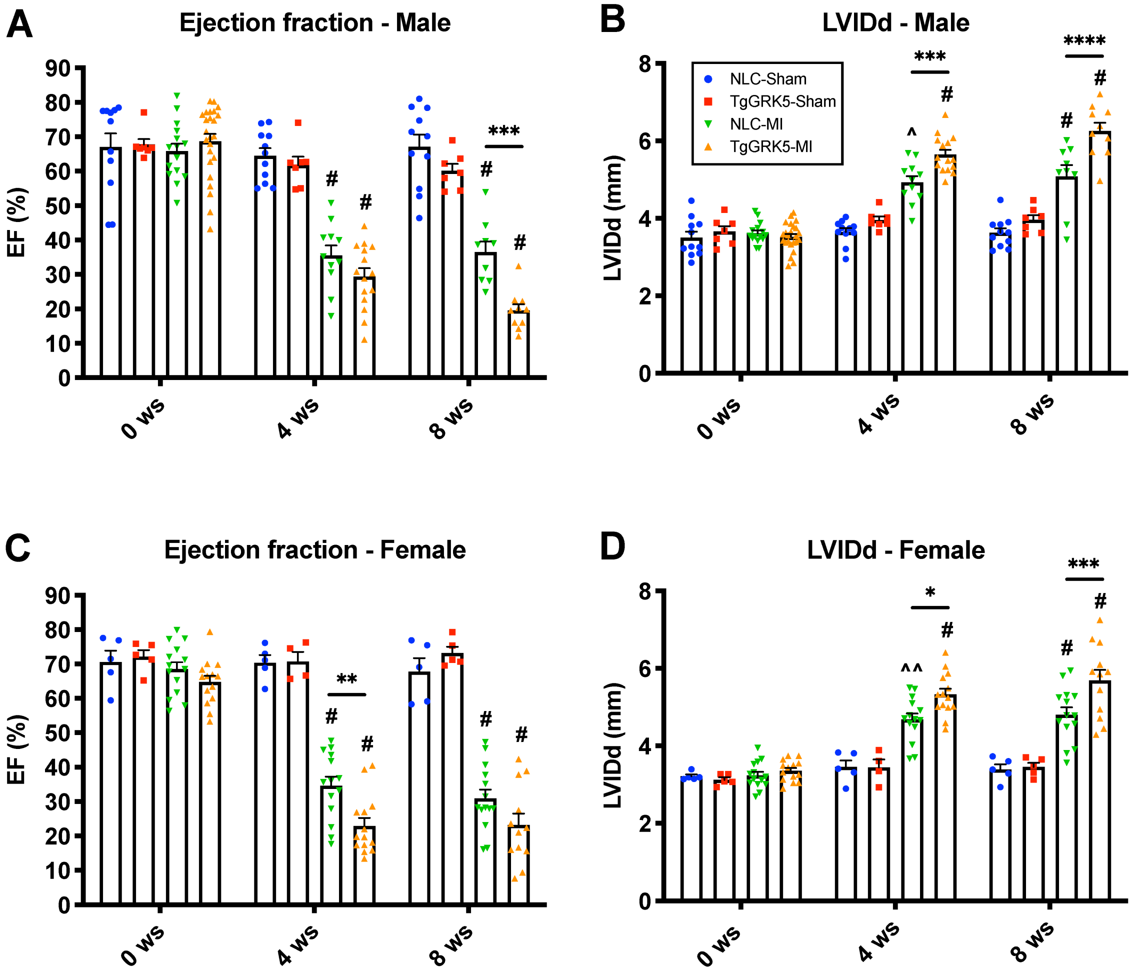

Suppl. Figure 4

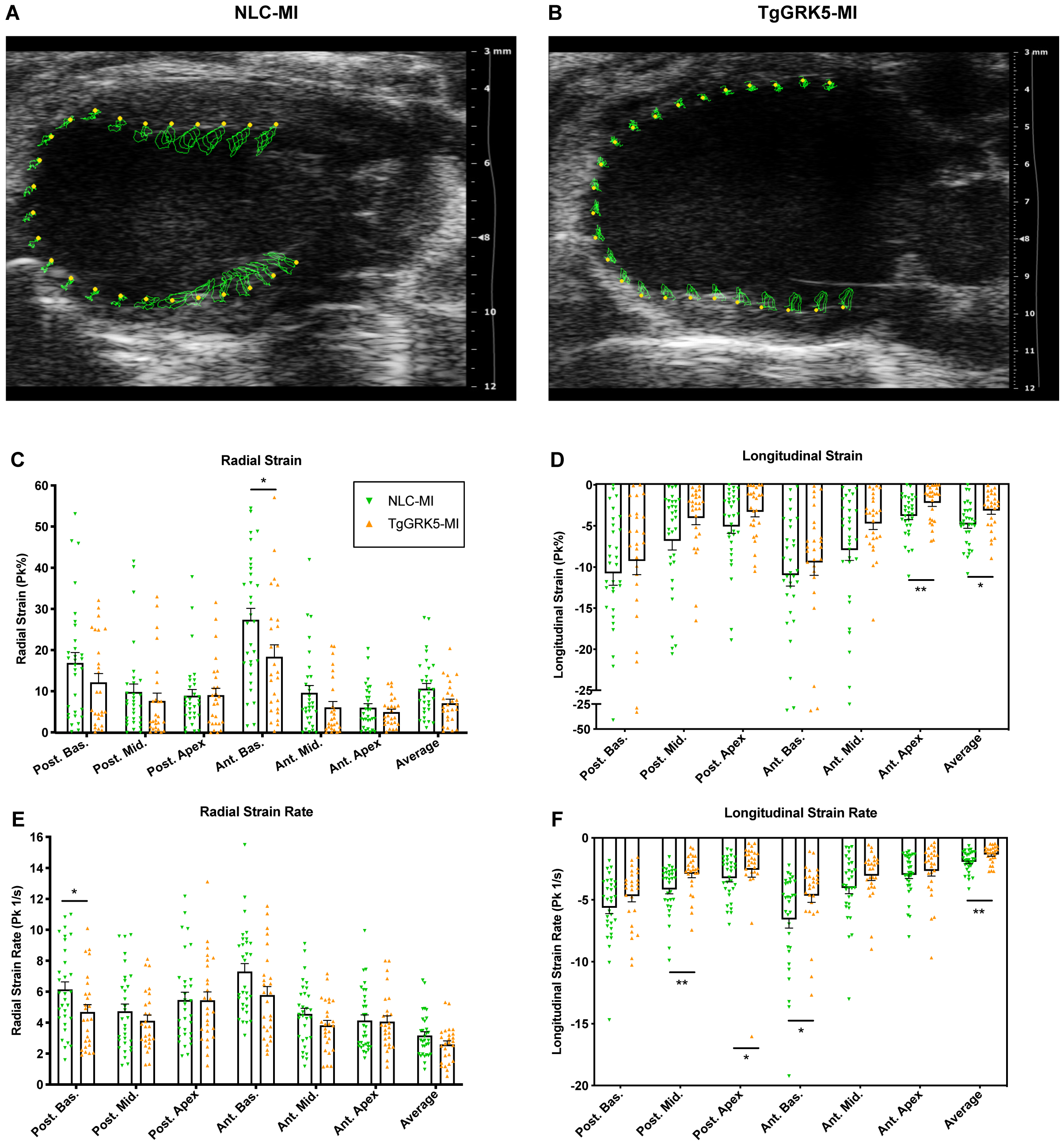

Suppl. Figure 5

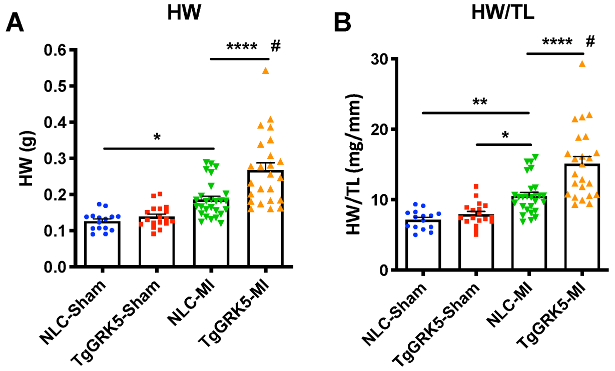

Suppl. Figure 6

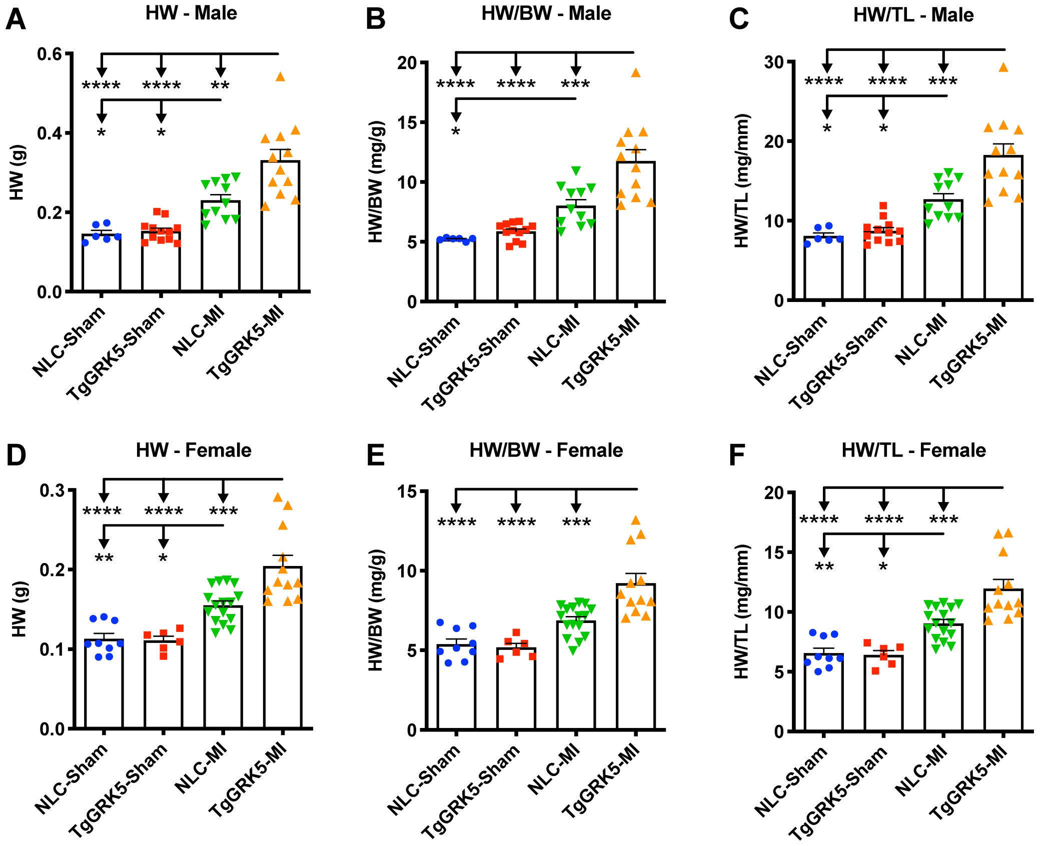

Suppl. Figure 7

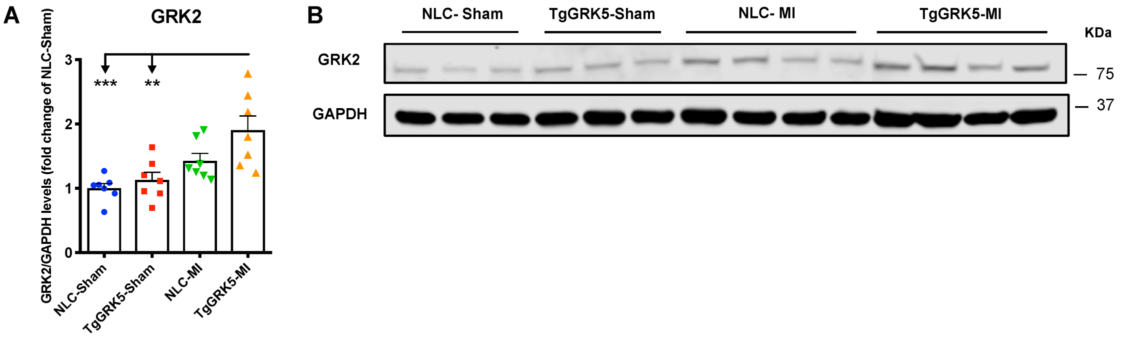

Suppl. Figure 8

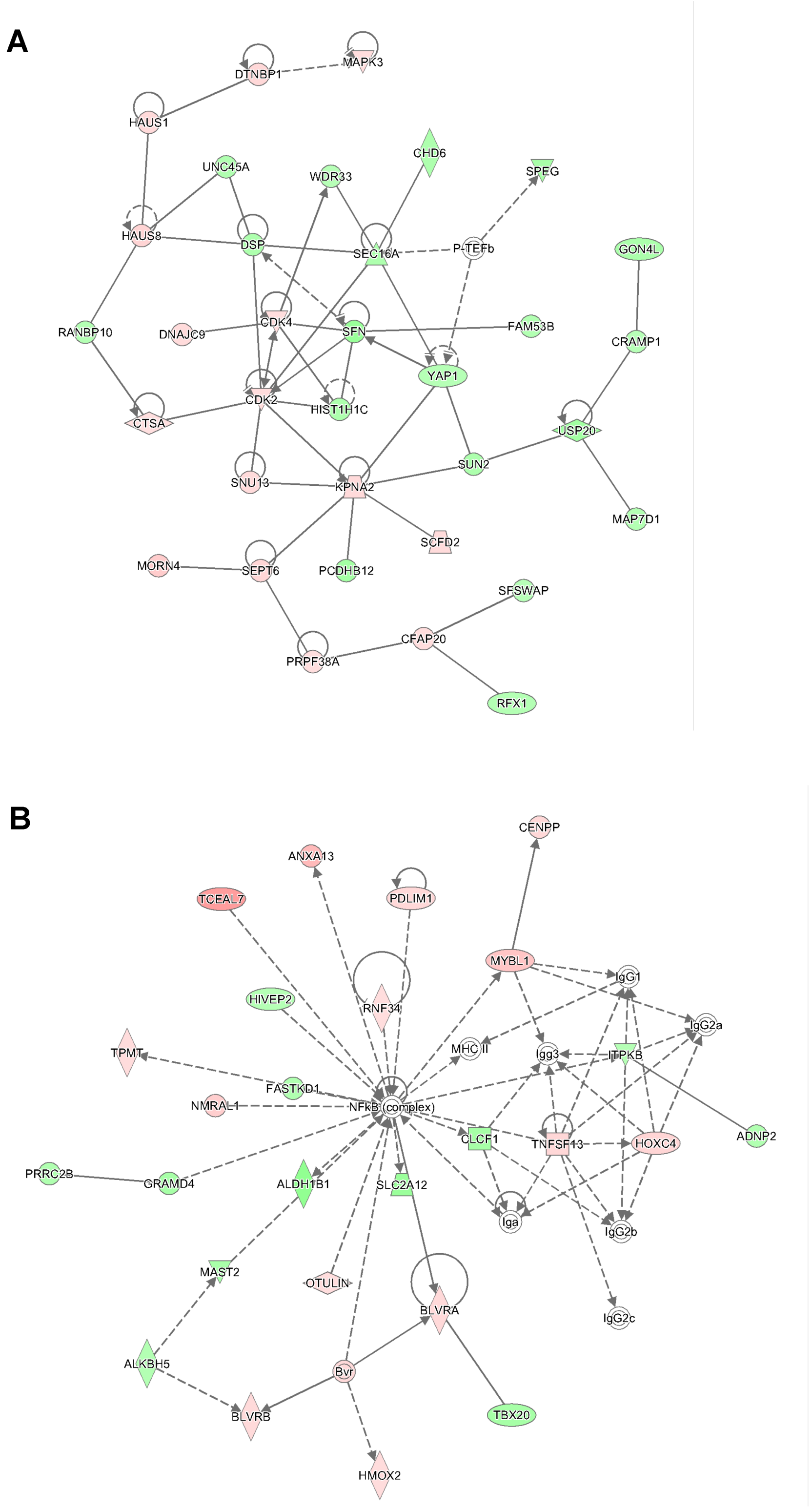

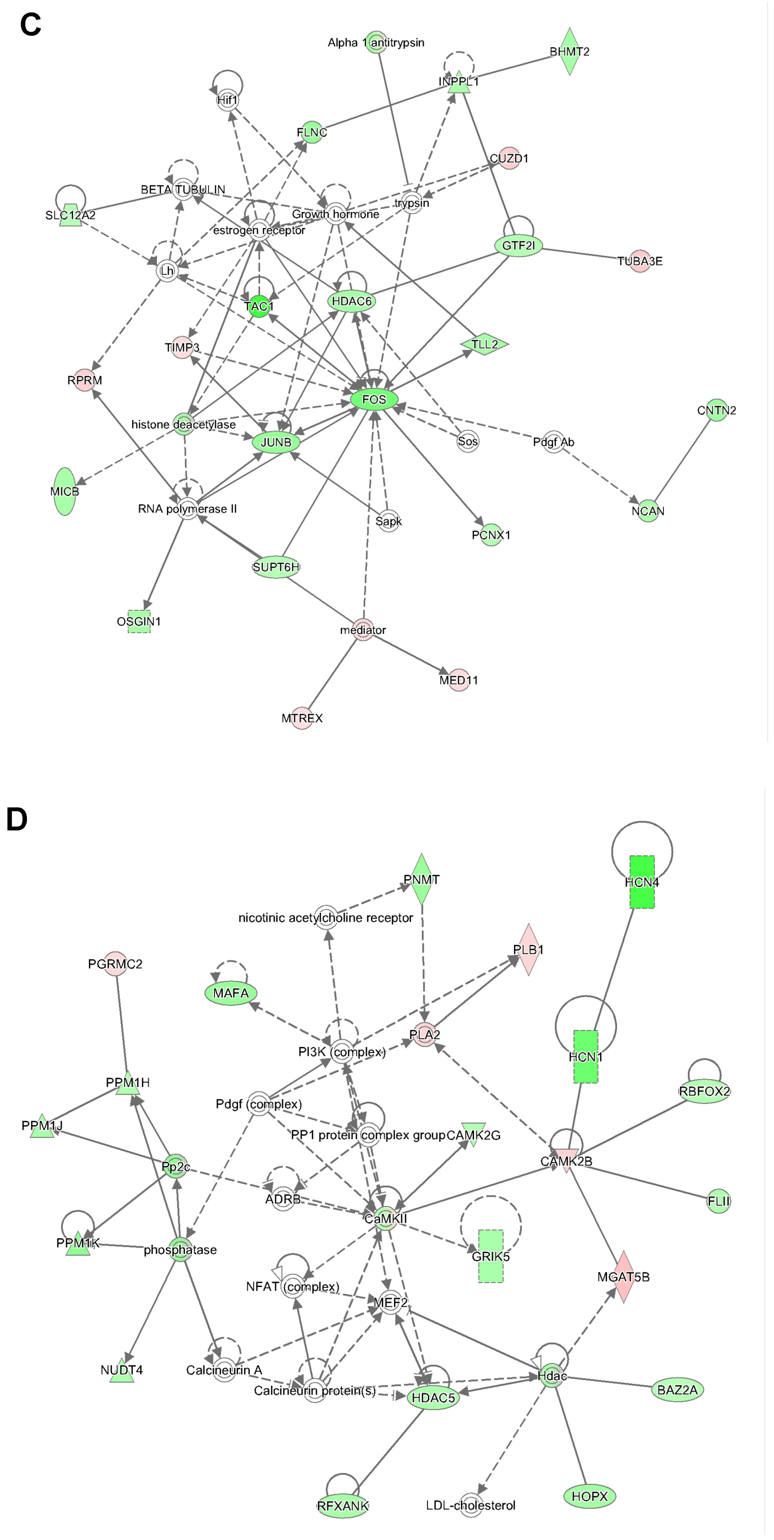
C
Alpha 1 antitrypsin
BHMT2
INPPL1
Hif1
FLNC
CUZD1
BETA TUBULIN
SLC12A2
Growth hormone
trypsin
estrogen receptor
GTF2I
TUBA3E
Lh
TAC1
HDAC6
TIMP3
TLL2
RPRM
FOS
CNTN2
histone deacetylase
JUNB
Sos
Pdgf Ab
MICB
RNA polymerase II
Sapk
NCAN
PCNX1
SUPT6H
OSGIN1
mediator
MED11
MTREX
D
PNMT
HCN4
nicotinic acetylcholine receptor
PLB1
PGRMC2
MAFA
PLA2
PI3K (complex)
HCN1
PPM1H
Pdgf (complex)
RBFOX2
PPM1J
PP1 protein complex group
CAMK2G
CAMK2B
Pp2c
ADRB
FLII
CaMKII
PPM1K
phosphatase
GRIK5
MGAT5B
NFAT (complex)
MEF2
NUDT4
Calcineurin A
Hdac
BAZ2A
Calcineurin protein(s)
HDAC5
RFXANK
LDL-cholesterol
HOPX

E

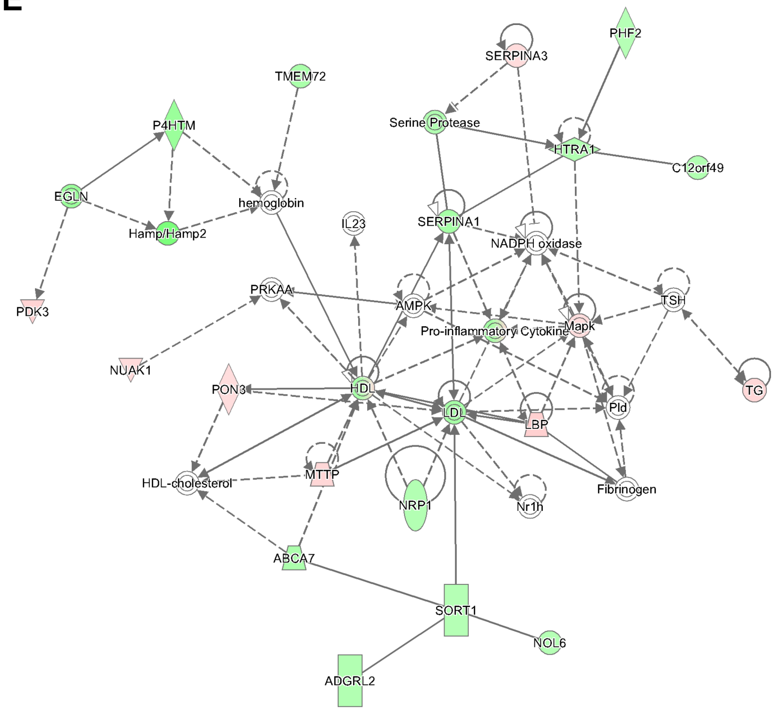
PHF2
SERPINA3
TMEM72
P4HTM
Serine Protease
HTRA1
C12orf49
EGLN
hemoglobin
Hamp/Hamp2
IL23
SERPINA1
NADPH oxidase
PRKAA
AMPK
TSH
PDK3
Pro-inflammatory Cytokine
Mapk
NUAK1
PON3
HDL
LDL
LBP
Pld
TG
MTTP
HDL-cholesterol
NRP1
Nr1h
Fibrinogen
ABCA7
SORT1
NOL6
ADGRL2

F

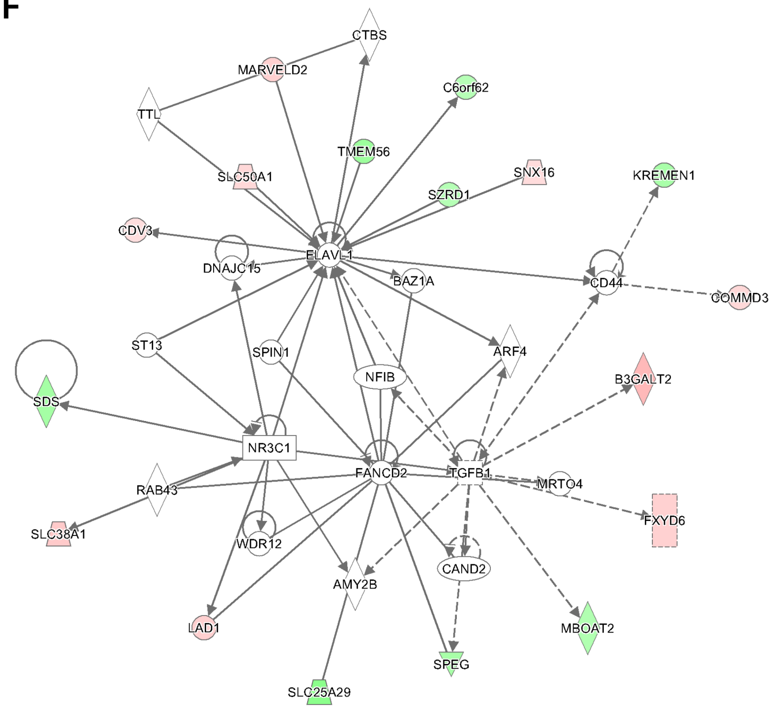
CTBS
MARVELD2
C6orf62
TTL
TMEM56
SLC50A1
SNX16
KREMEN1
SZRD1
CDV3
ELAVL1
DNAJC15
BAZ1A
CD44
COMMD3
ST13
SPIN1
NFIB
ARF4
B3GALT2
SDS
NR3C1
FANCD2
TGFB1
MRTO4
RAB43
FXYD6
SLC38A1
WDR12
CAND2
AMY2B
LAD1
MBOAT2
SPEG
SLC25A29

G

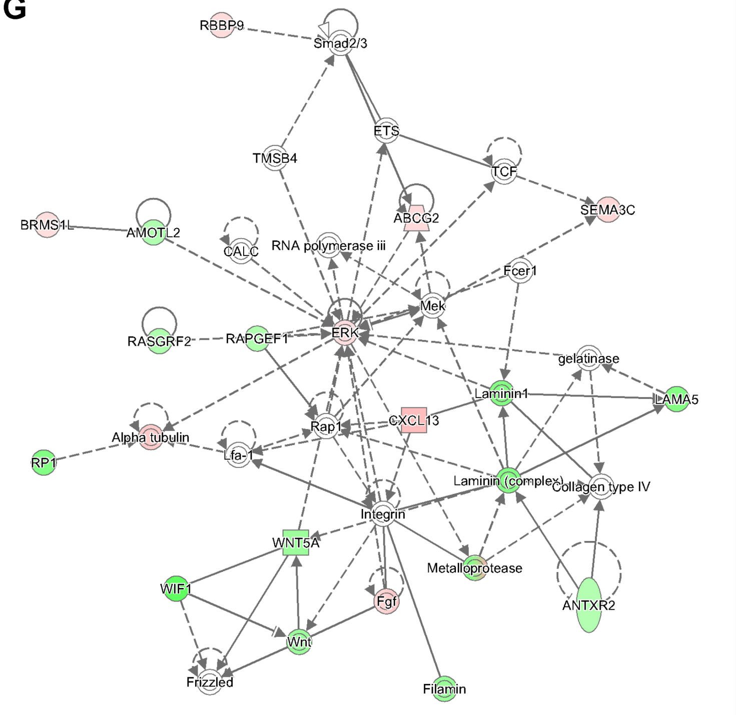
RBBP9
Smad2/3
ETS
TMSB4
TCF
SEMA3C
BRMS1L
AMOTL2
CALC
RNA polymerase iii
ABCG2
Fcer1
Mek
RASGRF2
RAPGEF1
ERK
gelatinase
Laminin1
LAMA5
CXCL13
Rap1
Alpha tubulin
Lfa-1
RP1
Laminin (complex)
Collagen type IV
Integrin
WNT5A
Metalloprotease
WIF1
Fgf
ANTXR2
Wnt
Frizzled
Filamin

H

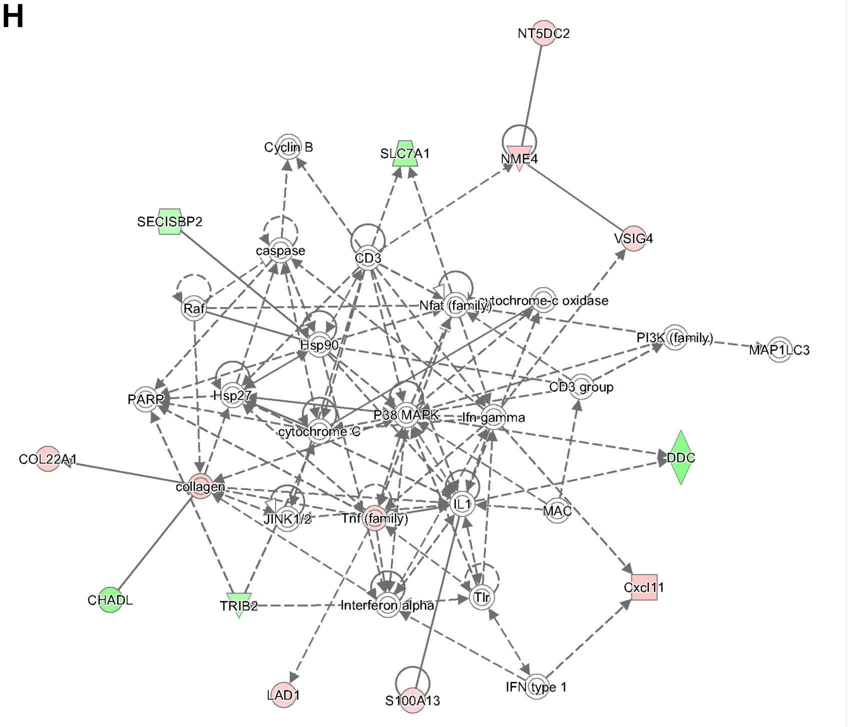
NT5DC2
Cyclin B
SLC7A1
NME4
SECISBP2
VSIG4
caspase
CD3
Raf
Nfat (family)
cytochrome-c oxidase
Hsp90
PI3K (family)
MAP1LC3
CD3 group
PARP
Hsp27
P38 MAPK
Ifn gamma
cytochrome C
COL22A1
DDC
collagen
JINK1/2
Tnf (family)
IL1
MAC
CHADL
TRIB2
Interferon alpha
Tlr
Cxcl11
LAD1
S100A13
IFN type 1

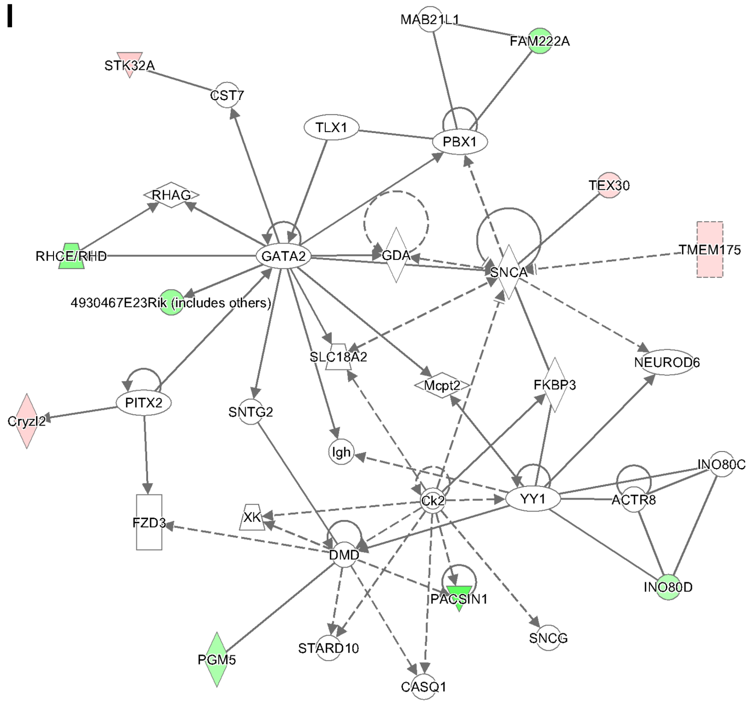
I
MAB21L1
FAM222A
STK32A
CST7
TLX1
PBX1
TEX30
RHAG
TMEM175
RHCE/RHD
GATA2
GDA
SNCA
4930467E23Rik (includes others)
SLC18A2
NEUROD6
Mcpt2
FKBP3
PITX2
Cryzl2
SNTG2
Igh
INO80C
FZD3
XK
Ck2
YY1
ACTR8
DMD
PACSIN1
INO80D
SNCG
PGM5
STARD10
CASQ1

Suppl. Figure 9

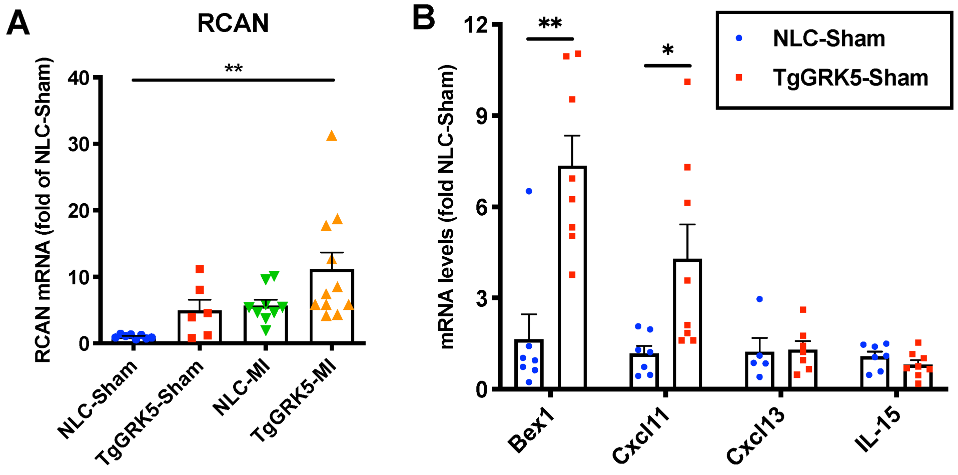

Suppl. Figure 10

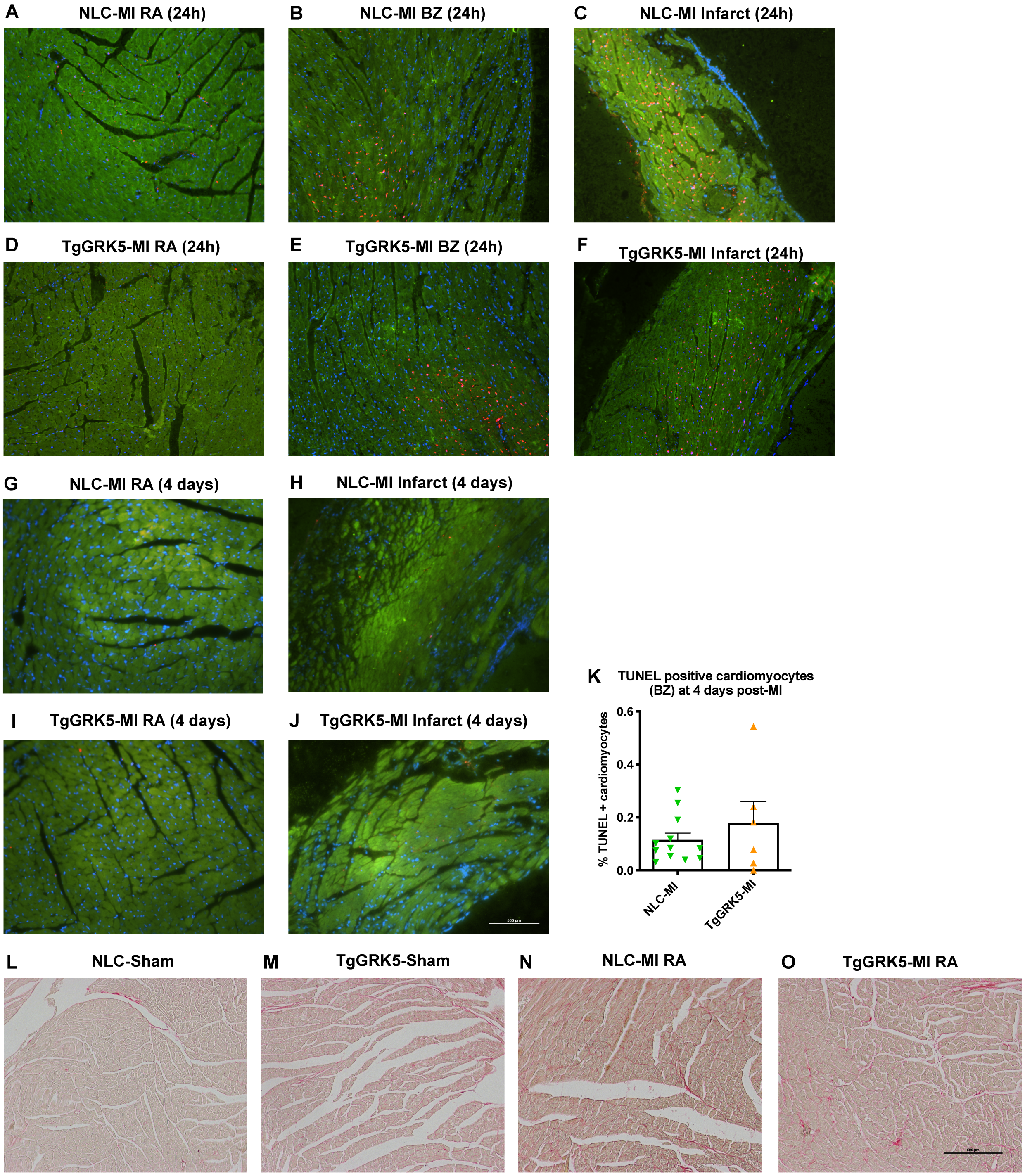

Suppl. Figure 11

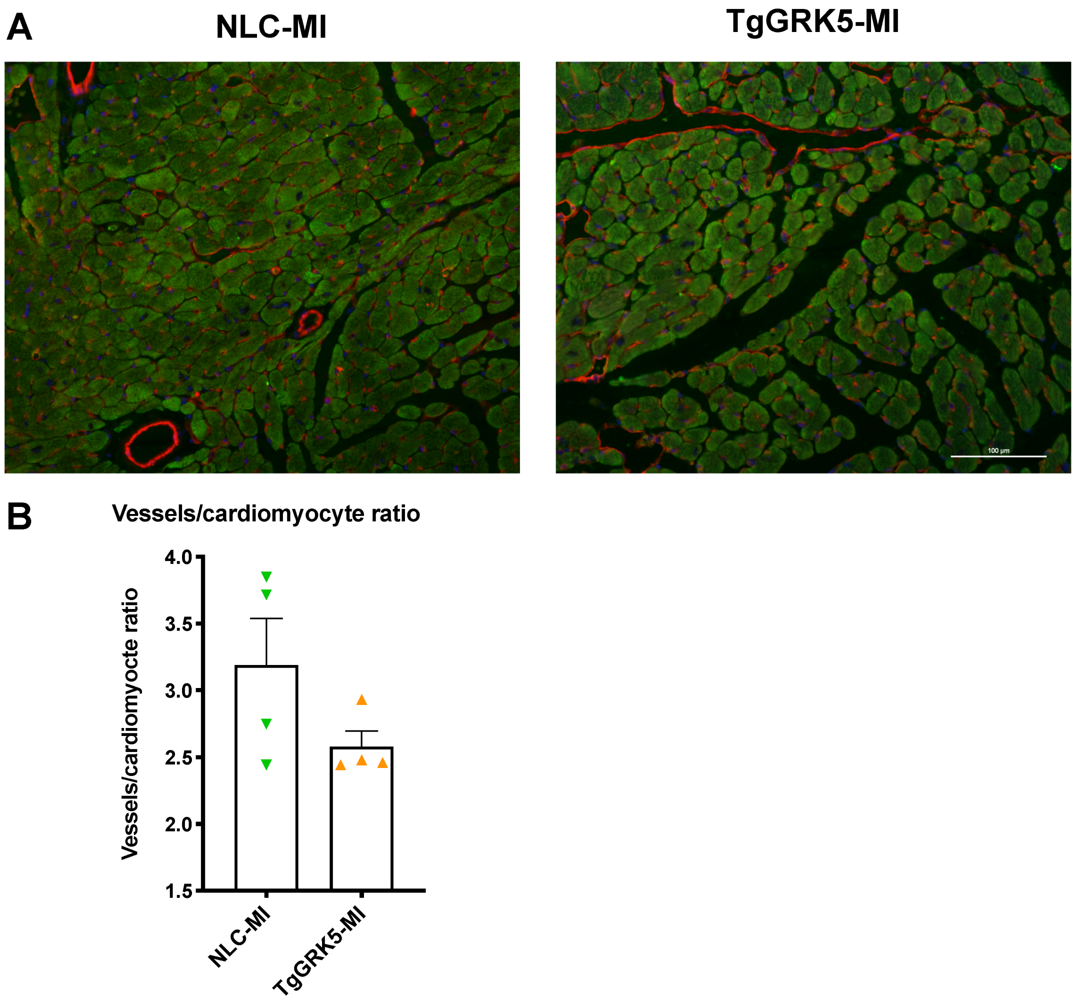

Suppl. Figure 12

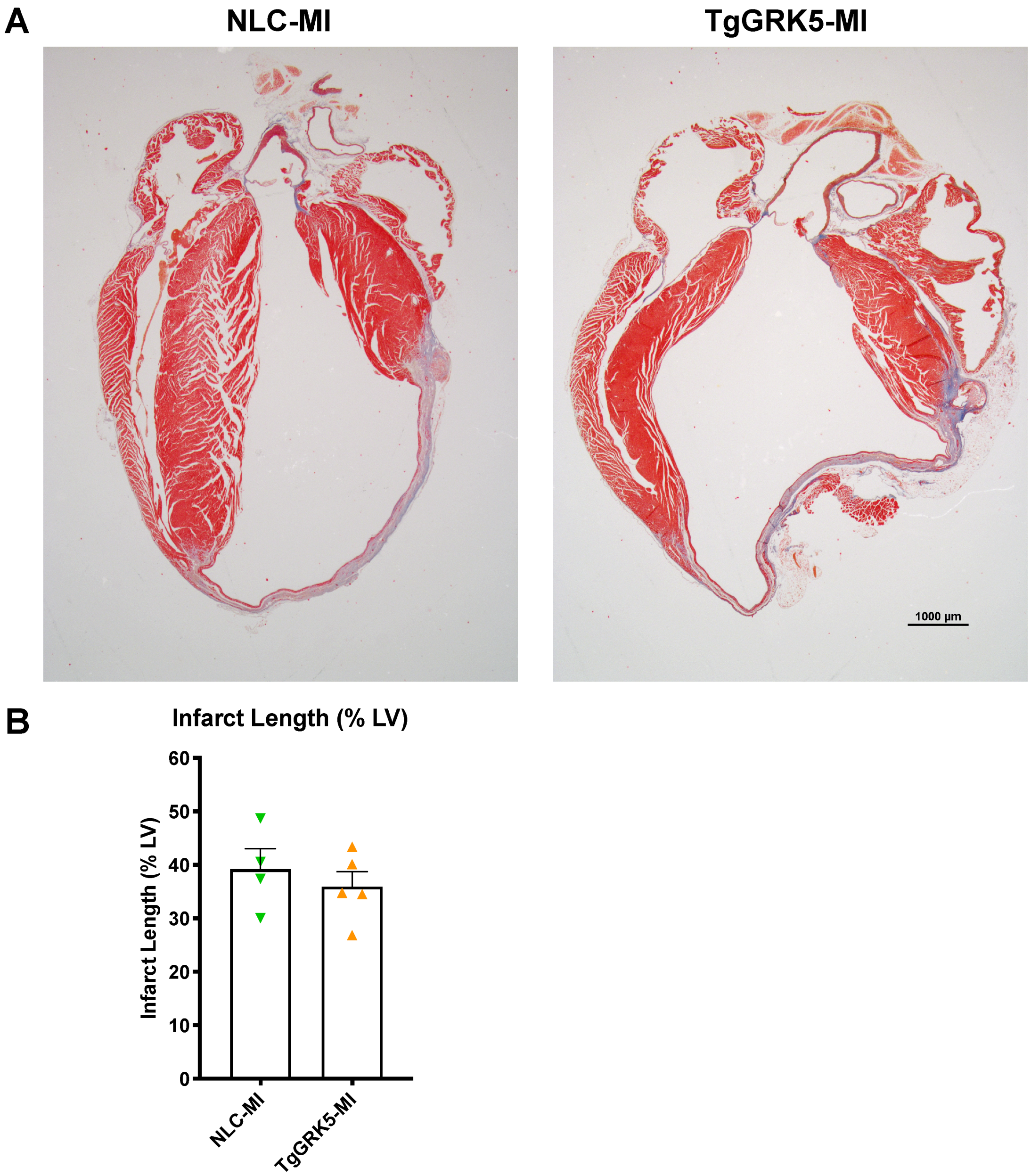

Suppl. Figure 13

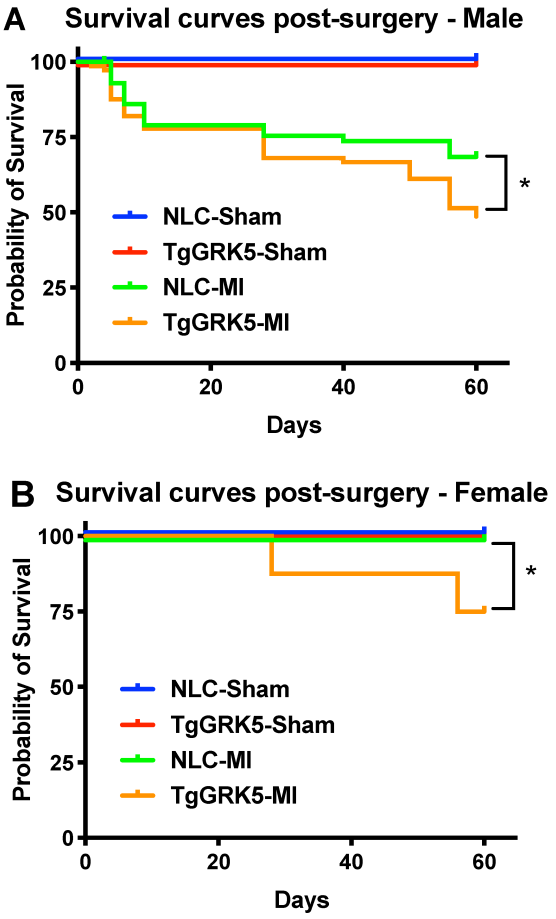

Suppl. Figure 14

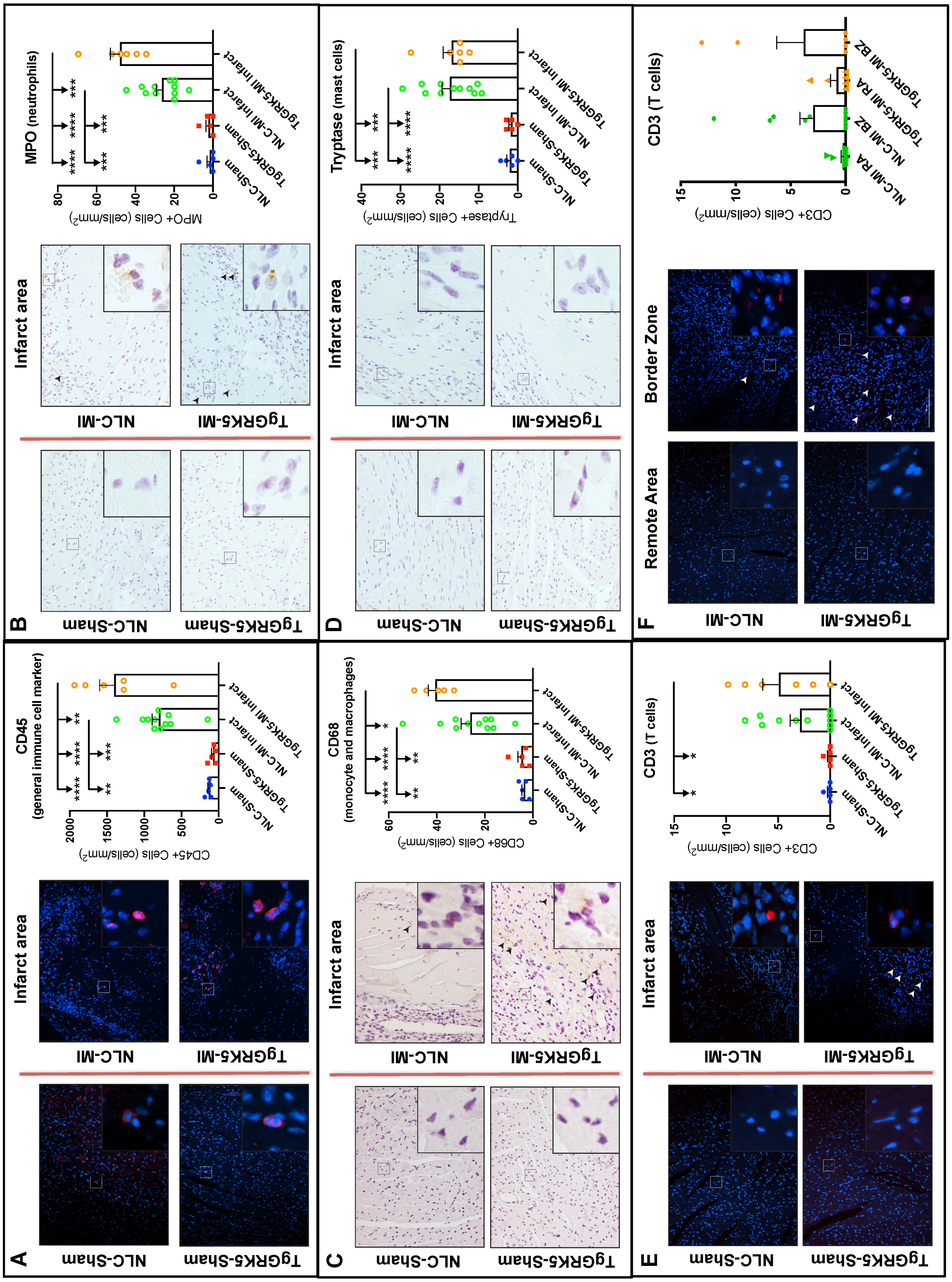

Suppl. Figure 15

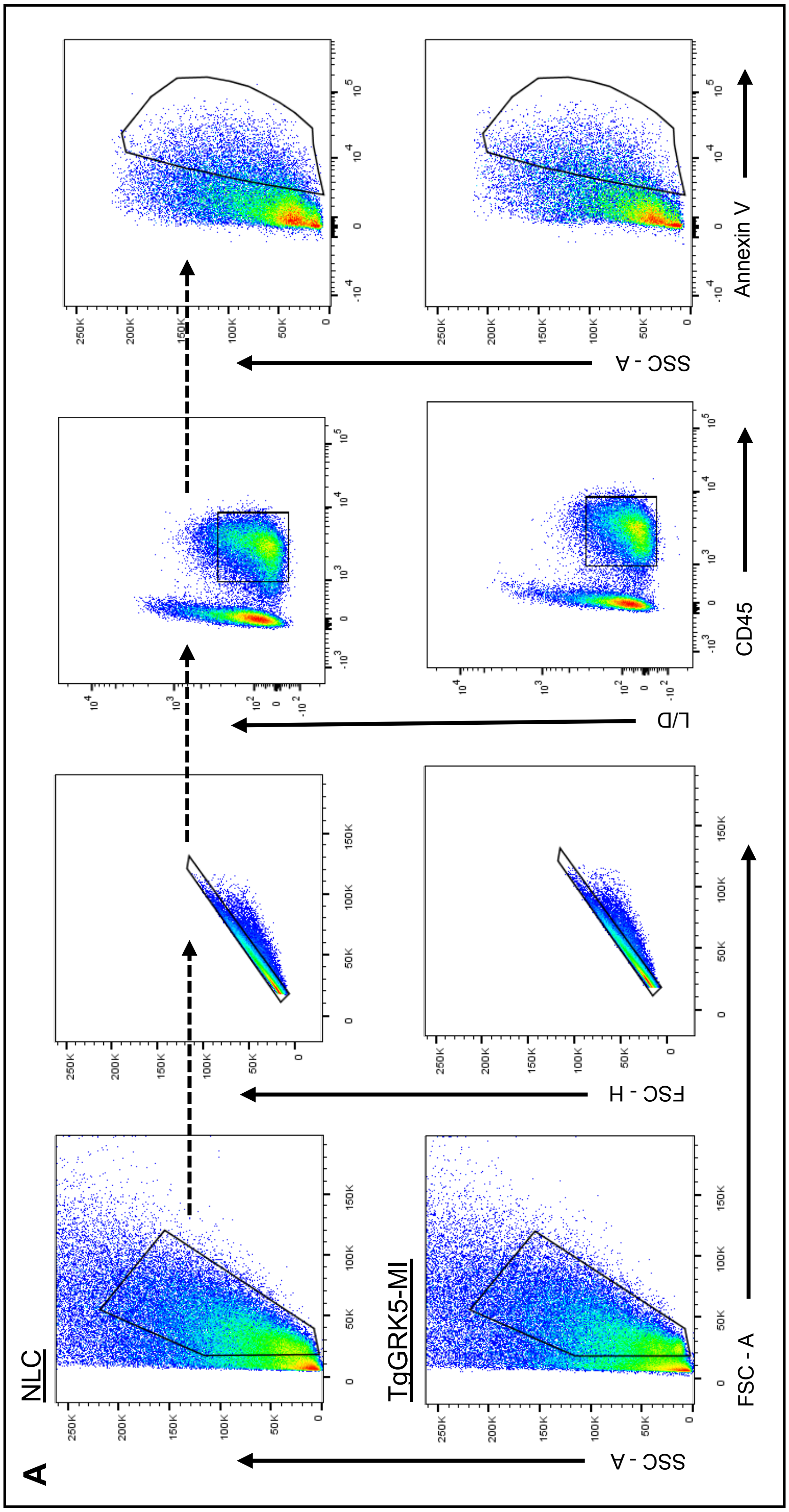

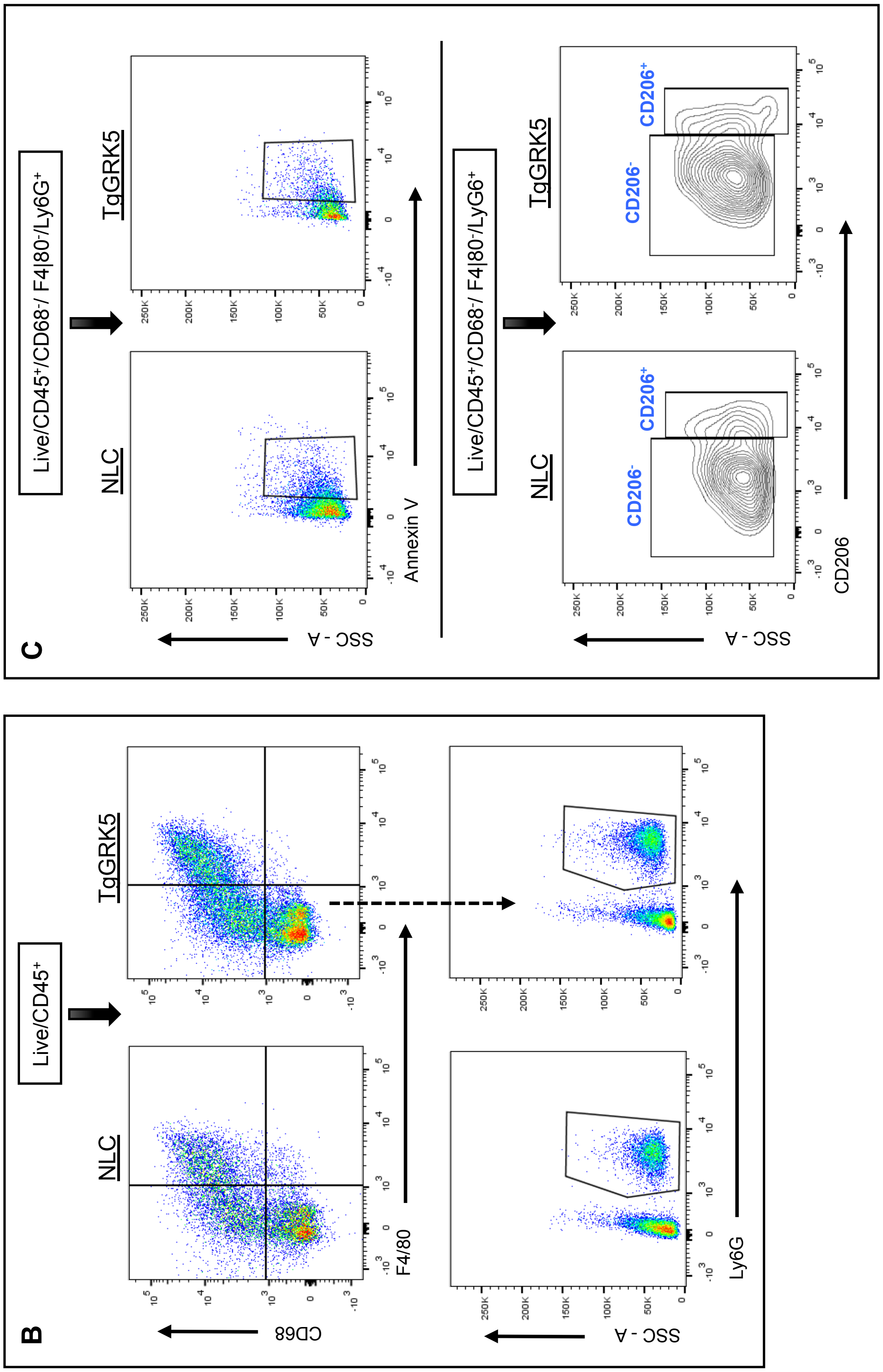
C
Live/CD45⁺/CD68⁻/ F4|80⁻/Ly6G⁺
NLC
TgGRK5
SSC - A
Annexin V
Live/CD45⁺/CD68⁻/ F4|80⁻/LyG6⁺
CD206⁻
CD206⁺
CD206
B
Live/CD45⁺
CD68
F4/80
Ly6G

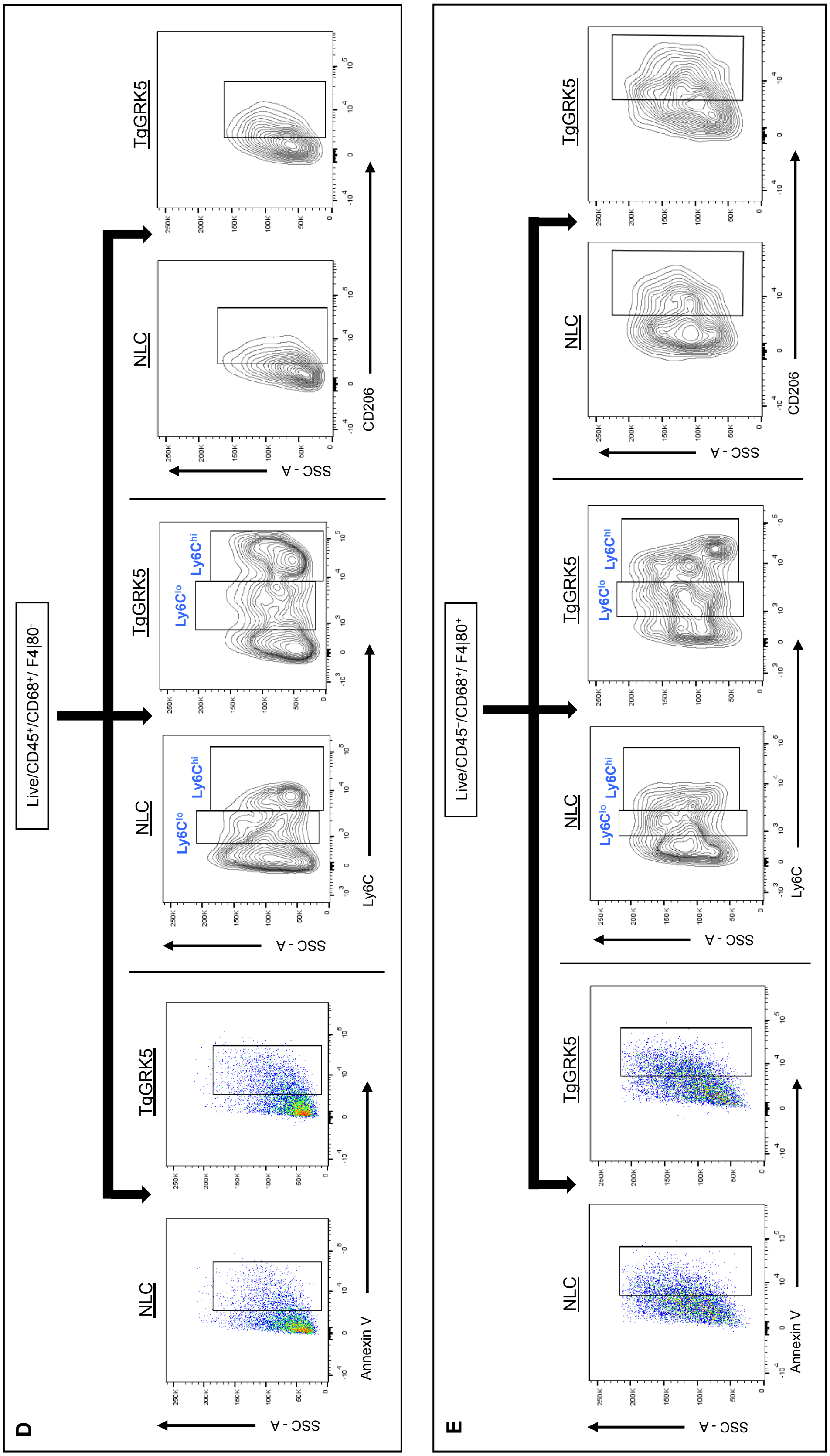
D
Live/CD45+/CD68+/ F4|80-
NLC
TgGRK5
SSC - A
Annexin V
Ly6Clo
Ly6Chi
Ly6C
CD206
E
Live/CD45+/CD68+/ F4|80+
NLC
TgGRK5
SSC - A
Annexin V
Ly6Clo
Ly6Chi
Ly6C
CD206

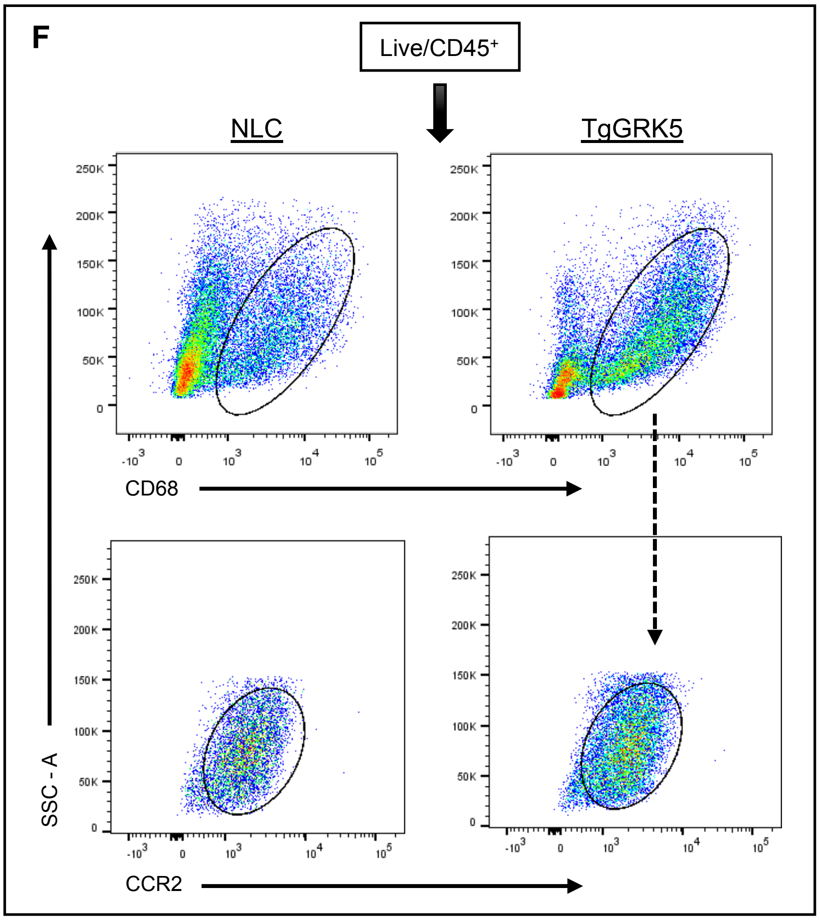
F
Live/CD45+
NLC
TgGRK5
CD68
SSC - A
CCR2

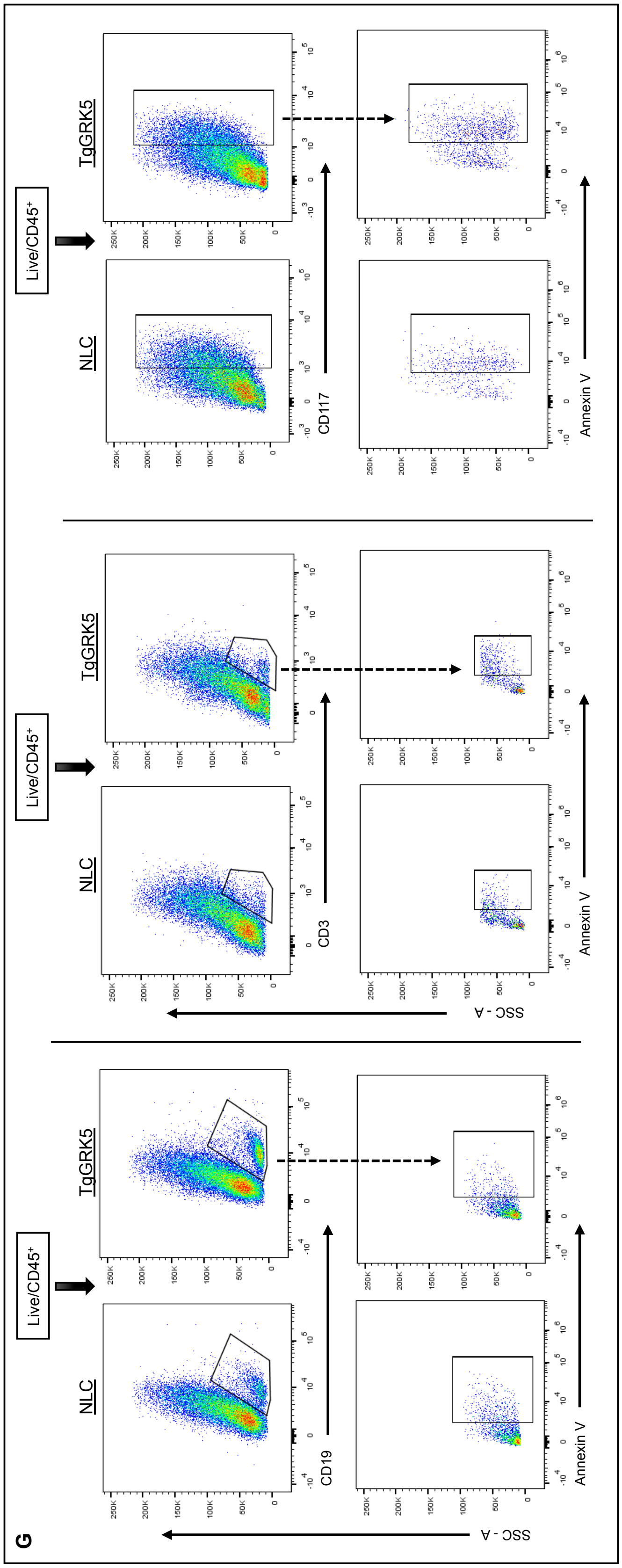
G
NLC
TgGRK5
Live/CD45+
CD19
CD3
CD117
Annexin V
SSC - A

Suppl. Figure 16

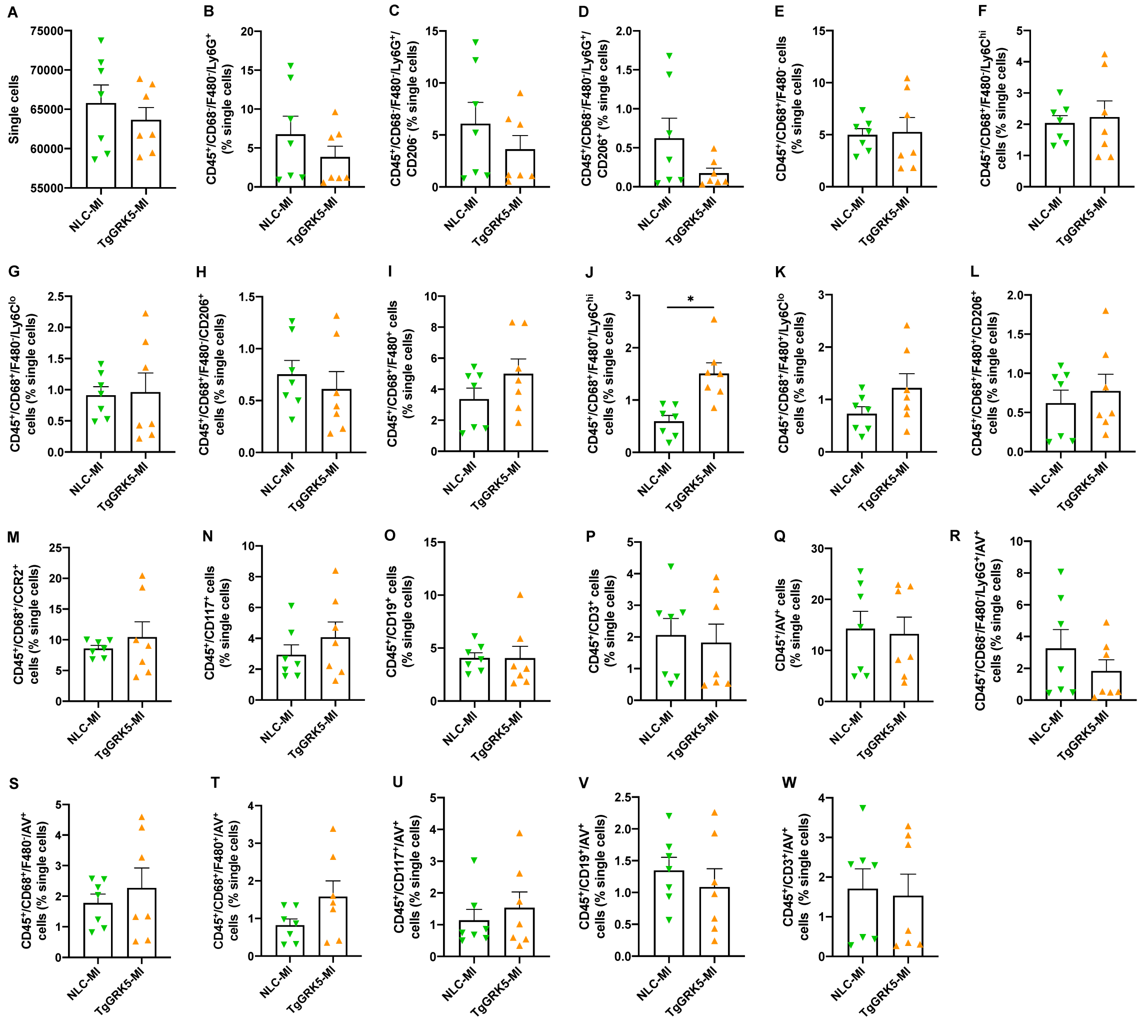

Suppl. Figure 17

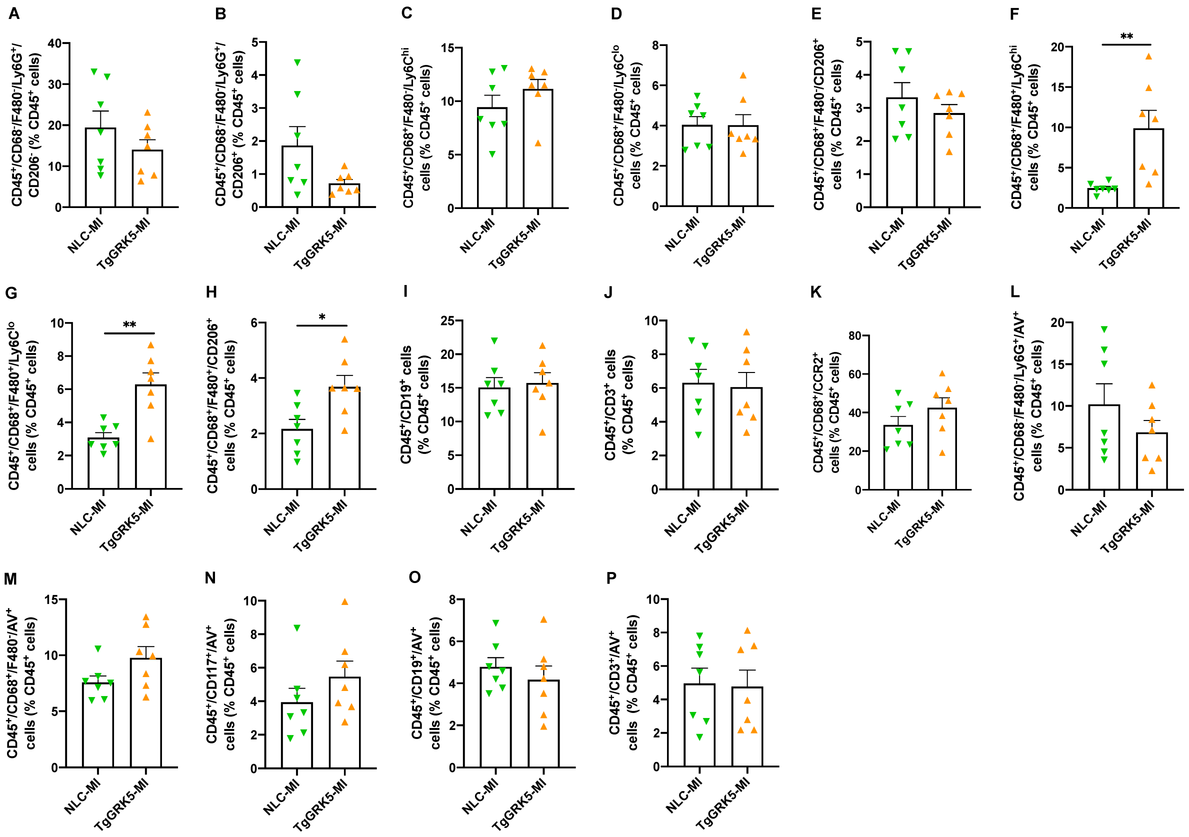

Suppl. Figure 18

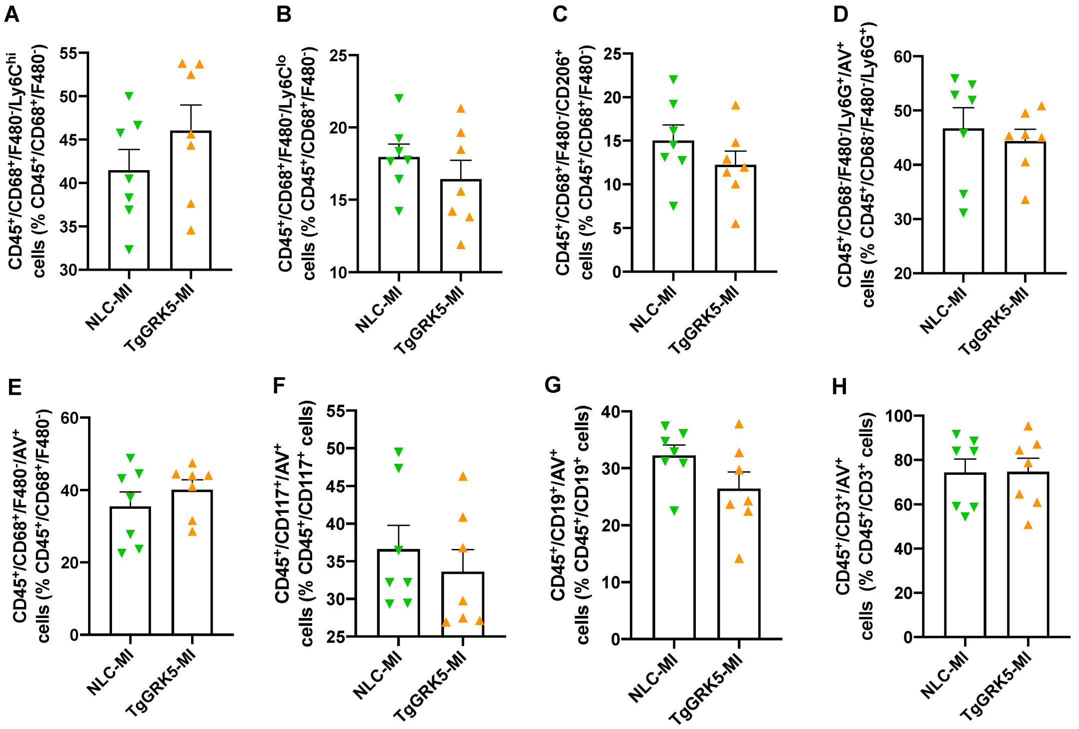

Suppl. Figure 19

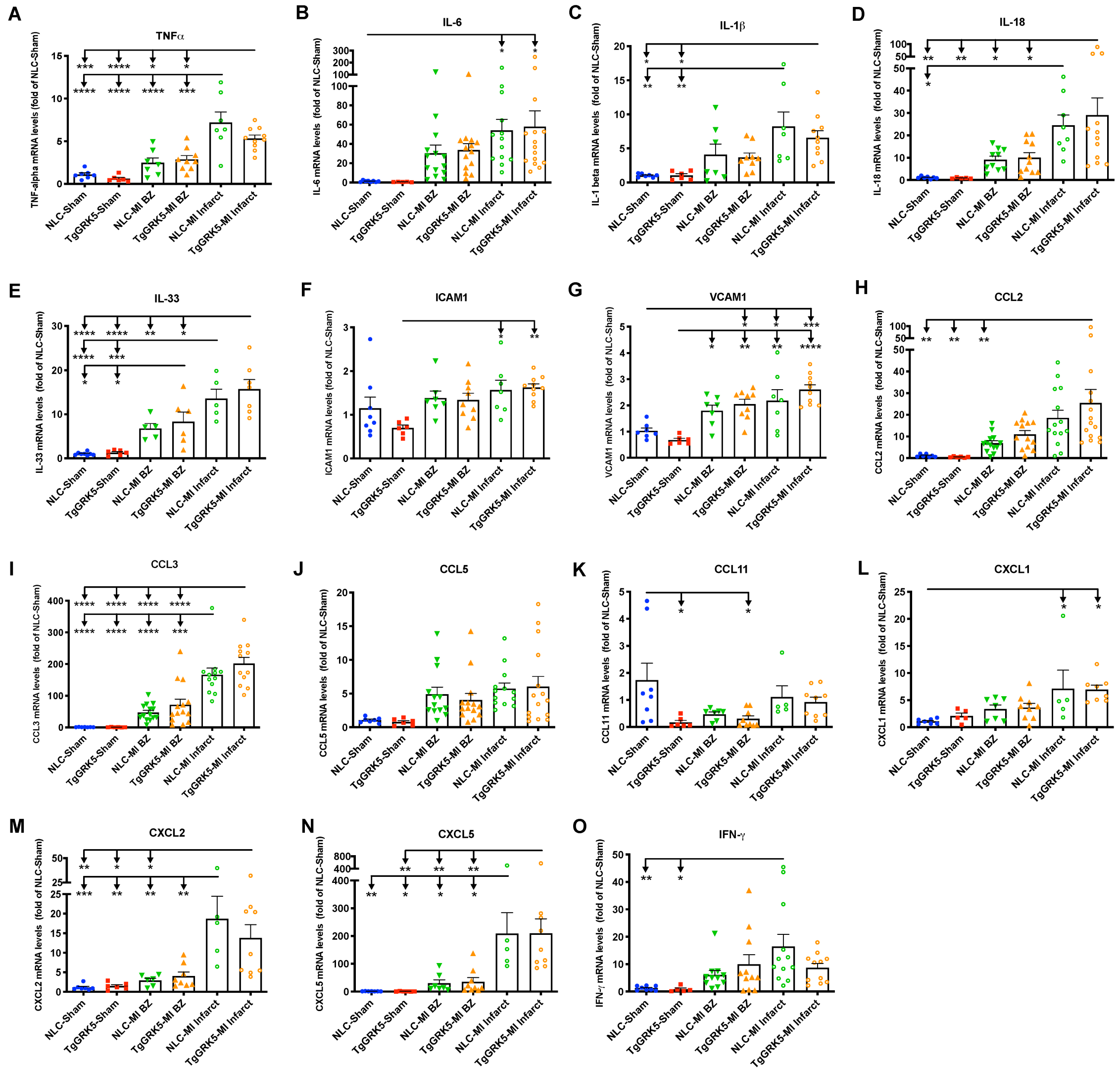

Suppl. Figure 20

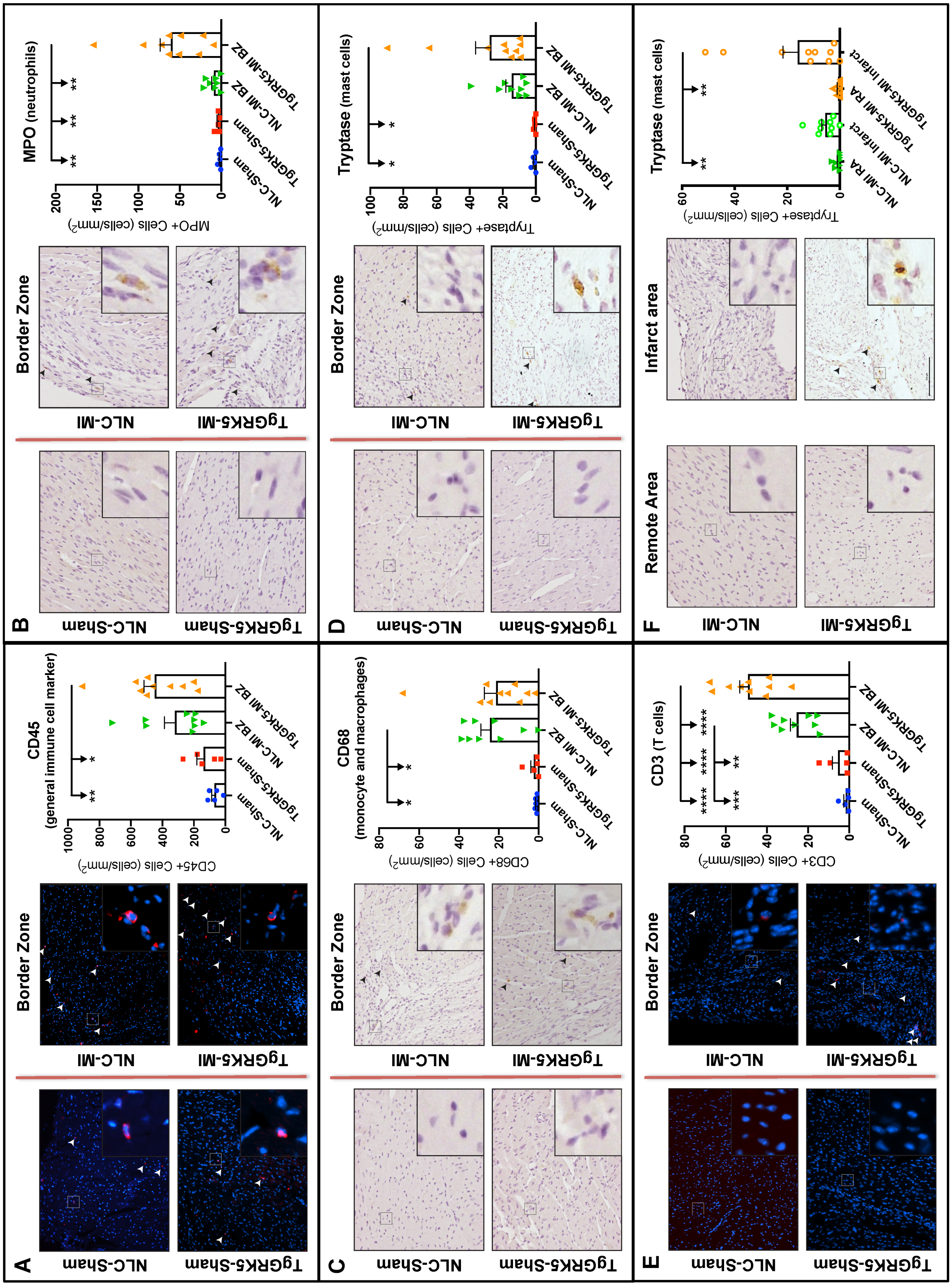

Suppl. Figure 21

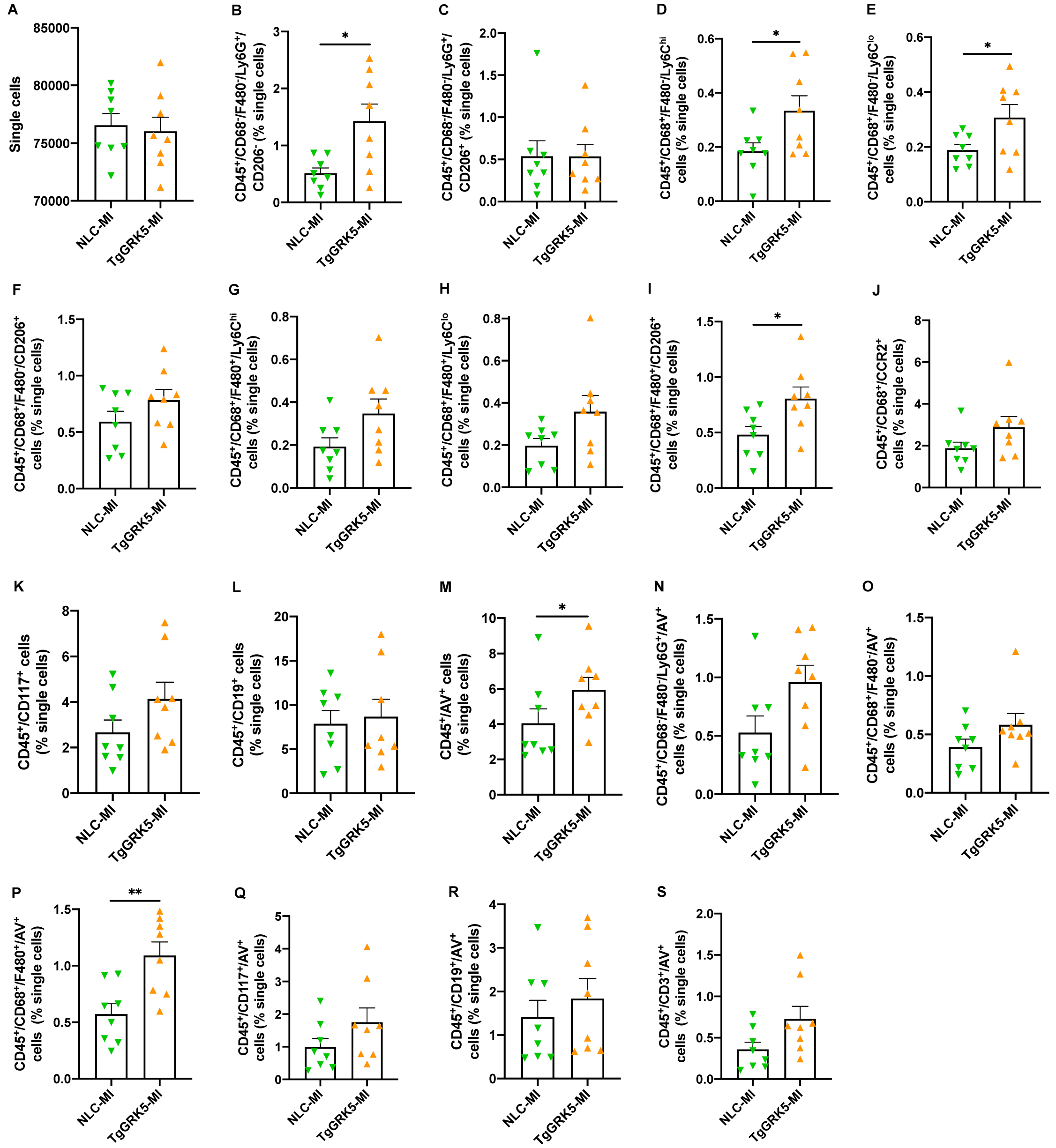

Suppl. Figure 22

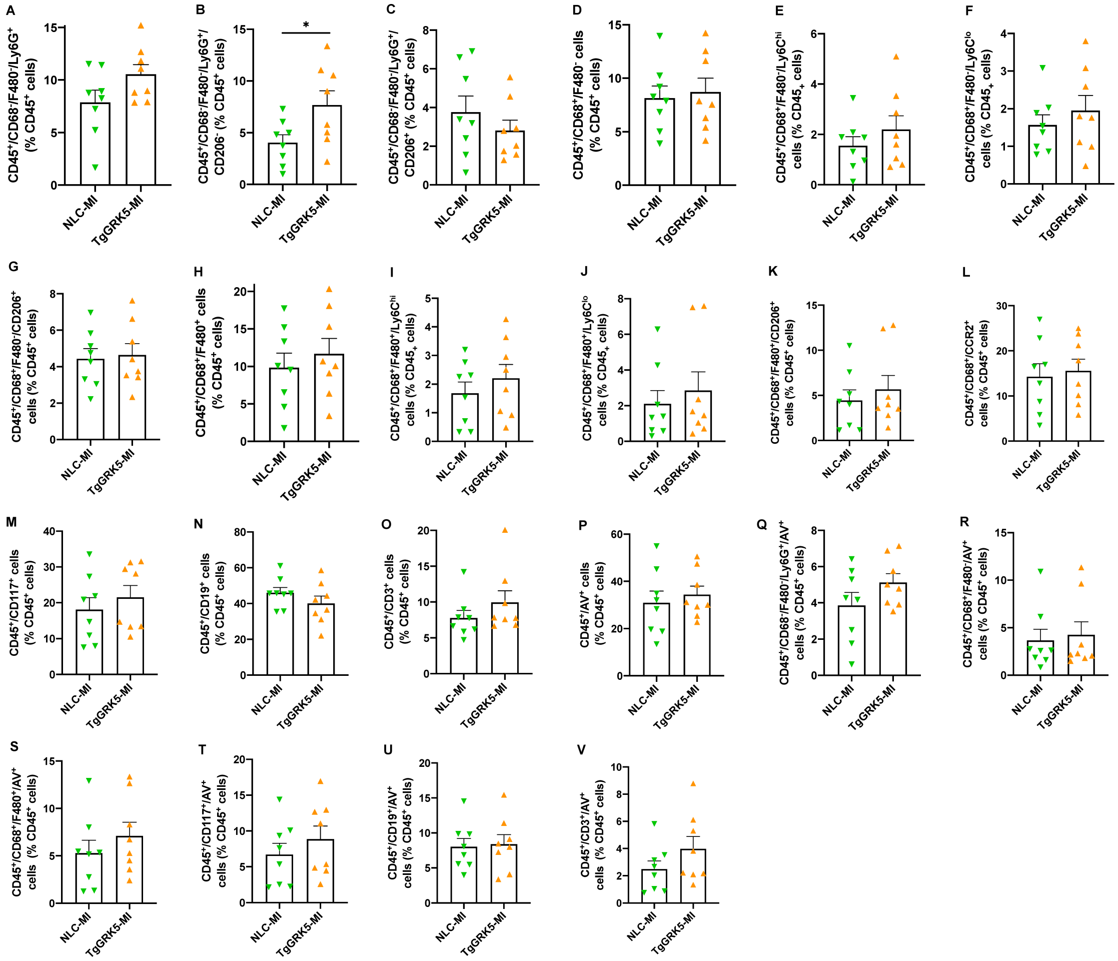

Suppl. Figure 23

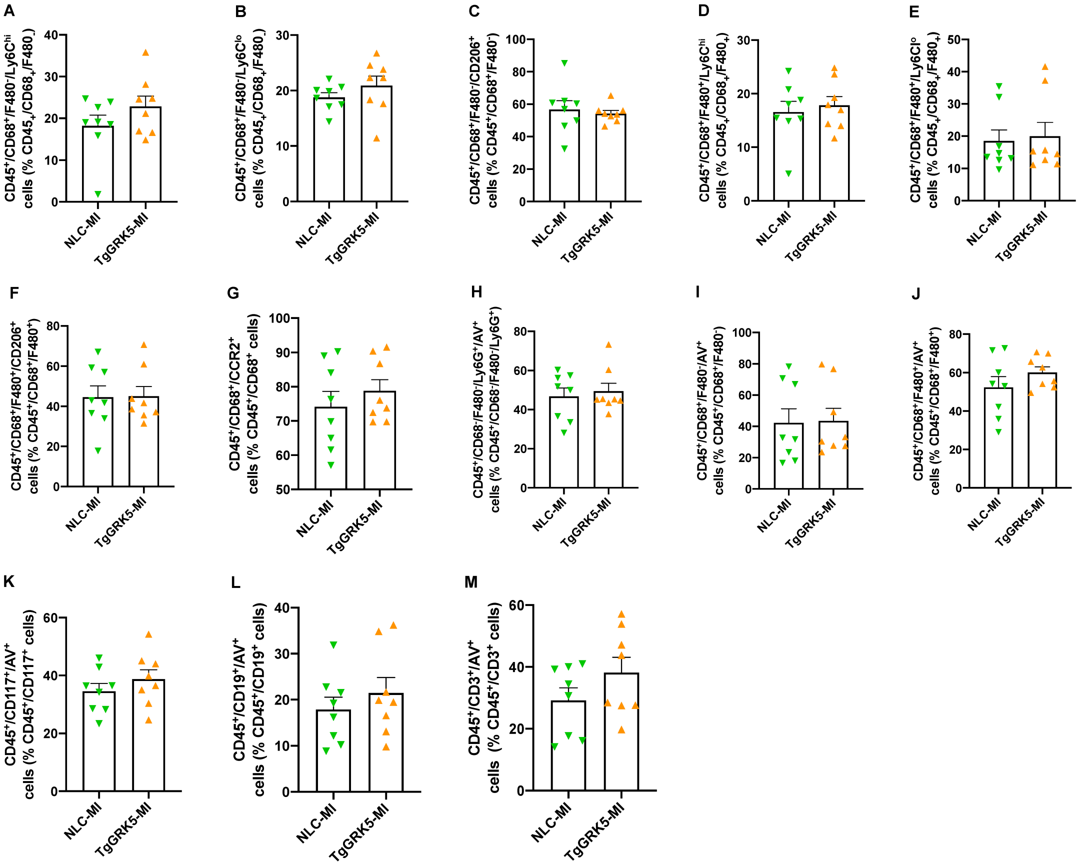

Suppl. Figure 24

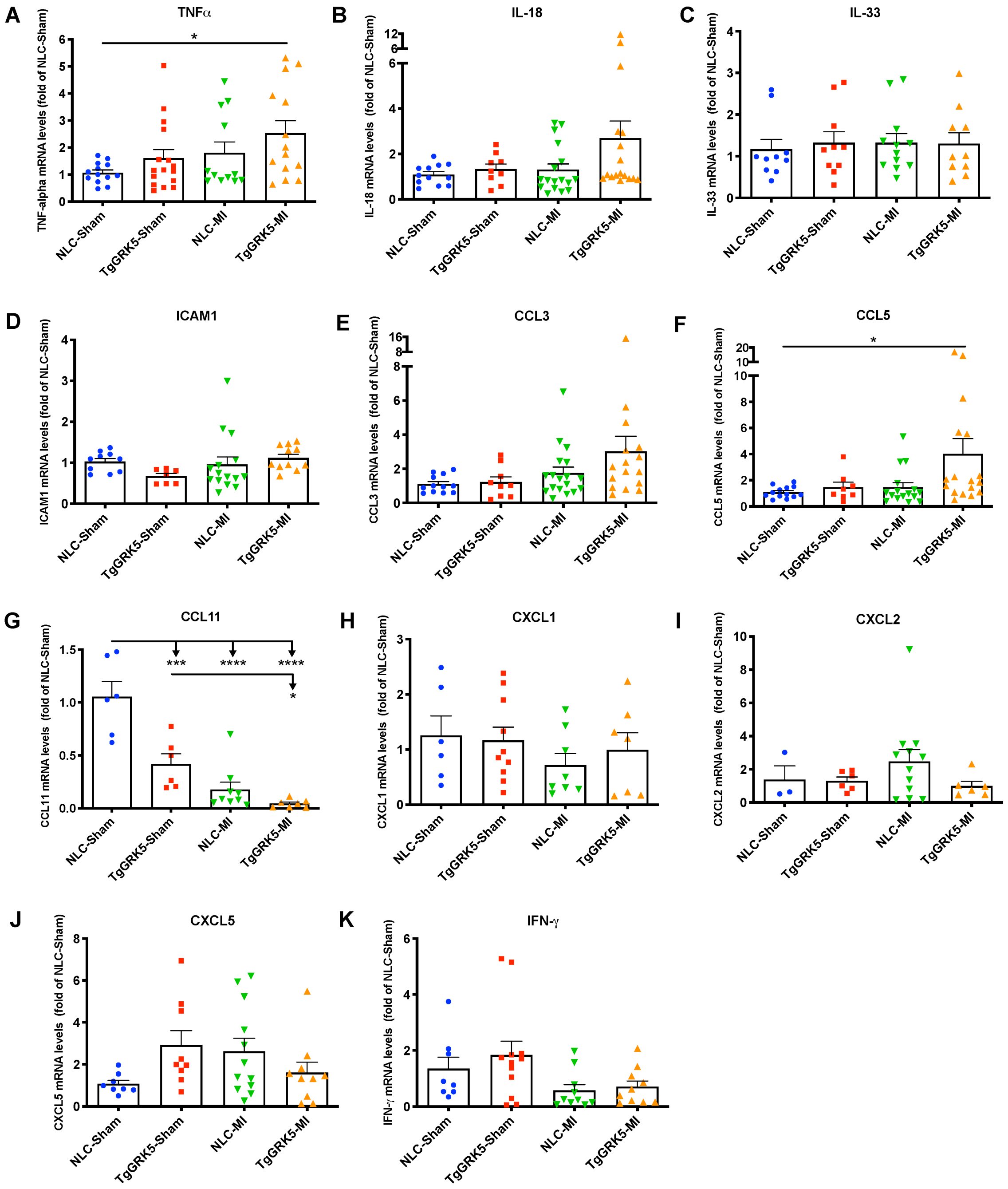

Suppl. Figure 25

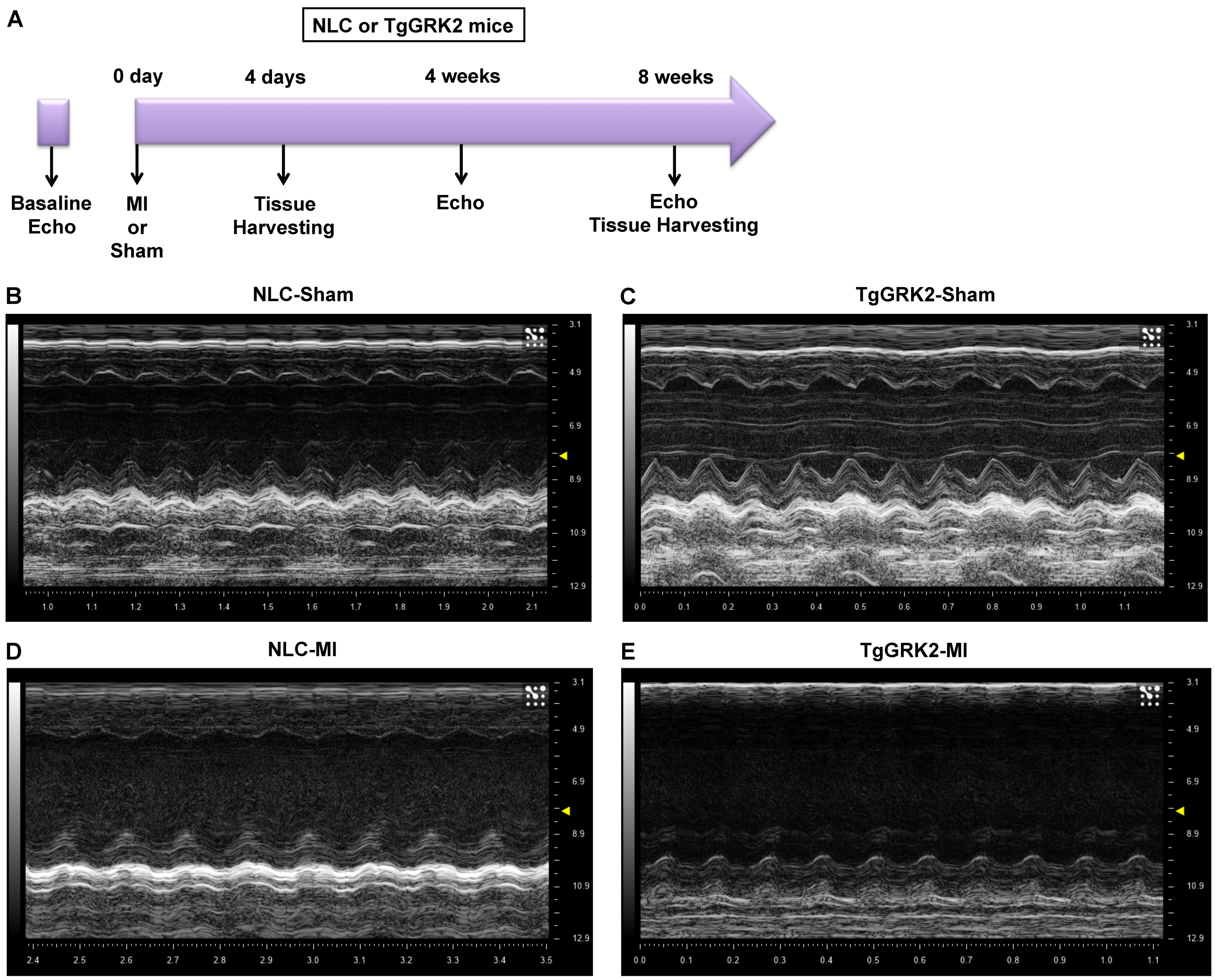

Suppl. Figure 26

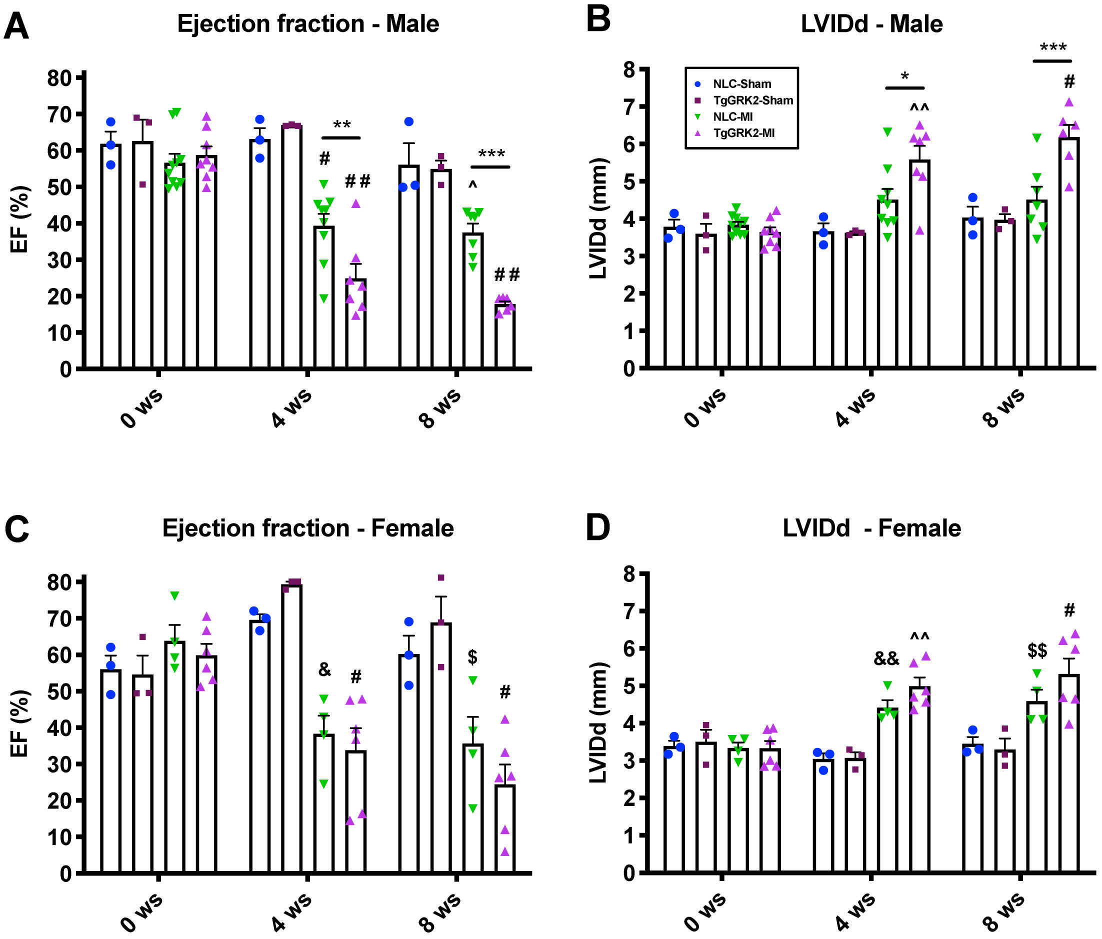

Suppl. Figure 27

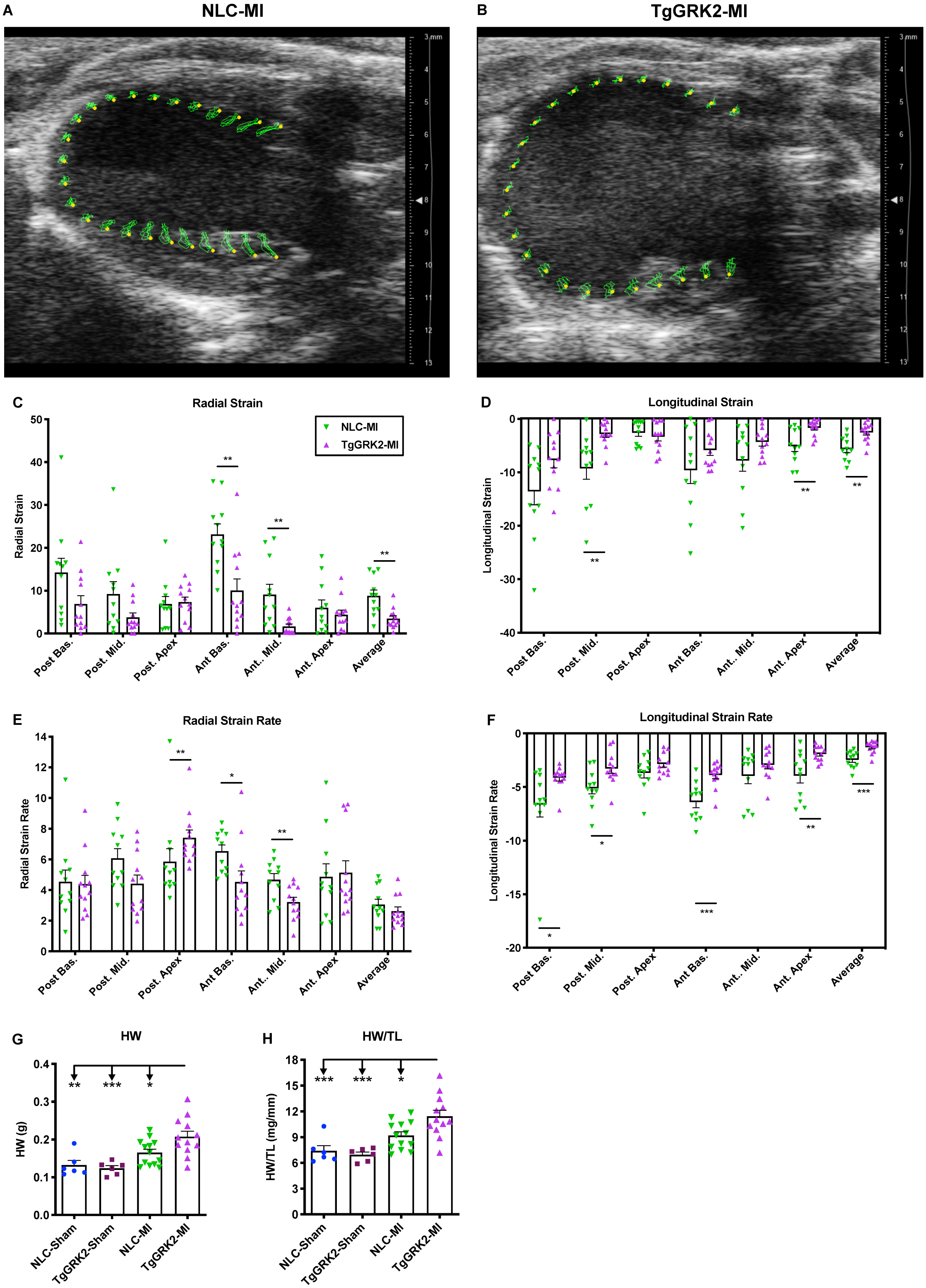

Suppl. Figure 28

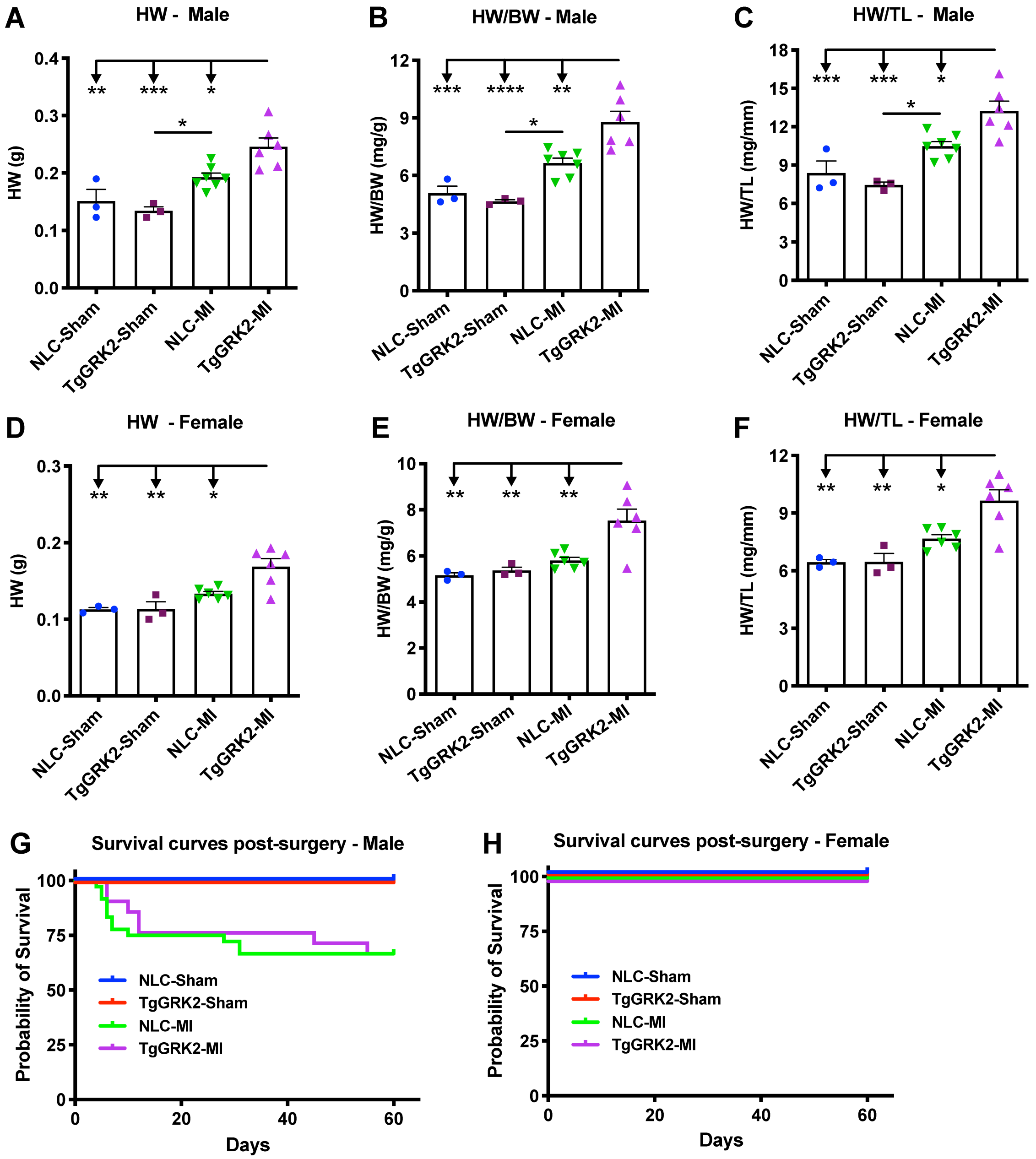

Suppl. Figure 29

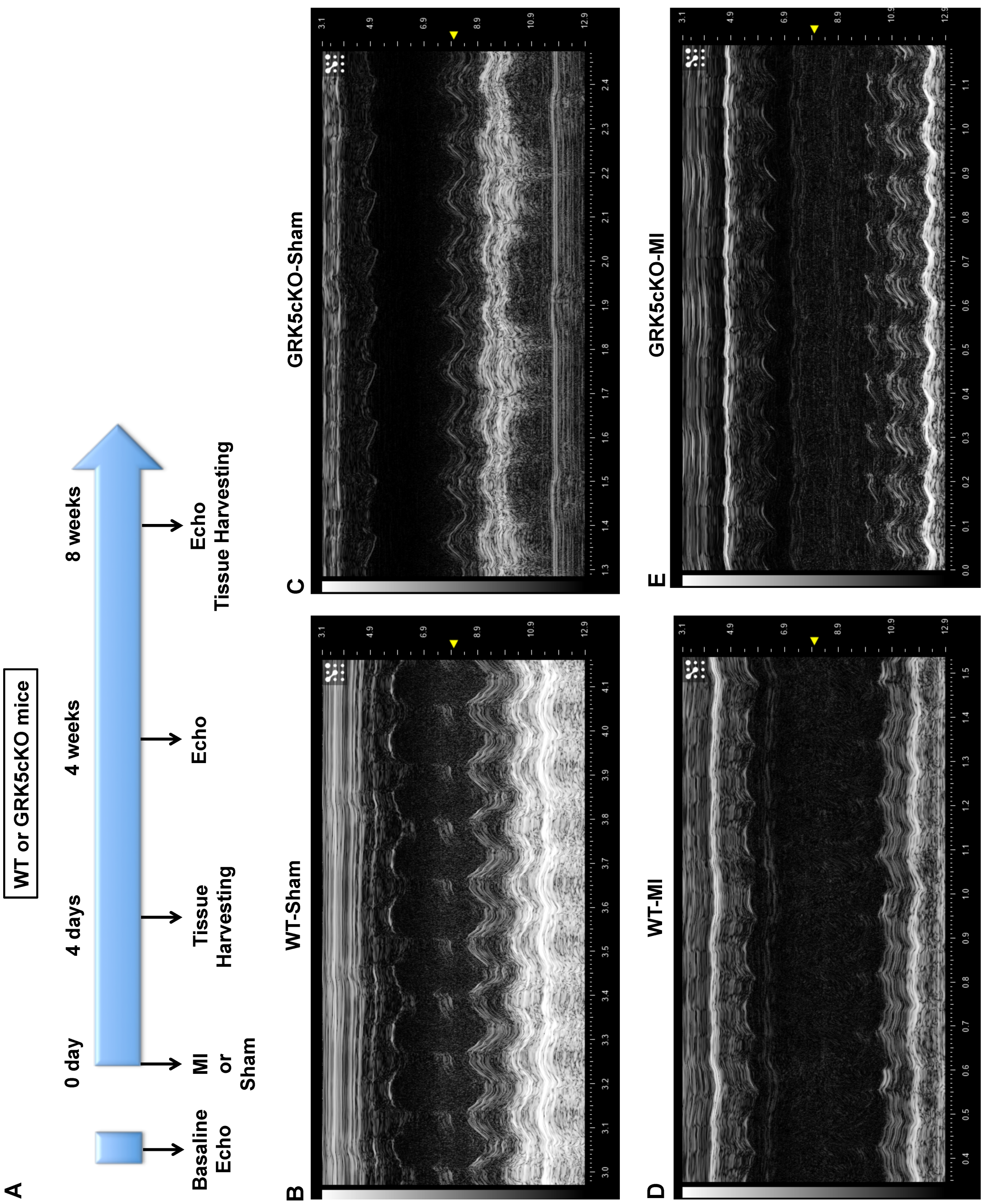

Suppl. Figure 30

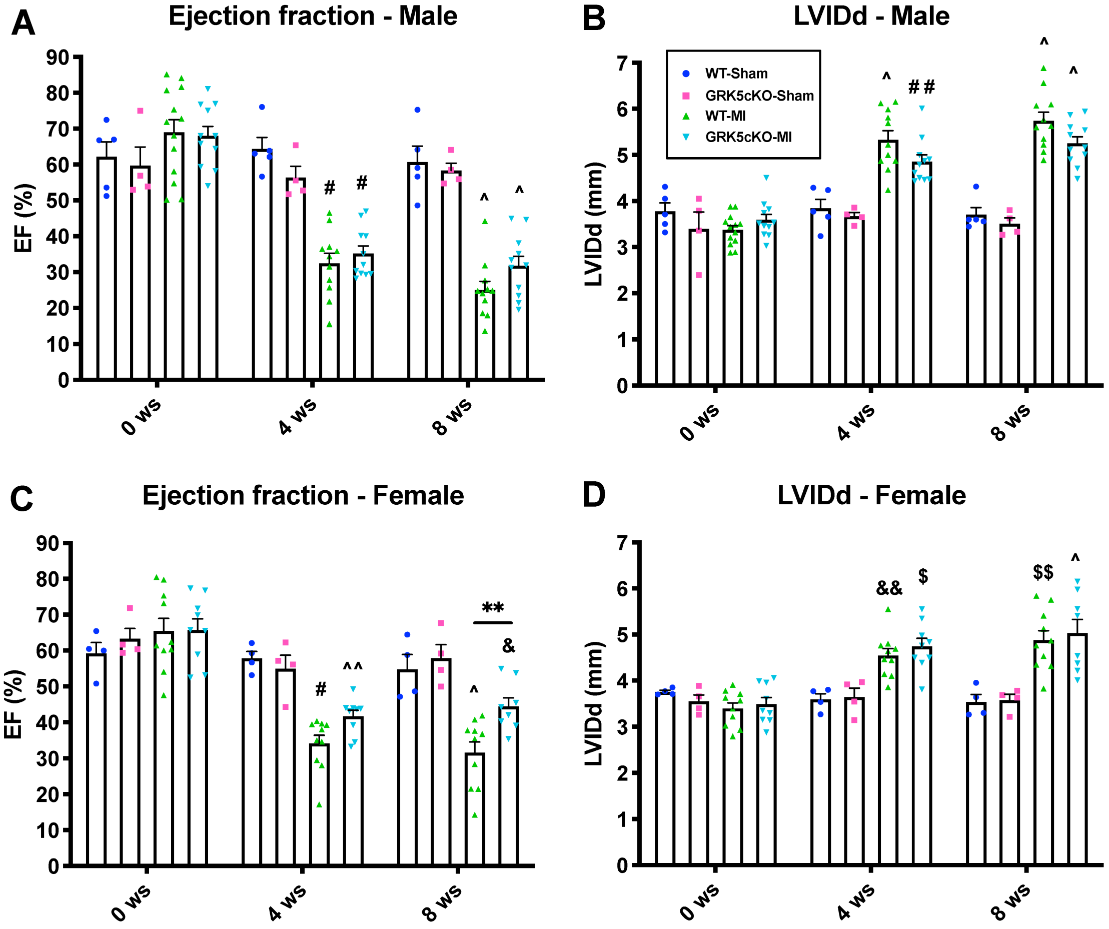

Suppl. Figure 31

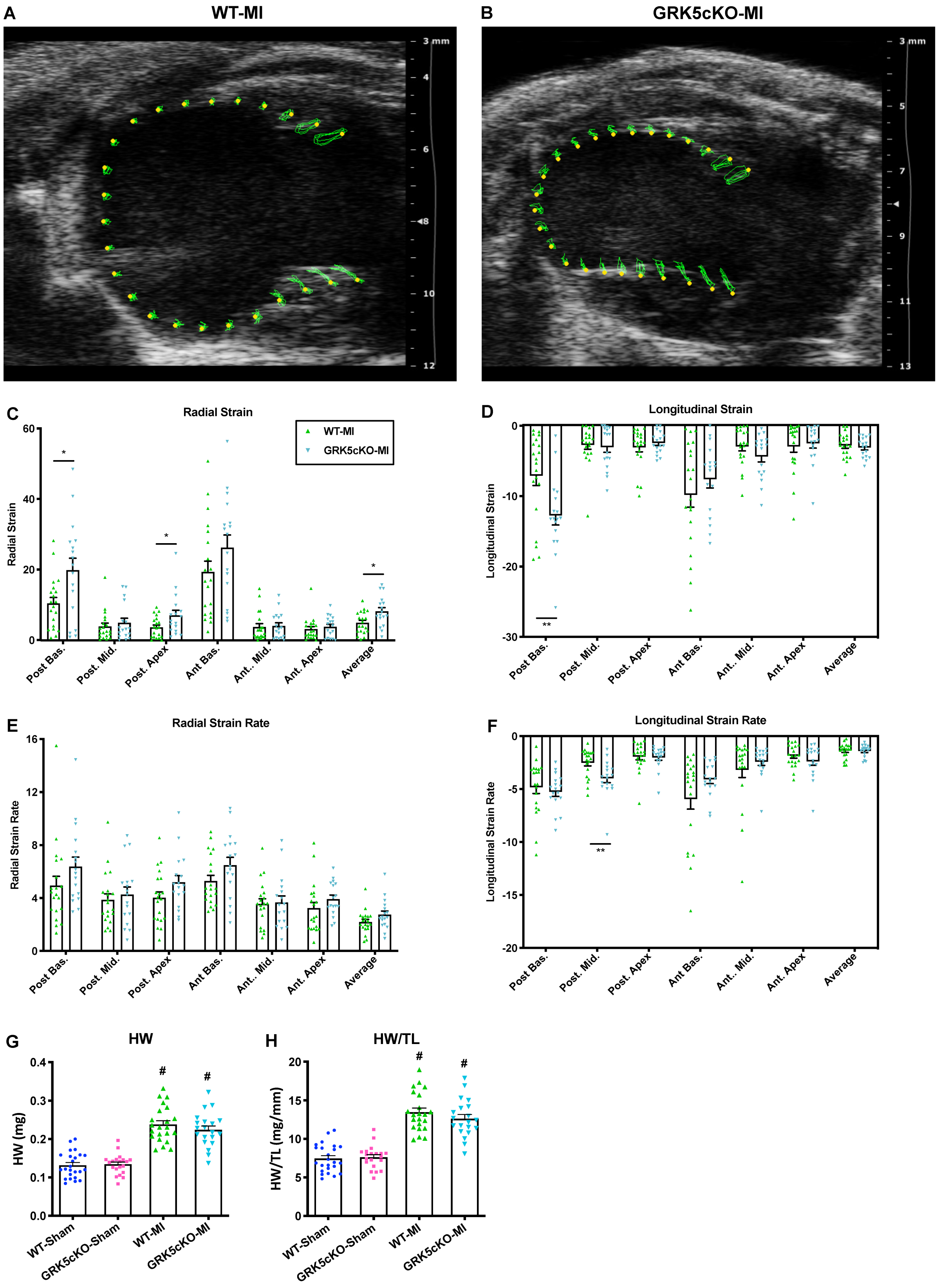

Suppl. Figure 32

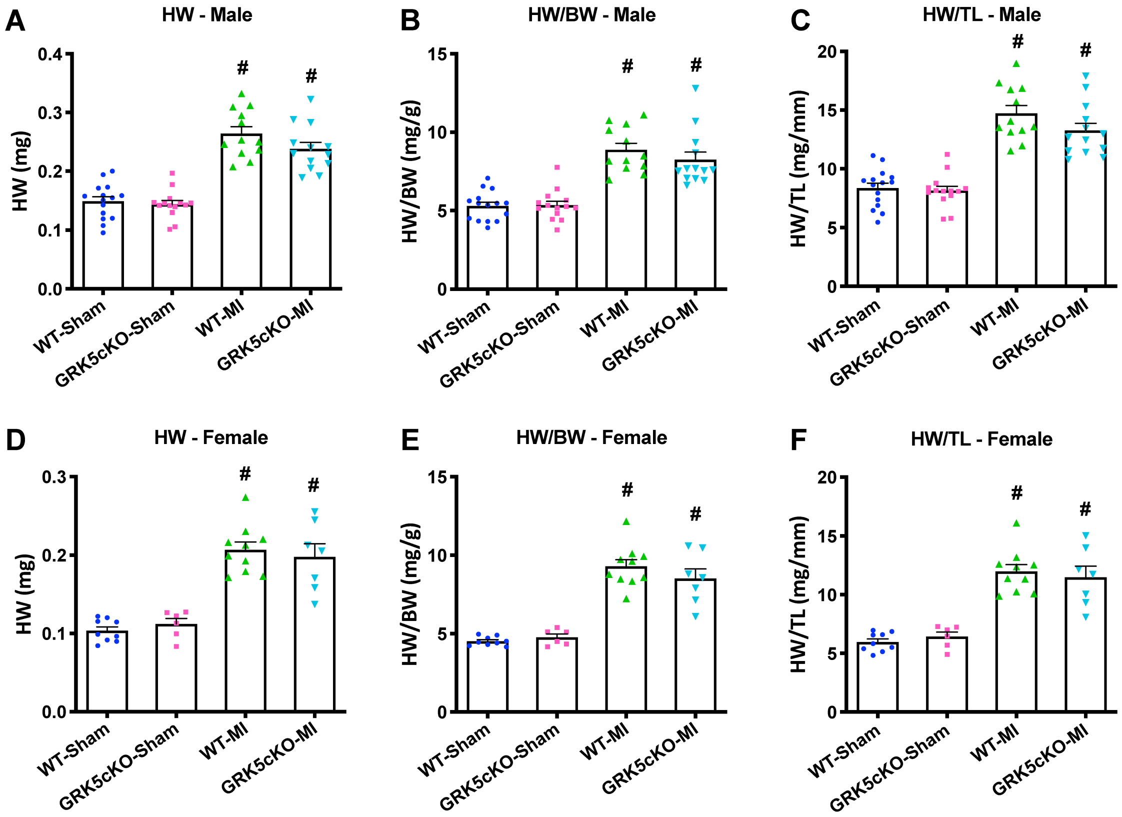

Suppl. Figure 33

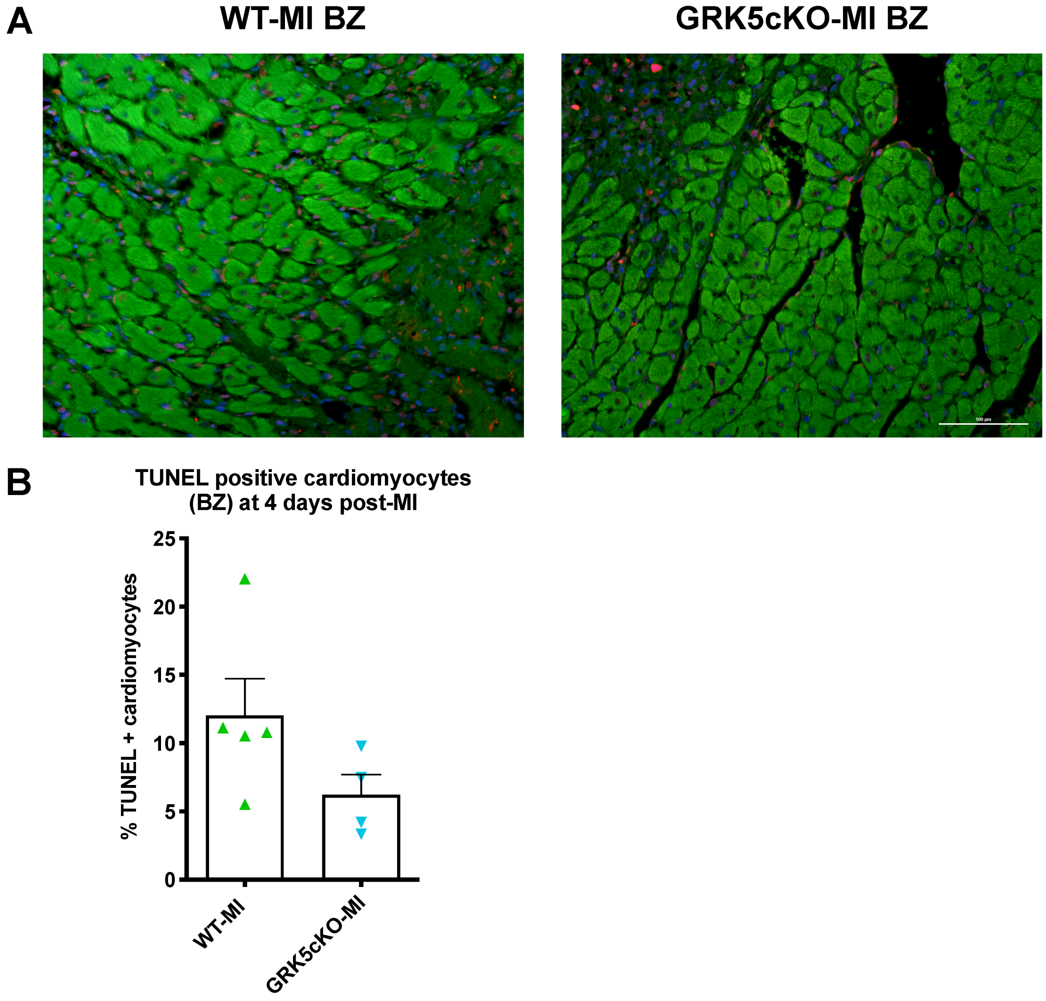

Suppl. Figure 34

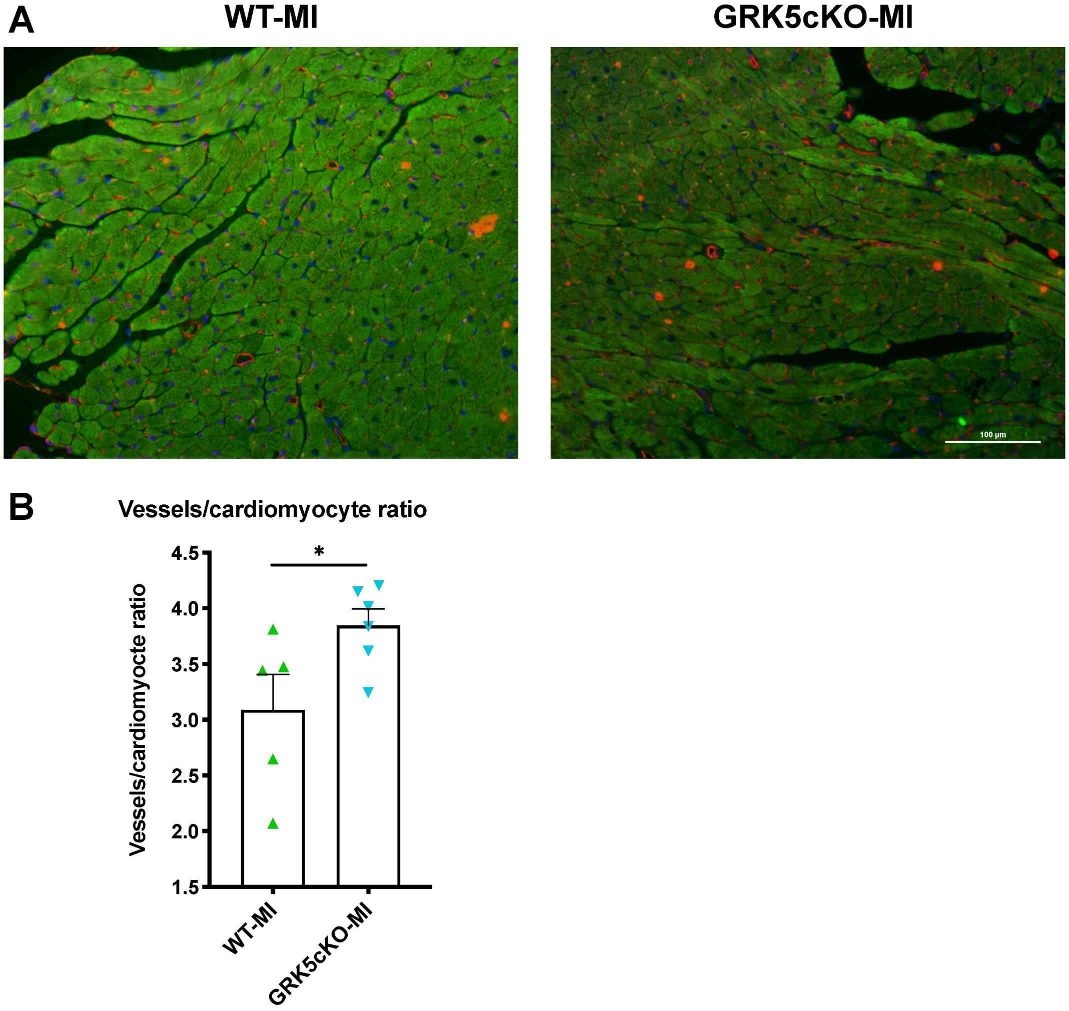

Suppl. Figure 35

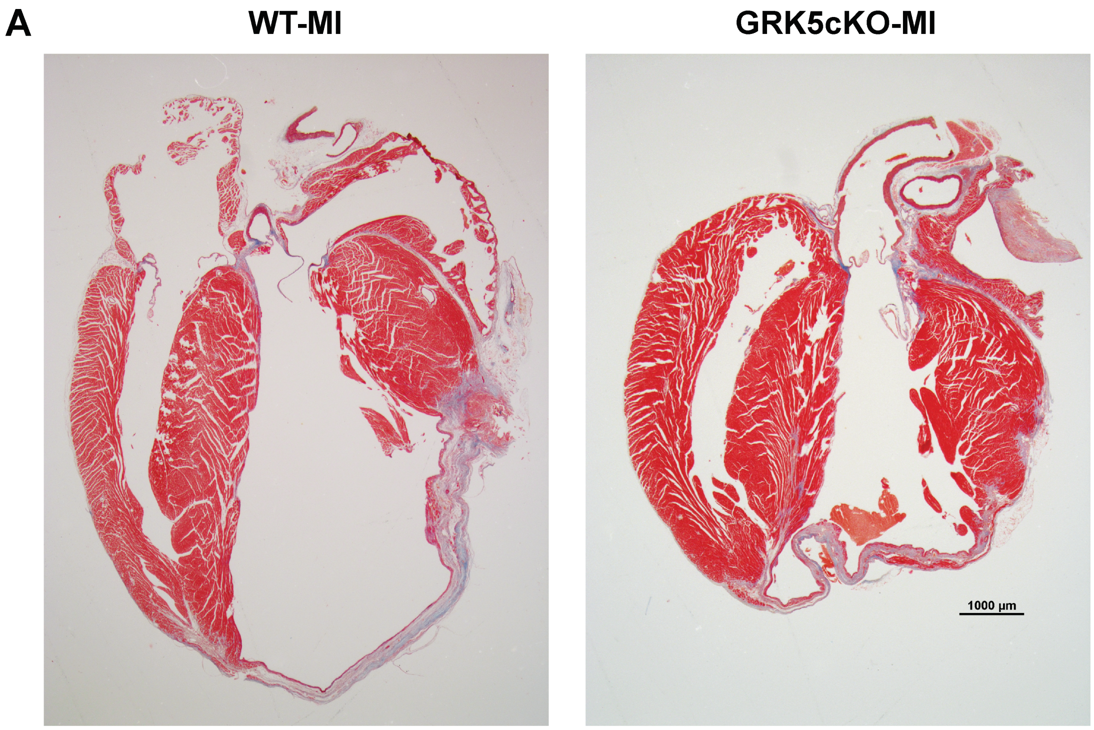

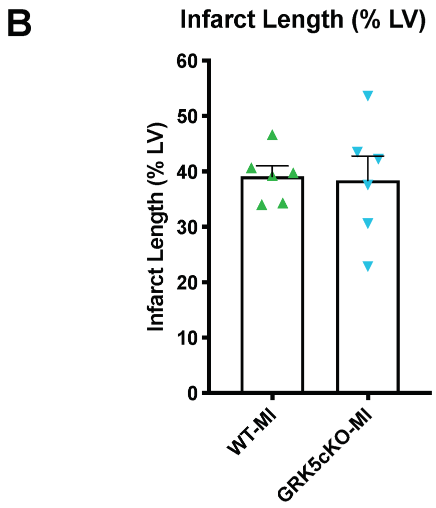

Suppl. Figure 36

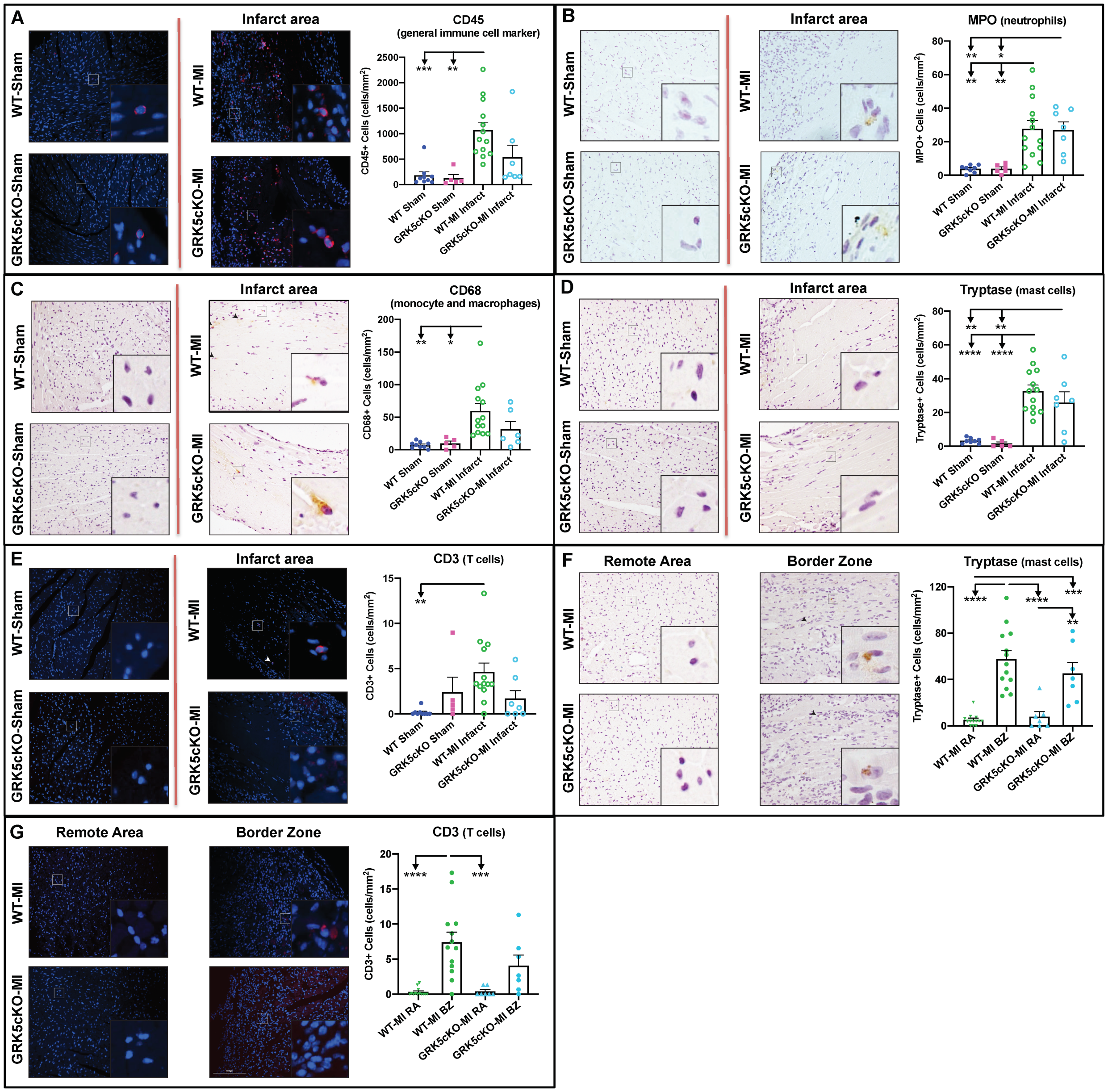

Suppl. Figure 37

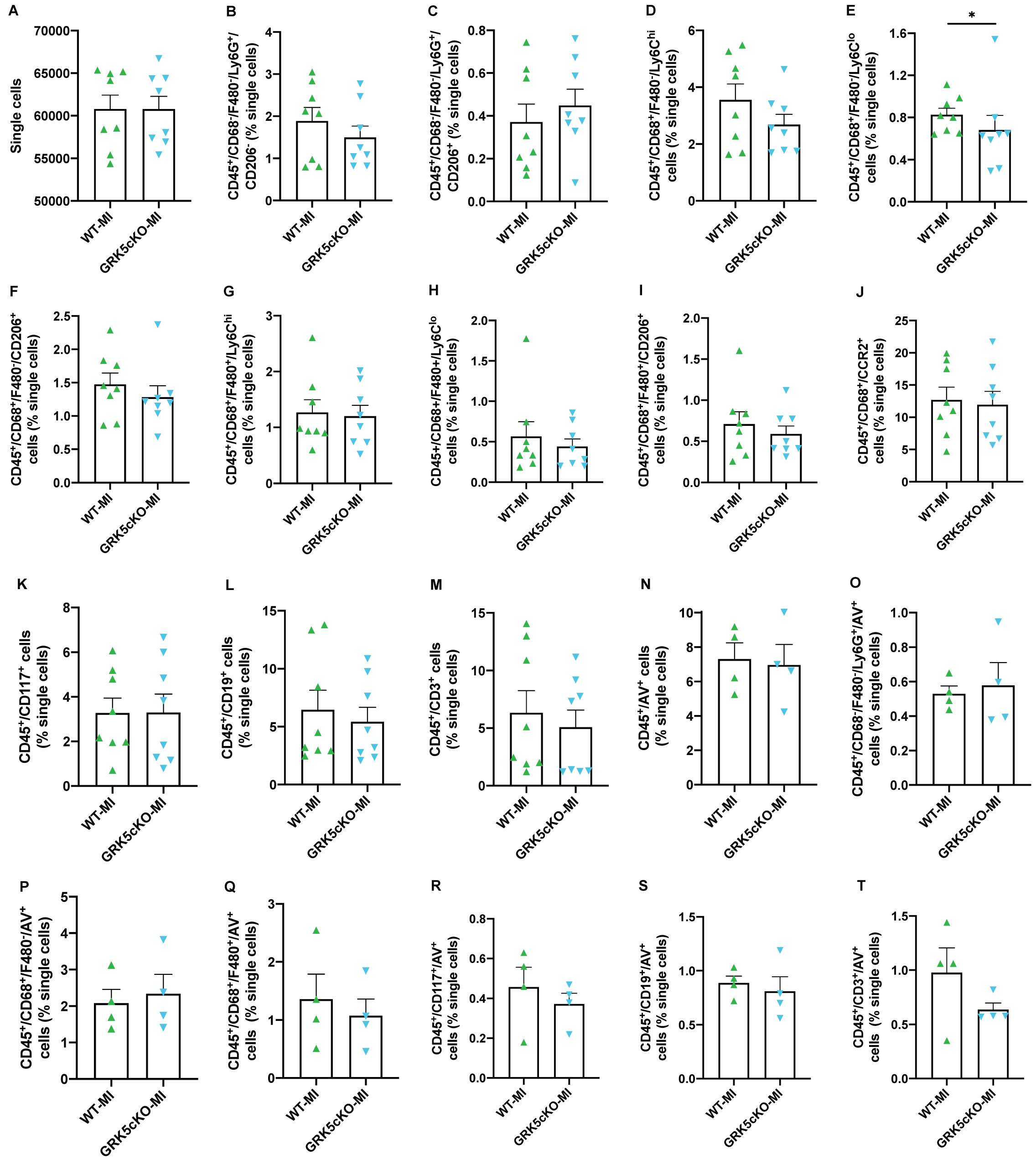

Suppl. Figure 38

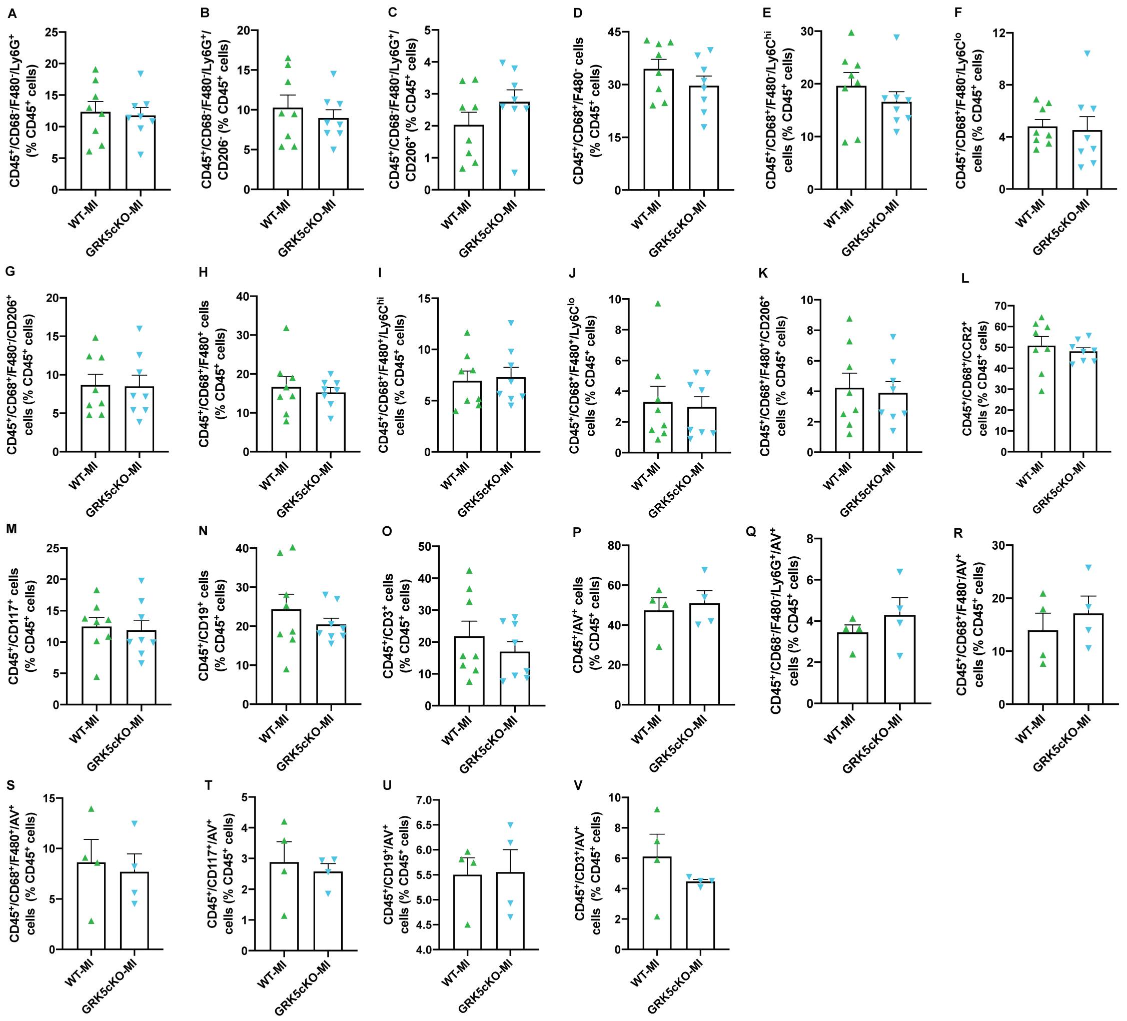

Suppl. Figure 39

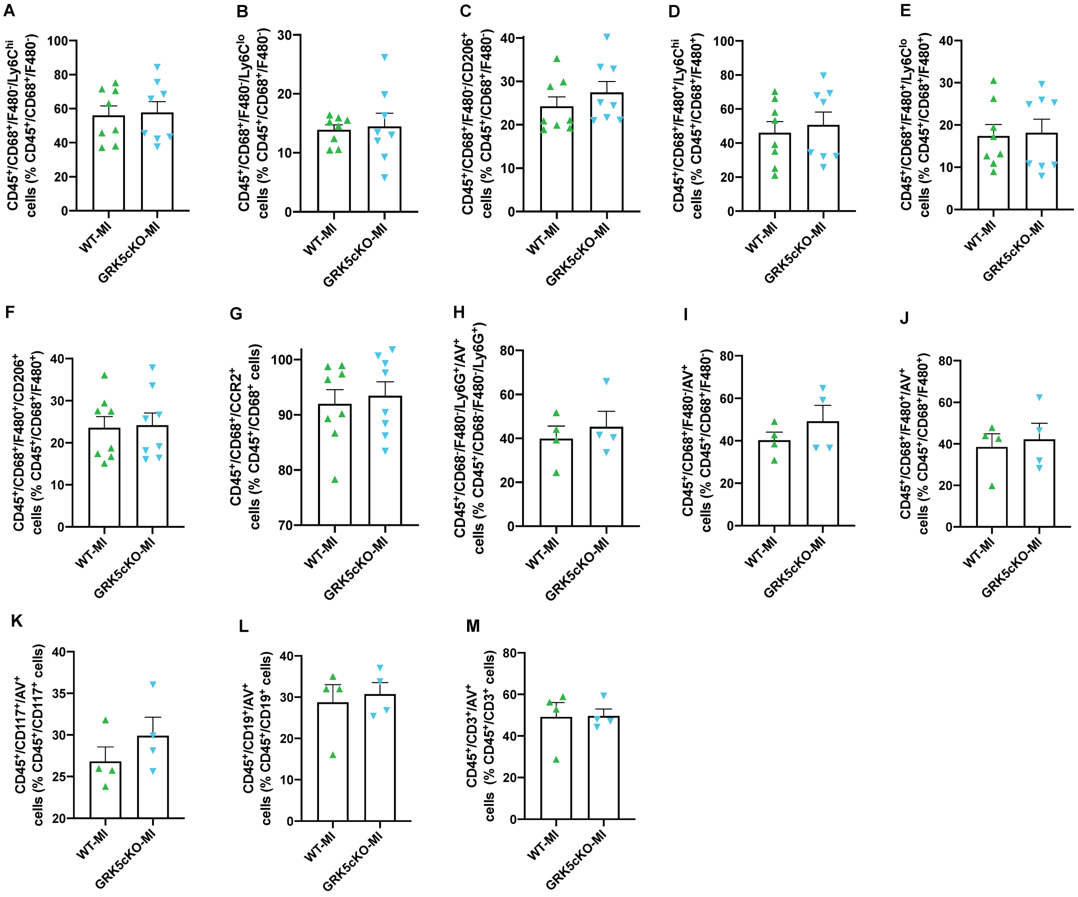

Suppl. Figure 40

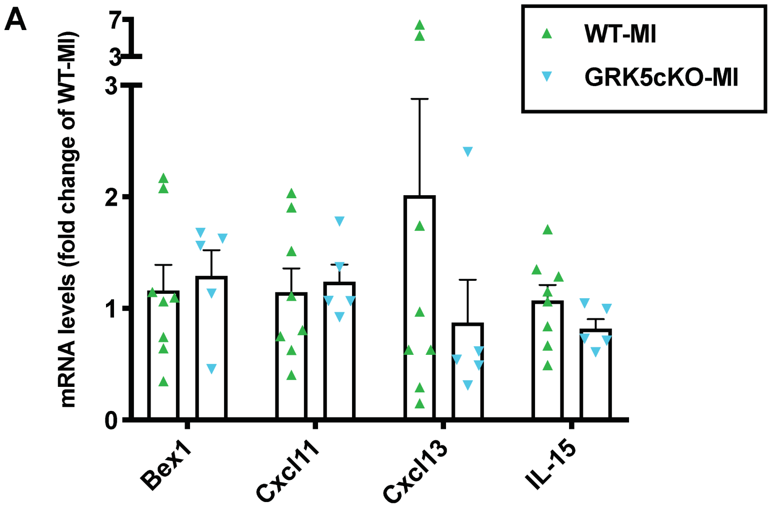

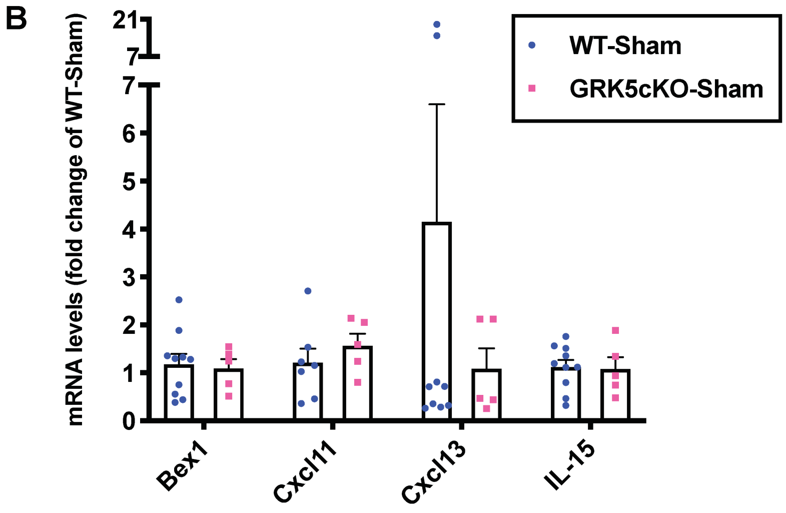

Suppl. Figure 41

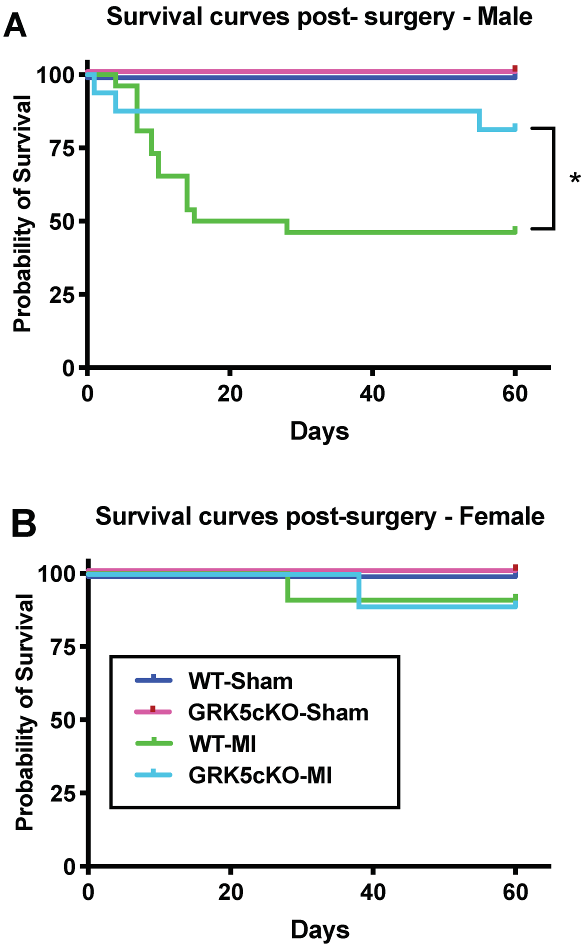

SUPPLEMENTAL TABLES:

Suppl. Table 1:

	BASELINE				4 WEEKS				8 WEEKS			
	**NLC-Sham**	**TgGRK5-Sham**	**NLC-MI**	**TgGRK5-MI**	**NLC-Sham**	**TgGRK5-Sham**	**NLC-MI**	**TgGRK5-MI**	**NLC-Sham**	**TgGRK5-Sham**	**NLC-MI**	**TgGRK5-MI**
**HR (bpm)**	472.4±15.76	469.0±13.10	483.4±9.10	474.4±9.24	454.8±14.05	476.3±14.12	485.7±9.63	474.0±9.51	502.2±12.52	492.1±12.72	501.2±10.13	486.9±14.02
**LVIDs (mm)**	2.15±0.14	2.12±0.09	2.18±0.07	2.19±0.06	2.30±0.09	2.46±0.13	4.00±0.12*	4.82±0.11*†	2.25±0.12	2.43±0.13	4.16±0.17*	5.38±0.21*†
**LVIDd (mm)**	3.41±0.11	3.44±0.11	3.43±0.07	3.46±0.05	3.59±0.08	3.77±0.12	4.79±0.11*	5.49±0.10*†	3.55±0.09	3.75±0.11	4.91±0.16*	5.95±0.18*†
**Vol s (uL)**	17.08±3.02	15.33±1.65	16.81±1.37	16.76±1.15	18.90±1.73	22.47±2.77	72.35±5.07*	110.88±6.23*†	18.41±2.66	21.97±2.86	80.84±6.89*	146.30±12.18*†
**Vol d (uL)**	49.12±3.96	49.74±4.05	49.44±2.30	50.29±1.85	54.80±2.92	61.81±4.35	109.15±5.74*	148.86±6.15*†	53.65±3.46	60.99±4.31	117.45±8.31*	181.29±12.18*†
**EF (%)**	68.12±2.91	69.59±1.35	67.11±1.49	67.23±1.51	66.31±1.78	65.06±2.23	35.03±1.91*	26.27±1.75*‡	67.31±2.62	65.59±2.37	33.14±2.00*	21.53±1.98*§
**FS (%)**	37.81±2.08	38.40±1.09	36.71±1.14	36.95±1.14	36.09±1.30	35.31±1.63	16.89±1.00*	12.47±0.90*‖	37.21±1.97	35.75±1.74	15.89±1.04*	10.10±0.98*‡

Echocardiographic data from TgGRK5 and NLC mice subjected to MI or Sham operation. Left ventricular (LV) ejection fraction (EF), fractional shortening (FS), internal diameter in systole (LVIDs) and diastole (LVIDd), volume in systole (Vol s) and diastole (Vol d), and heart rate (HR) were evaluated in NLC and TgGRK5 mice at baseline and at 4 and 8 weeks post-myocardial infarction (MI) or sham operation. Data are shown as mean±S.E.M. *p<0.0001 vs NLC-Sham and TgGRK5-Sham; †p<0.0001 vs NLC-MI; ‡p<0.01 vs NLC-MI; §p<0.001 vs NLC-MI; ||p<0.05 vs NLC-MI. Two-way ANOVA with Tukey's post hoc test was used between groups.

Suppl. Table 2. Genes upregulated in cardiomyocytes isolated at 4 days post-myocardial infarction in TgGRK5 mice compared to NCL mice.

Ensemble ID	Gene	Description	TgGRK5/ NLC fold	p-value
ENSMUSG00000063696	Gm8730	predicted pseudogene 8730	20.554	4.16E-06
ENSMUSG00000003228	Grk5	G protein-coupled receptor kinase 5	18.684	1.16E-50
ENSMUSG00000082475	Gm7206	predicted pseudogene 7206	7.577	0.003437938
ENSMUSG00000050071	Bex1	brain expressed X-linked 1	7.570	1.87E-18
ENSMUSG00000109870	Gm35850	predicted gene, 35850	6.064	6.87E-12
ENSMUSG00000067242	Lgi1	leucine-rich repeat LGI family, member 1	4.337	9.49E-06
ENSMUSG00000054034	Tceal5	transcription elongation factor A (SII)-like 5	3.809	6.71E-07
ENSMUSG00000079428	Tceal7	transcription elongation factor A (SII)-like 7	3.620	8.12E-09
ENSMUSG00000034009	Rxfp1	relaxin/insulin-like family peptide receptor 1	3.586	3.45E-07
ENSMUSG00000074736	Syndig1	synapse differentiation inducing 1	3.137	7.60E-06
ENSMUSG00000022449	Adamts20	a disintegrin-like and metallopeptidase (reprolysin type) with thrombospondin type 1 motif, 20	3.045	5.93E-05
ENSMUSG00000038801	Scgb1c1	secretoglobin, family 1C, member 1	3.002	5.84E-07
ENSMUSG00000021314	Amph	amphiphysin	2.933	5.10E-08
ENSMUSG00000041798	Gck	glucokinase	2.923	6.37E-07
ENSMUSG00000023284	Zfp605	zinc finger protein 605	2.920	8.17E-10
ENSMUSG00000050241	Klre1	killer cell lectin-like receptor family E member 1	2.750	4.73E-05
ENSMUSG00000023439	Gnb3	guanine nucleotide binding protein (G protein), beta 3	2.745	0.000207194
ENSMUSG00000097652	Mhrt	myosin heavy chain associated RNA transcript	2.654	5.74E-06
ENSMUSG00000084819	Gm11967	predicted gene 11967	2.631	2.50E-06
ENSMUSG00000033849	B3galt2	UDP-Gal:betaGlcNAc beta 1,3-galactosyltransferase, polypeptide 2	2.580	0.000310477
ENSMUSG00000055114	Anxa13	annexin A13	2.559	0.000405163
ENSMUSG00000034472	Rasd2	RASD family, member 2	2.552	0.000104723
ENSMUSG00000029053	Prkcz	protein kinase C, zeta	2.499	0.004007464
ENSMUSG00000052133	Sema5b	sema domain, seven thrombospondin repeats (type 1 and type 1-like), transmembrane domain (TM) and short cytoplasmic domain, (semaphorin) 5B	2.408	6.59E-06
ENSMUSG00000023078	Cxcl13	chemokine (C-X-C motif) ligand 13	2.400	0.00103746

ENSMUSG00000020102	Slc16a7	solute carrier family 16 (monocarboxylic acid transporters), member 7	2.361	2.22E-07
ENSMUSG00000039639	Kcne1	potassium voltage-gated channel, Isk-related subfamily, member 1	2.348	1.69E-10
ENSMUSG00000109754	Gm39214	predicted gene, 39214	2.324	0.001104177
ENSMUSG00000048782	Insc	INSC spindle orientation adaptor protein	2.292	0.005571099
ENSMUSG00000012123	Crybg2	crystallin beta-gamma domain containing 2	2.260	0.000628583
ENSMUSG00000043857	Mgat5b	mannoside acetylglucosaminyltransferase 5, isoenzyme B	2.238	0.000810913
ENSMUSG00000000183	Fgf6	fibroblast growth factor 6	2.233	0.000329195
ENSMUSG00000000305	Cdh4	cadherin 4	2.209	0.001631233
ENSMUSG00000039714	Cplx3	complexin 3	2.199	0.000477104
ENSMUSG00000029452	Tmem116	transmembrane protein 116	2.185	0.000141465
ENSMUSG00000025912	Mybl1	myeloblastosis oncogene-like 1	2.126	0.000290387
ENSMUSG00000084803	5830444B04Rik	RIKEN cDNA 5830444B04 gene	2.109	0.000231649
ENSMUSG00000108634	Gm38534	predicted gene, 38534	2.066	0.000589983
ENSMUSG00000092286	Dtnbos	dystrobrevin, beta, opposite strand	2.064	0.006696768
ENSMUSG00000060183	Cxcl11	chemokine (C-X-C motif) ligand 11	2.035	0.018806399
ENSMUSG00000097863	1010001B22Rik	RIKEN cDNA 1010001B22 gene	2.015	0.006448148
ENSMUSG00000021747	4930452B06Rik	RIKEN cDNA 4930452B06 gene	2.005	0.000520861
ENSMUSG00000064307	Lrrc51	leucine rich repeat containing 51	1.993	5.20E-05
ENSMUSG00000061322	Dnaic1	dynein, axonemal, intermediate chain 1	1.971	0.005448223
ENSMUSG00000023169	Slc38a1	solute carrier family 38, member 1	1.948	0.000337896
ENSMUSG00000021638	Ocln	occludin	1.928	0.005920521
ENSMUSG00000067338	Tuba3b	tubulin, alpha 3B	1.928	0.004051959
ENSMUSG00000051159	Cited1	Cbp/p300-interacting transactivator with Glu/Asp-rich carboxy-terminal domain 1	1.923	0.017843375
ENSMUSG00000102059	Gm20257	predicted gene, 20257	1.919	0.00118044
ENSMUSG00000092074	Dynlt1a	dynein light chain Tctex-type 1A	1.909	0.02571061
ENSMUSG00000047342	Zfp286	zinc finger protein 286	1.904	0.029386221
ENSMUSG00000030134	Rasgef1a	RasGEF domain family, member 1A	1.884	0.002011938
ENSMUSG00000056271	Lman1l	lectin, mannose-binding 1 like	1.878	0.001671903
ENSMUSG00000002324	Rec8	REC8 meiotic recombination protein	1.871	0.00360156
ENSMUSG00000113634	Gm36756	predicted gene, 36756	1.871	0.004045363
ENSMUSG00000040557	Mettl27	methyltransferase like 27	1.867	0.009050026

ENSMUSG00000049670	Morn4	MORN repeat containing 4	1.863	0.002195847
ENSMUSG00000039954	Stk32a	serine/threonine kinase 32A	1.862	0.014878277
ENSMUSG00000083411	Rpl30-ps10	ribosomal protein L30, pseudogene 10	1.857	0.019706916
ENSMUSG00000030075	Cntn3	contactin 3	1.845	0.002746216
ENSMUSG00000114061	Gm48626	predicted gene, 48626	1.840	0.002748417
ENSMUSG00000079022	Col22a1	collagen, type XXII, alpha 1	1.837	0.002935163
ENSMUSG00000054428	Atpif1	ATPase inhibitory factor 1	1.833	0.037248142
ENSMUSG00000024177	Nme4	NME/NM23 nucleoside diphosphate kinase 4	1.832	2.70E-05
ENSMUSG00000072762	4930522L14Rik	RIKEN cDNA 4930522L14 gene	1.812	9.78E-05
ENSMUSG00000063297	Luzp2	leucine zipper protein 2	1.807	0.005925596
ENSMUSG00000063445	Nmral1	NmrA-like family domain containing 1	1.802	8.38E-05
ENSMUSG00000024846	Cst6	cystatin E/M	1.802	0.000389936
ENSMUSG00000034353	Ramp1	receptor (calcitonin) activity modifying protein 1	1.792	0.014148281
ENSMUSG00000039154	Shd	src homology 2 domain-containing transforming protein D	1.791	0.019777224
ENSMUSG00000019194	Scn1b	sodium channel, voltage-gated, type I, beta	1.780	0.001966565
ENSMUSG00000061815	Rufy4	RUN and FYVE domain containing 4	1.771	0.001707788
ENSMUSG00000085787	Gm13092	predicted gene 13092	1.761	0.010563077
ENSMUSG00000035509	Fbxl21	F-box and leucine-rich repeat protein 21	1.751	0.007400962
ENSMUSG00000040205	Cuzd1	CUB and zona pellucida-like domains 1	1.736	0.003468271
ENSMUSG00000016024	Lbp	lipopolysaccharide binding protein	1.733	0.002357246
ENSMUSG00000021636	Marveld2	MARVEL (membrane-associating) domain containing 2	1.727	0.004604897
ENSMUSG00000075334	Rprm	reprimo, TP53 dependent G2 arrest mediator candidate	1.725	0.00828779
ENSMUSG00000097123	Gm6297	predicted gene 6297	1.720	0.014457902
ENSMUSG00000041782	Lad1	ladinin	1.718	0.007899878
ENSMUSG00000037106	Fer1l6	fer-1-like 6 (C. elegans)	1.718	0.007865828
ENSMUSG00000086915	Gm16365	predicted gene 16365	1.714	0.024882567
ENSMUSG00000030787	Lyve1	lymphatic vessel endothelial hyaluronan receptor 1	1.697	4.04E-05
ENSMUSG00000087651	1500009L16Rik	RIKEN cDNA 1500009L16 gene	1.697	9.22E-05
ENSMUSG00000036091	Hyal3	hyaluronoglucosaminidase 3	1.693	0.039236057
ENSMUSG00000109696	Gm36670	predicted gene, 36670	1.691	0.004207518
ENSMUSG00000034209	Rasl10a	RAS-like, family 10, member A	1.687	0.001008483
ENSMUSG00000022681	Ntan1	N-terminal Asn amidase	1.685	0.00084155

ENSMUSG00000085087	Gm13528	predicted gene 13528	1.681	0.018352913
ENSMUSG00000066705	Fxyd6	FXYD domain-containing ion transport regulator 6	1.678	0.010717481
ENSMUSG00000089768	Tmsb15b1	thymosin beta 15b1	1.676	0.017038802
ENSMUSG00000086712	AI427809	expressed sequence AI427809	1.665	0.035443132
ENSMUSG00000095686	Gm3099	predicted gene 3099	1.653	0.002681296
ENSMUSG00000086361	Gm6569	predicted gene 6569	1.650	0.015113267
ENSMUSG00000071547	Nt5dc2	5'-nucleotidase domain containing 2	1.643	0.000338327
ENSMUSG00000057897	Camk2b	calcium/calmodulin-dependent protein kinase II, beta	1.638	0.003855294
ENSMUSG00000000326	Comt	catechol-O-methyltransferase	1.635	0.00080777
ENSMUSG00000093106	Gm25534	predicted gene, 25534	1.635	0.006655001
ENSMUSG00000020431	Adcy1	adenylate cyclase 1	1.634	0.021226734
ENSMUSG00000008999	Bmp7	bone morphogenetic protein 7	1.629	0.018240547
ENSMUSG00000057286	St6galnac2	ST6 (alpha-N-acetyl-neuraminyl-2,3-beta-galactosyl-1,3)-N-acetylgalactosaminide alpha-2,6-sialyltransferase 2	1.622	0.000175407
ENSMUSG00000025584	Pde8a	phosphodiesterase 8A	1.622	0.00784169
ENSMUSG00000086363	A330102I10Rik	RIKEN cDNA A330102I10 gene	1.616	0.044909077
ENSMUSG00000032172	Olfm2	olfactomedin 2	1.615	0.03035321
ENSMUSG00000053168	9030619P08Rik	RIKEN cDNA 9030619P08 gene	1.611	0.02578538
ENSMUSG00000006218	Fam131c	family with sequence similarity 131, member C	1.605	0.022774604
ENSMUSG00000069733	Ube2u	ubiquitin-conjugating enzyme E2U (putative)	1.605	0.024961605
ENSMUSG00000097047	1110020A21Rik	RIKEN cDNA 1110020A21 gene	1.603	0.014193348
ENSMUSG00000059149	Mfsd4a	major facilitator superfamily domain containing 4A	1.597	0.011276501
ENSMUSG00000097489	Gm26808	predicted gene, 26808	1.596	0.047866017
ENSMUSG00000085927	Gm15888	predicted gene 15888	1.595	0.009506993
ENSMUSG00000050035	Fhl4	four and a half LIM domains 4	1.591	0.042091841
ENSMUSG00000097776	Gm26795	predicted gene, 26795	1.590	0.018020819
ENSMUSG00000044206	Vsig4	V-set and immunoglobulin domain containing 4	1.580	0.012866414
ENSMUSG00000086368	Gm13830	predicted gene 13830	1.578	0.015336948
ENSMUSG00000035439	Haus8	4HAUS augmin-like complex, subunit 8	1.578	0.000834482
ENSMUSG00000038879	Nipal2	NIPA-like domain containing 2	1.577	0.017003843
ENSMUSG00000015354	Pcolce2	procollagen C-endopeptidase enhancer 2	1.573	0.006256456
ENSMUSG00000032725	Folr2	folate receptor 2 (fetal)	1.572	0.038532486

ENSMUSG00000001891	Ugp2	UDP-glucose pyrophosphorylase 2	1.571	0.001638679
ENSMUSG00000020534	Shmt1	serine hydroxymethyltransferase 1 (soluble)	1.564	0.02027742
ENSMUSG00000024109	Nrxn1	neurexin I	1.564	0.022156369
ENSMUSG00000035062	Zc4h2	zinc finger, C4H2 domain containing	1.563	0.035318738
ENSMUSG00000045678	Olfr1489	olfactory receptor 1489	1.563	0.041860101
ENSMUSG00000104719	4933437G19Rik	RIKEN cDNA 4933437G19 gene	1.561	0.020556483
ENSMUSG00000025739	Gng13	guanine nucleotide binding protein (G protein), gamma 13	1.560	0.01666515
ENSMUSG00000035232	Pdk3	pyruvate dehydrogenase kinase, isoenzyme 3	1.559	0.047875934
ENSMUSG00000069094	Pde7a	phosphodiesterase 7A	1.558	0.004157954
ENSMUSG00000048899	Rimkla	ribosomal modification protein rimK-like family member A	1.553	0.038611147
ENSMUSG00000075394	Hoxc4	homeobox C4	1.553	0.030805357
ENSMUSG00000007646	Rad51c	RAD51 paralog C	1.552	0.006057001
ENSMUSG00000109817	Gm10683	predicted gene 10683	1.551	0.037423408
ENSMUSG00000037188	Grhl3	grainyhead like transcription factor 3	1.550	0.028579891
ENSMUSG00000030793	Pycard	PYD and CARD domain containing	1.545	0.000170468
ENSMUSG00000021932	Rnaseh2b	ribonuclease H2, subunit B	1.542	1.15E-05
ENSMUSG00000039899	Fgl2	fibrinogen-like protein 2	1.542	0.011987476
ENSMUSG00000112249	Gm30262	predicted gene, 30262	1.542	0.016343158
ENSMUSG00000023963	Cyp39a1	cytochrome P450, family 39, subfamily a, polypeptide 1	1.541	7.82E-05
ENSMUSG00000079339	Ifit1bl1	interferon induced protein with tetratricpeptide repeats 1B like 1	1.541	0.002572519
ENSMUSG00000033488	Cryzl2	crystallin zeta like 2	1.537	0.026115607
ENSMUSG00000086236	5830418P13Rik	RIKEN cDNA 5830418P13 gene	1.532	0.040898229
ENSMUSG00000085055	Gm15958	predicted gene 15958	1.530	0.032317073
ENSMUSG00000105156	Gm9057	predicted gene 9057	1.524	0.031156103
ENSMUSG00000019851	Perp	PERP, TP53 apoptosis effector	1.524	0.000965867
ENSMUSG00000027792	Bche	butyrylcholinesterase	1.518	0.002873374
ENSMUSG00000076068	Mir678	microRNA 678	1.515	0.041894203
ENSMUSG00000104195	B230377A18Rik	RIKEN cDNA B230377A18 gene	1.514	0.034755575
ENSMUSG00000039913	Pak7	p21 (RAC1) activated kinase 7	1.512	0.027654431
ENSMUSG00000059060	Rad51b	RAD51 paralog B	1.509	0.019127439
ENSMUSG00000089718	2310075C17Rik	RIKEN cDNA 2310075C17 gene	1.505	0.023770672
ENSMUSG00000085271	E130006D01Rik	RIKEN cDNA E130006D01 gene	1.504	0.023361969

ENSMUSG00000038665	Dgki	diacylglycerol kinase, iota	1.499	0.02980791
ENSMUSG00000097045	Gm26641	predicted gene, 26641	1.495	0.035140694
ENSMUSG00000042312	S100a13	S100 calcium binding protein A13	1.490	0.000212753
ENSMUSG00000028933	Xrcc2	X-ray repair complementing defective repair in Chinese hamster cells 2	1.487	0.002401364
ENSMUSG00000050379	Sept6	septin 6	1.487	0.026456685
ENSMUSG00000030474	Siglece	sialic acid binding Ig-like lectin E	1.486	0.030851757
ENSMUSG00000040726	Hesx1	homeobox gene expressed in ES cells	1.483	0.024249952
ENSMUSG00000031230	Fgf16	fibroblast growth factor 16	1.481	0.045999797
ENSMUSG00000030946	Lhpp	phospholysine phosphohistidine inorganic pyrophosphate phosphatase	1.479	0.009504945
ENSMUSG00000028158	Mttp	microsomal triglyceride transfer protein	1.479	0.004463862
ENSMUSG00000110156	Gm42067	predicted gene, 42067	1.477	0.047214149
ENSMUSG00000089669	Tnfsf13	tumor necrosis factor (ligand) superfamily, member 13	1.476	0.012285207
ENSMUSG00000042249	Grk3	G protein-coupled receptor kinase 3	1.473	0.000688734
ENSMUSG00000028780	Sema3c	sema domain, immunoglobulin domain (Ig), short basic domain, secreted, (semaphorin) 3C	1.472	0.002323354
ENSMUSG00000004032	Gstm5	glutathione S-transferase, mu 5	1.469	0.005529303
ENSMUSG00000097062	Gm17586	predicted gene, 17586	1.462	0.020880904
ENSMUSG00000029134	Plb1	phospholipase B1	1.454	0.044415389
ENSMUSG00000100750	Gm29084	predicted gene 29084	1.452	0.002534017
ENSMUSG00000025855	Prkar1b	protein kinase, cAMP dependent regulatory, type I beta	1.445	0.03383511
ENSMUSG00000027018	Hat1	histone aminotransferase 1	1.436	0.005375495
ENSMUSG00000053469	Tg	thyroglobulin	1.433	0.011971624
ENSMUSG00000068854	Hist2h2be	histone cluster 2, H2be	1.431	0.033372941
ENSMUSG00000055044	Pdlim1	PDZ and LIM domain 1 (elfin)	1.430	0.036246188
ENSMUSG00000057531	Dtnbp1	dystrobrevin binding protein 1	1.428	0.027846956
ENSMUSG00000051154	Commd3	COMM domain containing 3	1.420	0.034465358
ENSMUSG00000097563	Gm26638	predicted gene, 26638	1.419	0.021821638
ENSMUSG00000021391	Cenpp	centromere protein P	1.415	0.009271743
ENSMUSG00000025395	Prim1	DNA primase, p49 subunit	1.415	0.012875195
ENSMUSG00000032698	Lmo2	LIM domain only 2	1.412	0.004647463
ENSMUSG00000027953	Slc50a1	solute carrier family 50 (sugar transporter), member 1	1.411	0.001984303
ENSMUSG00000082746	Rps12-ps1	ribosomal protein S12, pseudogene 1	1.408	0.042111403

ENSMUSG00000087344	Gm15627	predicted gene 15627	1.407	0.038130942
ENSMUSG00000057329	Bcl2	B cell leukemia/lymphoma 2	1.406	0.020203663
ENSMUSG00000038776	Ephx1	epoxide hydrolase 1, microsomal	1.406	0.007994752
ENSMUSG00000087579	Hectd2os	Hectd2, opposite strand	1.398	0.042001333
ENSMUSG00000000295	Hddc2	HD domain containing 2	1.394	0.01125243
ENSMUSG00000062619	2310039H08Rik	RIKEN cDNA 2310039H08 gene	1.392	0.002701277
ENSMUSG00000024556	Me2	malic enzyme 2, NAD(+)-dependent, mitochondrial	1.387	0.003343986
ENSMUSG00000027173	Depdc7	DEP domain containing 7	1.386	0.016746691
ENSMUSG00000026778	Prkcq	protein kinase C, theta	1.381	0.019033665
ENSMUSG00000099812	Gm28515	predicted gene 28515	1.379	0.036828462
ENSMUSG00000001999	Blvra	biliverdin reductase A	1.376	0.01427052
ENSMUSG00000039345	Mettl22	methyltransferase like 22	1.374	0.031054902
ENSMUSG00000045827	Serpinb9	serine (or cysteine) peptidase inhibitor, clade B, member 9	1.372	0.004622839
ENSMUSG00000012187	Mogat1	monoacylglycerol O-acyltransferase 1	1.371	0.025105278
ENSMUSG00000102722	Gm37935	predicted gene, 37935	1.370	0.049120458
ENSMUSG00000078851	Hist3h2a	histone cluster 3, H2a	1.369	0.005218667
ENSMUSG00000041840	Haus1	HAUS augmin-like complex, subunit 1	1.366	0.003446112
ENSMUSG00000025150	Cbr2	carbonyl reductase 2	1.363	0.011324781
ENSMUSG00000040466	Blvrb	biliverdin reductase B (flavin reductase (NADPH))	1.360	0.014513266
ENSMUSG00000058443	Rpl10-ps3	ribosomal protein L10, pseudogene 3	1.357	0.00808969
ENSMUSG00000049940	Pgrmc2	progesterone receptor membrane component 2	1.357	0.002727927
ENSMUSG00000111103	1700016J18Rik	RIKEN cDNA 1700016J18 gene	1.355	0.04514185
ENSMUSG00000026049	Tex30	testis expressed 30	1.353	0.017196123
ENSMUSG00000022964	Tmem50b	transmembrane protein 50B	1.351	0.016049617
ENSMUSG00000015575	Atp6v0e	ATPase, H+ transporting, lysosomal V0 subunit E	1.346	0.014599323
ENSMUSG00000043770	Gm12481	predicted gene 12481	1.343	0.041374844
ENSMUSG00000018362	Kpna2	karyopherin (importin) alpha 2	1.341	0.002577575
ENSMUSG00000062110	Scfd2	Sec1 family domain containing 2	1.334	0.004741931
ENSMUSG00000021811	Dnajc9	DnaJ heat shock protein family (Hsp40) member C9	1.333	0.001301529
ENSMUSG00000063480	Snu13	SNU13 homolog, small nuclear ribonucleoprotein (U4/U6.U5)	1.333	0.018710429
ENSMUSG00000024561	Mbd1	methyl-CpG binding domain protein 1	1.326	0.013072565
ENSMUSG00000035184	Fam124a	family with sequence similarity 124, member A	1.325	0.041213293

ENSMUSG00000096255	Dynlt1b	dynein light chain Tctex-type 1B	1.321	0.022372691
ENSMUSG00000031023	Akip1	A kinase (PRKA) interacting protein 1	1.321	0.008662274
ENSMUSG00000029802	Abcg2	ATP binding cassette subfamily G member 2 (Junior blood group)	1.315	0.002600635
ENSMUSG00000021900	Btd	biotinidase	1.312	0.047101442
ENSMUSG00000041355	Ssr2	signal sequence receptor, beta	1.310	0.016101335
ENSMUSG00000020032	Nuak1	NUAK family, SNF1-like kinase, 1	1.309	0.025892544
ENSMUSG00000025358	Cdk2	cyclin-dependent kinase 2	1.299	0.046337629
ENSMUSG00000042694	Stn1	STN1, CST complex subunit	1.298	0.013705376
ENSMUSG00000020737	Jpt1	Jupiter microtubule associated homolog 1	1.297	0.02755839
ENSMUSG00000020044	Timp3	tissue inhibitor of metalloproteinase 3	1.296	0.009579298
ENSMUSG00000018923	Med11	mediator complex subunit 11	1.296	0.033766591
ENSMUSG00000021091	Serpina3n	serine (or cysteine) peptidase inhibitor, clade A, member 3N	1.294	0.015633266
ENSMUSG00000026810	Dpm2	dolichol-phosphate (beta-D) mannosyltransferase 2	1.293	0.012470129
ENSMUSG00000021376	Tpmt	thiopurine methyltransferase	1.291	0.04686624
ENSMUSG00000032436	Cmtm7	CKLF-like MARVEL transmembrane domain containing 7	1.287	0.045461725
ENSMUSG00000027865	Gdap2	ganglioside-induced differentiation-associated-protein 2	1.286	0.043927731
ENSMUSG00000058922	Gm10052	predicted pseudogene 10052	1.285	0.037974206
ENSMUSG00000022664	Slc35a5	solute carrier family 35, member A5	1.283	0.033207069
ENSMUSG00000030591	Psmd8	proteasome (prosome, macropain) 26S subunit, non-ATPase, 8	1.283	0.000194707
ENSMUSG00000084416	Rpl10a-ps1	ribosomal protein L10A, pseudogene 1	1.282	0.016650744
ENSMUSG00000027534	Snx16	sorting nexin 16	1.279	0.048304866
ENSMUSG00000027428	Rbbp9	retinoblastoma binding protein 9, serine hydrolase	1.274	0.044039867
ENSMUSG00000004070	Hmox2	heme oxygenase 2	1.274	0.011495475
ENSMUSG00000027263	Tubgcp4	tubulin, gamma complex associated protein 4	1.273	0.044090267
ENSMUSG00000060771	Tsga10	testis specific 10	1.271	0.029848153
ENSMUSG00000028016	Ints12	integrator complex subunit 12	1.268	0.026405169
ENSMUSG00000093674	Rpl41	ribosomal protein L41	1.267	0.023390843
ENSMUSG00000013495	Tmem175	transmembrane protein 175	1.265	0.008393891
ENSMUSG00000027327	1700037H04Rik	RIKEN cDNA 1700037H04 gene	1.264	0.041913548
ENSMUSG00000017176	Nt5c3b	5'-nucleotidase, cytosolic IIIB	1.262	0.018650122
ENSMUSG00000063065	Mapk3	mitogen-activated protein kinase 3	1.254	0.020870801
ENSMUSG00000036890	Gtdc1	glycosyltransferase-like domain containing 1	1.247	0.035933685

ENSMUSG00000055850	Rnf181	ring finger protein 181	1.243	0.002941064
ENSMUSG00000028271	Gtf2b	general transcription factor IIB	1.241	0.025395119
ENSMUSG00000046034	Otulin	OTU deubiquitinase with linear linkage specificity	1.236	0.030838245
ENSMUSG00000031796	Cfap20	cilia and flagella associated protein 20	1.235	0.007276256
ENSMUSG00000029474	Rnf34	ring finger protein 34	1.235	0.032407802
ENSMUSG00000020024	Cep83	centrosomal protein 83	1.232	0.049591767
ENSMUSG00000006728	Cdk4	cyclin-dependent kinase 4	1.231	0.013318891
ENSMUSG00000022841	Ap2m1	adaptor-related protein complex 2, mu 1 subunit	1.229	0.043252779
ENSMUSG00000052102	Gnpda1	glucosamine-6-phosphate deaminase 1	1.226	0.034416988
ENSMUSG00000029759	Pon3	paraoxonase 3	1.226	0.035953359
ENSMUSG00000040822	1700123O20Rik	RIKEN cDNA 1700123O20 gene	1.224	0.026272465
ENSMUSG00000022064	Pibf1	progesterone immunomodulatory binding factor 1	1.221	0.033904402
ENSMUSG00000066979	Bub3	BUB3 mitotic checkpoint protein	1.218	0.049907832
ENSMUSG00000053565	Eif3k	eukaryotic translation initiation factor 3, subunit K	1.215	0.039400892
ENSMUSG00000035790	Cep19	centrosomal protein 19	1.214	0.044539867
ENSMUSG00000032803	Cdv3	carnitine deficiency-associated gene expressed in ventricle 3	1.214	0.03254435
ENSMUSG00000031109	Enox2	ecto-NOX disulfide-thiol exchanger 2	1.210	0.029495286
ENSMUSG00000022749	Tbc1d23	TBC1 domain family, member 23	1.209	0.014081749
ENSMUSG00000034263	Ints14	integrator complex subunit 14	1.202	0.019036769
ENSMUSG00000063800	Prpf38a	PRP38 pre-mRNA processing factor 38 (yeast) domain containing A	1.200	0.013964016
ENSMUSG00000012076	Brms1l	breast cancer metastasis-suppressor 1-like	1.200	0.023362373
ENSMUSG00000030965	Abraxas2	BRISC complex subunit	1.199	0.033720457
ENSMUSG00000058799	Nap1l1	nucleosome assembly protein 1-like 1	1.196	0.046552365
ENSMUSG00000011831	Evi5	ecotropic viral integration site 5	1.183	0.030805696
ENSMUSG00000041236	Vps41	VPS41 HOPS complex subunit	1.170	0.038917304
ENSMUSG00000017760	Ctsa	cathepsin A	1.163	0.011147987
ENSMUSG00000056234	Ncoa4	nuclear receptor coactivator 4	1.158	0.045758203
ENSMUSG00000016018	Skiv2l2	superkiller viralicidic activity 2-like 2 (S. cerevisiae)	1.157	0.049852054

Suppl. Table 3. Genes downregulated in cardiomyocytes isolated at 4 days post-myocardial infarction in TgGRK5 mice compared to NCL mice.

Ensemble ID	Gene	Description	TgGRK5/ NLC fold	p-value
ENSMUSG00000028307	Aldob	aldolase B, fructose-bisphosphate	-5.382	3.68E-07
ENSMUSG00000037145	2210407C18Rik	RIKEN cDNA 2210407C18 gene	-4.209	1.85E-11
ENSMUSG00000003934	Efnb3	ephrin B3	-4.145	2.87E-09
ENSMUSG00000104713	Gbp6	guanylate binding protein 6	-3.746	1.24E-22
ENSMUSG00000036218	Pdzrn4	PDZ domain containing RING finger 4	-3.479	1.50E-05
ENSMUSG00000028036	Ptgfr	prostaglandin F receptor	-3.310	3.31E-05
ENSMUSG00000061762	Tac1	tachykinin 1	-3.173	7.79E-06
ENSMUSG00000032338	Hcn4	hyperpolarization-activated, cyclic nucleotide-gated K+ 4	-3.061	9.81E-07
ENSMUSG00000075270	Pde11a	phosphodiesterase 11A	-2.782	0.000808148
ENSMUSG00000098986	Gm28006	predicted gene, 28006	-2.711	0.003545646
ENSMUSG00000040276	Pacsin1	protein kinase C and casein kinase substrate in neurons 1	-2.695	6.14E-07
ENSMUSG00000055632	Hmcn2	hemicentin 2	-2.662	9.25E-09
ENSMUSG00000020218	Wif1	Wnt inhibitory factor 1	-2.659	3.52E-06
ENSMUSG00000030433	Sbk2	SH3-binding domain kinase family, member 2	-2.623	1.70E-05
ENSMUSG00000108942	Gm44660	predicted gene 44660	-2.577	0.015076817
ENSMUSG00000111765	Gm10635	predicted gene 10635	-2.502	0.000794162
ENSMUSG00000021730	Hcn1	hyperpolarization-activated, cyclic nucleotide-gated K+ 1	-2.447	0.000952307
ENSMUSG00000078815	Cacng6	calcium channel, voltage-dependent, gamma subunit 6	-2.437	0.000391715
ENSMUSG00000085162	Gm12295	predicted gene 12295	-2.325	3.46E-05
ENSMUSG00000058441	Panx2	pannexin 2	-2.317	0.001146055
ENSMUSG00000021250	Fos	FBJ osteosarcoma oncogene	-2.254	8.62E-05
ENSMUSG00000021453	Gadd45g	growth arrest and DNA-damage-inducible 45 gamma	-2.224	2.86E-05
ENSMUSG00000039488	Cntn5	contactin 5	-2.221	0.000743477
ENSMUSG00000083327	Vcp-rs	valosin containing protein, related sequence	-2.204	0.000374601
ENSMUSG00000050440	Hamp	hepcidin antimicrobial peptide	-2.197	0.003301044
ENSMUSG00000107653	Gm31520	predicted gene, 31520	-2.128	0.002111231
ENSMUSG00000036242	3632451O06Rik	RIKEN cDNA 3632451O06 gene	-2.122	0.00030777

ENSMUSG00000050541	Adra1b	adrenergic receptor, alpha 1b	-2.122	0.000180695
ENSMUSG00000027887	Sypl2	synaptophysin-like 2	-2.116	0.003482413
ENSMUSG00000025900	Rp1	retinitis pigmentosa 1 (human)	-2.069	0.002795274
ENSMUSG00000015647	Lama5	laminin, alpha 5	-2.057	0.000735852
ENSMUSG00000103837	Gm37614	predicted gene, 37614	-2.049	0.025291535
ENSMUSG00000023140	Reg2	regenerating islet-derived 2	-2.046	0.043569857
ENSMUSG00000028125	Abca4	ATP-binding cassette, sub-family A (ABC1), member 4	-2.034	0.000231836
ENSMUSG00000091956	C2cd4b	C2 calcium-dependent domain containing 4B	-2.028	0.006365193
ENSMUSG00000020926	Adam11	a disintegrin and metallopeptidase domain 11	-1.998	0.00017938
ENSMUSG00000025537	Phkg1	phosphorylase kinase gamma 1	-1.956	0.000148696
ENSMUSG00000085513	Gm16363	predicted gene 16363	-1.956	0.015378992
ENSMUSG00000020182	Ddc	dopa decarboxylase	-1.948	0.000675471
ENSMUSG00000034936	Arl4d	ADP-ribosylation factor-like 4D	-1.943	0.007884892
ENSMUSG00000040113	Mettl11b	methyltransferase like 11B	-1.941	0.008040517
ENSMUSG00000106106	CT010467.1	na	-1.934	0.005058308
ENSMUSG00000021265	Slc25a29	solute carrier family 25 (mitochondrial carrier, palmitoylcarnitine transporter), member 29	-1.933	0.008255198
ENSMUSG00000028825	Rhd	Rh blood group, D antigen	-1.933	0.007454189
ENSMUSG00000109353	Gm45183	predicted gene 45183	-1.929	0.009922826
ENSMUSG00000052188	Gm14964	predicted gene 14964	-1.918	0.006122198
ENSMUSG00000039137	Whrn	whirlin	-1.915	0.004356243
ENSMUSG00000010492	Uckl1os	uridine-cytidine kinase 1-like 1, opposite strand	-1.906	0.010805175
ENSMUSG00000060716	Plekhh1	pleckstrin homology domain containing, family H (with MyTH4 domain) member 1	-1.897	0.008287222
ENSMUSG00000113847	Gm48799	predicted gene, 48799	-1.881	0.003838923
ENSMUSG00000109335	Gm44845	predicted gene 44845	-1.850	0.023150199
ENSMUSG00000097839	Gm26909	predicted gene, 26909	-1.848	0.010229226
ENSMUSG00000021313	Ryr2	ryanodine receptor 2, cardiac	-1.842	0.001141459
ENSMUSG00000070495	Ctcfl	CCCTC-binding factor (zinc finger protein)-like	-1.842	0.021360889
ENSMUSG00000037341	Slc9a7	solute carrier family 9 (sodium/hydrogen exchanger), member 7	-1.840	0.004621513
ENSMUSG00000085666	Gm9855	predicted pseudogene 9855	-1.837	0.002238732
ENSMUSG00000035561	Aldh1b1	aldehyde dehydrogenase 1 family, member B1	-1.823	0.004968038
ENSMUSG00000105168	Gm30735	predicted gene, 30735	-1.819	0.001797521

ENSMUSG00000112800	Gm40604	predicted gene, 40604	-1.814	0.004733507
ENSMUSG00000052684	Jun	jun proto-oncogene	-1.805	0.013139323
ENSMUSG00000063765	Chadl	chondroadherin-like	-1.804	0.045738369
ENSMUSG00000096197	4930555G01Rik	RIKEN cDNA 4930555G01 gene	-1.804	0.002824125
ENSMUSG00000037826	Ppm1k	protein phosphatase 1K (PP2C domain containing)	-1.800	0.003950578
ENSMUSG00000072852	2310040G07Rik	RIKEN cDNA 2310040G07 gene	-1.800	0.014530802
ENSMUSG00000044349	Snhg11	small nucleolar RNA host gene 11	-1.796	0.014159372
ENSMUSG00000090389	Cdv3-ps	Cdv3 retrotransposed pseudogene	-1.767	0.002886308
ENSMUSG00000032523	Hhatl	hedgehog acyltransferase-like	-1.746	0.00136897
ENSMUSG00000024112	Cacna1h	calcium channel, voltage-dependent, T type, alpha 1H subunit	-1.746	0.000293496
ENSMUSG00000086740	Gm17029	predicted gene 17029	-1.716	0.013286617
ENSMUSG00000005089	Slc1a2	solute carrier family 1 (glial high affinity glutamate transporter), member 2	-1.715	0.007903268
ENSMUSG00000110489	Gm31659	predicted gene, 31659	-1.713	0.004727001
ENSMUSG00000091418	Gm3164	predicted gene 3164	-1.711	0.001324233
ENSMUSG00000022797	Tfrc	transferrin receptor	-1.706	0.004102279
ENSMUSG00000047281	Sfn	stratifin	-1.704	0.011258754
ENSMUSG00000098183	Gm27010	predicted gene, 27010	-1.701	0.020569164
ENSMUSG00000068699	Flnc	filamin C, gamma	-1.700	0.005984398
ENSMUSG00000103674	Gm37699	na	-1.699	0.016850652
ENSMUSG00000052837	Junb	jun B proto-oncogene	-1.698	0.001055205
ENSMUSG00000040287	Stac3	SH3 and cysteine rich domain 3	-1.698	0.011494796
ENSMUSG00000028909	Ptpru	protein tyrosine phosphatase, receptor type, U	-1.690	0.006769525
ENSMUSG00000034533	Scn10a	sodium channel, voltage-gated, type X, alpha	-1.681	0.037717587
ENSMUSG00000037490	Slc2a12	solute carrier family 2 (facilitated glucose transporter), member 12	-1.680	0.030087587
ENSMUSG00000006675	P4htm	prolyl 4-hydroxylase, transmembrane (endoplasmic reticulum)	-1.679	0.008011982
ENSMUSG00000047155	Cyp4x1	cytochrome P450, family 4, subfamily x, polypeptide 1	-1.678	0.016993314
ENSMUSG00000021208	Ifi27l2b	interferon, alpha-inducible protein 27 like 2B	-1.678	0.017354134
ENSMUSG00000092051	Gm17229	predicted gene 17229	-1.673	0.008121261
ENSMUSG00000105867	Gm42517	predicted gene 42517	-1.671	0.017168801
ENSMUSG00000047591	Mafa	v-maf musculoaponeurotic fibrosarcoma oncogene family, protein A (avian)	-1.671	0.009385168
ENSMUSG00000105444	Gm10727	predicted gene 10727	-1.670	0.012722575

ENSMUSG00000097805	Gm17473	predicted gene, 17473	-1.669	0.015959124
ENSMUSG00000085632	Gm12381	predicted gene 12381	-1.668	0.020142214
ENSMUSG00000051331	Cacna1c	calcium channel, voltage-dependent, L type, alpha 1C subunit	-1.664	0.005004419
ENSMUSG00000095294	Gm21119	predicted gene, 21119	-1.659	0.018859963
ENSMUSG00000021994	Wnt5a	wingless-type MMTV integration site family, member 5A	-1.659	0.001459794
ENSMUSG00000068606	Gm4841	predicted gene 4841	-1.657	0.024247685
ENSMUSG00000038216	Pnmt	phenylethanolamine-N-methyltransferase	-1.656	0.028529509
ENSMUSG00000022197	Pdzd2	PDZ domain containing 2	-1.653	0.000980717
ENSMUSG00000048915	Efna5	ephrin A5	-1.645	0.000128876
ENSMUSG00000041930	Fam222a	family with sequence similarity 222, member A	-1.645	0.010109906
ENSMUSG00000057716	Tmem178b	transmembrane protein 178B	-1.643	0.033892805
ENSMUSG00000113789	Gm47322	predicted gene, 47322	-1.642	0.021856244
ENSMUSG00000106995	Gm33167	predicted gene, 33167	-1.640	0.036256846
ENSMUSG00000105556	Gm43080	predicted gene 43080	-1.637	0.024065851
ENSMUSG00000030306	Tmtc1	transmembrane and tetratricopeptide repeat containing 1	-1.637	0.000767826
ENSMUSG00000028132	Tmem56	transmembrane protein 56	-1.632	0.011461343
ENSMUSG00000004891	Nes	nestin	-1.631	0.005572948
ENSMUSG00000049676	Catsperg1	cation channel sperm associated auxiliary subunit gamma 1	-1.631	0.019778622
ENSMUSG00000109045	Gm45084	predicted gene 45084	-1.629	0.02688937
ENSMUSG00000075383	Olfr351	olfactory receptor 351	-1.617	0.034894963
ENSMUSG00000108866	Gm35082	predicted gene, 35082	-1.617	0.01632363
ENSMUSG00000031880	Rrad	Ras-related associated with diabetes	-1.612	0.00063985
ENSMUSG00000006784	Ttc25	tetratricopeptide repeat domain 25	-1.610	0.029639415
ENSMUSG00000056753	C330011M18Rik	RIKEN cDNA C330011M18 gene	-1.610	0.020350657
ENSMUSG00000038984	Tspyl5	testis-specific protein, Y-encoded-like 5	-1.608	0.000946413
ENSMUSG00000105990	Gm43307	predicted gene 43307	-1.608	0.014076826
ENSMUSG00000022565	Plec	plectin	-1.605	0.003533736
ENSMUSG00000112855	Gm47842	predicted gene, 47842	-1.600	0.018615157
ENSMUSG00000048070	Pirt	phosphoinositide-interacting regulator of transient receptor potential channels	-1.597	0.010584447
ENSMUSG00000037949	Ano10	anoctamin 10	-1.596	0.017396541
ENSMUSG00000032511	Scn5a	sodium channel, voltage-gated, type V, alpha	-1.596	0.009063864

ENSMUSG00000110693	Gm45899	predicted gene 45899	-1.592	0.010615826
ENSMUSG00000104002	Gm38336	predicted gene, 38336	-1.587	0.028552651
ENSMUSG00000047205	Dusp18	dual specificity phosphatase 18	-1.581	0.014150903
ENSMUSG00000086127	Gm11934	predicted gene 11934	-1.579	0.031159058
ENSMUSG00000030235	Slco1c1	solute carrier organic anion transporter family, member 1c1	-1.576	0.030306438
ENSMUSG00000032845	Alpk2	alpha-kinase 2	-1.572	0.02233522
ENSMUSG00000041741	Pde3a	phosphodiesterase 3A, cGMP inhibited	-1.566	0.000658797
ENSMUSG00000110353	Gm33543	predicted gene, 33543	-1.566	0.033311882
ENSMUSG00000039414	Heatr5b	HEAT repeat containing 5B	-1.565	7.09E-05
ENSMUSG00000025243	Slc6a20b	solute carrier family 6 (neurotransmitter transporter), member 20B	-1.561	0.029457986
ENSMUSG00000026854	Usp20	ubiquitin specific peptidase 20	-1.553	0.010099521
ENSMUSG00000030276	Ttll3	tubulin tyrosine ligase-like family, member 3	-1.553	0.01372337
ENSMUSG00000038059	Smim3	small integral membrane protein 3	-1.552	0.000488102
ENSMUSG00000041313	Slc7a1	solute carrier family 7 (cationic amino acid transporter, y+ system), member 1	-1.546	2.19E-06
ENSMUSG00000015365	Mov10l1	Moloney leukemia virus 10-like 1	-1.545	0.015762371
ENSMUSG00000078684	5830417I10Rik	RIKEN cDNA 5830417I10 gene	-1.544	0.004715423
ENSMUSG00000073591	Pcdhb22	protocadherin beta 22	-1.541	0.01664132
ENSMUSG00000105340	Gm42878	predicted gene 42878	-1.541	0.033179826
ENSMUSG00000107495	Gm44215	predicted gene, 44215	-1.538	0.015469347
ENSMUSG00000054072	Iigp1	interferon inducible GTPase 1	-1.537	0.007607763
ENSMUSG00000025348	Itga7	integrin alpha 7	-1.536	0.007445816
ENSMUSG00000062257	Opcml	opioid binding protein/cell adhesion molecule-like	-1.536	0.039832487
ENSMUSG00000113328	Gm47260	predicted gene, 47260	-1.535	0.028482726
ENSMUSG00000053024	Cntn2	contactin 2	-1.530	0.040675537
ENSMUSG00000089719	Gm15758	predicted gene 15758	-1.530	0.020446364
ENSMUSG00000105368	Gm43759	predicted gene 43759	-1.529	0.049732084
ENSMUSG00000020866	Cacna1g	calcium channel, voltage-dependent, T type, alpha 1G subunit	-1.528	0.002645147
ENSMUSG00000043313	Pcdhb19	protocadherin beta 19	-1.520	0.019797071
ENSMUSG00000031712	Il15	interleukin 15	-1.519	0.029025069
ENSMUSG00000028399	Ptprd	protein tyrosine phosphatase, receptor type, D	-1.517	0.004355062
ENSMUSG00000072647	Adam1a	a disintegrin and metallopeptidase domain 1a	-1.515	0.04651203

ENSMUSG00000041757	Plekha6	pleckstrin homology domain containing, family A member 6	-1.514	0.008015163
ENSMUSG00000020961	Ston2	stonin 2	-1.514	0.028018043
ENSMUSG00000091071	1700030C10Rik	RIKEN cDNA 1700030C10 gene	-1.510	0.019467524
ENSMUSG00000108064	Gm44423	predicted gene, 44423	-1.510	0.049839219
ENSMUSG00000059325	Hopx	HOP homeobox	-1.506	0.037216403
ENSMUSG00000029597	Sds	serine dehydratase	-1.506	0.035229451
ENSMUSG00000102460	Gm38197	predicted gene, 38197	-1.502	0.043872699
ENSMUSG00000026565	Pou2f1	POU domain, class 2, transcription factor 1	-1.495	0.007250679
ENSMUSG00000035284	Vps13c	vacuolar protein sorting 13C	-1.493	0.015105003
ENSMUSG00000040663	Clcf1	cardiotrophin-like cytokine factor 1	-1.492	0.03079632
ENSMUSG00000072849	Serpina1e	serine (or cysteine) peptidase inhibitor, clade A, member 1E	-1.490	0.033761403
ENSMUSG00000045838	A430105I19Rik	RIKEN cDNA A430105I19 gene	-1.485	0.000393217
ENSMUSG00000051495	Irf2bp2	interferon regulatory factor 2 binding protein 2	-1.480	0.020619388
ENSMUSG00000103070	Gm37903	predicted gene, 37903	-1.479	0.027684509
ENSMUSG00000095762	Gm16378	predicted gene 16378	-1.477	0.026719796
ENSMUSG00000031965	Tbx20	T-box 20	-1.477	0.003726172
ENSMUSG00000075267	Pjvk	pejvakin	-1.474	0.023957623
ENSMUSG00000050592	Fam78a	family with sequence similarity 78, member A	-1.474	0.00649213
ENSMUSG00000074650	Gm10735	predicted gene 10735	-1.474	0.049739733
ENSMUSG00000035900	Gramd4	GRAM domain containing 4	-1.468	0.008439032
ENSMUSG00000032363	Adamts7	a disintegrin-like and metallopeptidase (reprolysin type) with thrombospondin type 1 motif, 7	-1.458	0.00622256
ENSMUSG00000101517	4732465J04Rik	RIKEN cDNA 4732465J04 gene	-1.457	0.042066716
ENSMUSG00000050919	Zfp366	zinc finger protein 366	-1.457	0.012653578
ENSMUSG00000021311	Mtr	5-methyltetrahydrofolate-homocysteine methyltransferase	-1.457	0.010606917
ENSMUSG00000026207	Speg	SPEG complex locus	-1.454	0.012794121
ENSMUSG00000111481	Gm47652	predicted gene, 47652	-1.451	0.010192303
ENSMUSG00000020393	Kremen1	kringle containing transmembrane protein 1	-1.445	0.011101115
ENSMUSG00000074063	Osgin1	oxidative stress induced growth inhibitor 1	-1.442	0.011071444
ENSMUSG00000036120	Rfxank	regulatory factor X-associated ankyrin-containing protein	-1.441	0.01141639
ENSMUSG00000105742	Gm42748	predicted gene 42748	-1.438	0.048639379
ENSMUSG00000079260	Tmppe	transmembrane protein with metallophosphoesterase domain	-1.438	0.030053282

ENSMUSG00000095040	1700001J03Rik	RIKEN cDNA 1700001J03 gene	-1.438	0.029638796
ENSMUSG00000025013	Tll2	tolloid-like 2	-1.437	0.0454742
ENSMUSG00000031161	Hdac6	histone deacetylase 6	-1.437	0.005406409
ENSMUSG00000003810	Mast2	microtubule associated serine/threonine kinase 2	-1.437	0.018249558
ENSMUSG00000042118	Bhmt2	betaine-homocysteine methyltransferase 2	-1.433	0.038200836
ENSMUSG00000110298	Gm8189	predicted gene 8189	-1.432	0.0471719
ENSMUSG00000108447	Gm44567	predicted gene 44567	-1.431	0.036115671
ENSMUSG00000002228	Ppm1j	protein phosphatase 1J	-1.428	0.014046442
ENSMUSG00000040987	Mill2	MHC I like leukocyte 2	-1.427	0.041573963
ENSMUSG00000000631	Myo18a	myosin XVIIIA	-1.424	0.013347464
ENSMUSG00000048108	Tmem72	transmembrane protein 72	-1.422	0.013812226
ENSMUSG00000090248	Gm14027	predicted gene 14027	-1.419	0.037502443
ENSMUSG00000097662	Gm2093	predicted gene 2093	-1.419	0.026387485
ENSMUSG00000059852	Kcng2	potassium voltage-gated channel, subfamily G, member 2	-1.418	0.027644472
ENSMUSG00000003378	Grik5	glutamate receptor, ionotropic, kainate 5 (gamma 2)	-1.417	0.016037442
ENSMUSG00000110711	Gm45760	predicted gene 45760	-1.416	0.025849643
ENSMUSG00000061393	Acvr2b	activin receptor IIB	-1.416	0.048105209
ENSMUSG00000051586	Mical3	microtubule associated monooxygenase, calponin and LIM domain containing 3	-1.415	0.047298633
ENSMUSG00000092278	Gm8752	predicted pseudogene 8752	-1.411	0.037731595
ENSMUSG00000014426	Map3k4	mitogen-activated protein kinase kinase kinase 4	-1.408	0.010438851
ENSMUSG00000101628	Gm28177	predicted gene 28177	-1.408	0.022173074
ENSMUSG00000043831	Lysmd4	LysM, putative peptidoglycan-binding, domain containing 4	-1.407	0.001390767
ENSMUSG00000051335	Gfod1	glucose-fructose oxidoreductase domain containing 1	-1.406	0.008396539
ENSMUSG00000051515	Fam181b	family with sequence similarity 181, member B	-1.404	0.028968315
ENSMUSG00000037692	Ahdc1	AT hook, DNA binding motif, containing 1	-1.403	0.001965663
ENSMUSG00000042744	Hectd4	HECT domain E3 ubiquitin protein ligase 4	-1.400	0.006496153
ENSMUSG00000066036	Ubr4	ubiquitin protein ligase E3 component n-recognin 4	-1.399	0.012208585
ENSMUSG00000038437	Mllt6	myeloid/lymphoid or mixed-lineage leukemia; translocated to, 6	-1.397	0.009898402
ENSMUSG00000053334	Ficd	FIC domain containing	-1.397	0.021611544
ENSMUSG00000054199	Gon4l	gon-4-like (C.elegans)	-1.395	0.001003904
ENSMUSG00000041731	Pgm5	phosphoglucomutase 5	-1.394	0.005583422

ENSMUSG00000016624	Phf21b	PHD finger protein 21B	-1.392	0.029077218
ENSMUSG00000024298	Zfp871	zinc finger protein 871	-1.389	0.031175001
ENSMUSG00000032737	Inppl1	inositol polyphosphate phosphatase-like 1	-1.389	0.00515151
ENSMUSG00000111761	9230112J17Rik	RIKEN cDNA 9230112J17 gene	-1.388	0.026586746
ENSMUSG00000038518	Jarid2	jumonji, AT rich interactive domain 2	-1.385	0.009171097
ENSMUSG00000035722	Abca7	ATP-binding cassette, sub-family A (ABC1), member 7	-1.383	0.046749197
ENSMUSG00000070733	Fryl	FRY like transcription coactivator	-1.382	0.006409305
ENSMUSG00000020029	Nudt4	nudix (nucleoside diphosphate linked moiety X)-type motif 4	-1.380	0.0085819
ENSMUSG00000104623	Gm6394	predicted gene 6394	-1.379	0.030815013
ENSMUSG00000002341	Ncan	neurocan	-1.377	0.040797661
ENSMUSG00000037443	Cep85	centrosomal protein 85	-1.377	0.024041817
ENSMUSG00000058056	Palld	palladin, cytoskeletal associated protein	-1.376	0.003240617
ENSMUSG00000054889	Dsp	desmoplakin	-1.375	0.044053557
ENSMUSG00000009739	Pou6f1	POU domain, class 6, transcription factor 1	-1.375	0.003336302
ENSMUSG00000024773	Atg2a	autophagy related 2A	-1.370	0.010018766
ENSMUSG00000008855	Hdac5	histone deacetylase 5	-1.369	0.005285148
ENSMUSG00000055639	Dach1	dachshund family transcription factor 1	-1.369	0.007068966
ENSMUSG00000036181	Hist1h1c	histone cluster 1, H1c	-1.367	0.011943805
ENSMUSG00000042524	Sun2	Sad1 and UNC84 domain containing 2	-1.364	0.042478731
ENSMUSG00000057133	Chd6	chromodomain helicase DNA binding protein 6	-1.361	0.016101044
ENSMUSG00000040648	Ppip5k2	diphosphoinositol pentakisphosphate kinase 2	-1.358	0.035589858
ENSMUSG00000021708	Rasgrf2	RAS protein-specific guanine nucleotide-releasing factor 2	-1.356	0.036301197
ENSMUSG00000030530	Furin	furin (paired basic amino acid cleaving enzyme)	-1.354	0.00276777
ENSMUSG00000032875	Arhgef17	Rho guanine nucleotide exchange factor (GEF) 17	-1.353	0.002720363
ENSMUSG00000026924	Sec16a	SEC16 homolog A, endoplasmic reticulum export factor	-1.352	0.010570827
ENSMUSG00000048027	Rgmb	repulsive guidance molecule family member B	-1.350	0.027356242
ENSMUSG00000049807	Arhgap23	Rho GTPase activating protein 23	-1.349	0.025694952
ENSMUSG00000025583	Rptor	regulatory associated protein of MTOR, complex 1	-1.347	0.01210866
ENSMUSG00000026596	Abl2	v-abl Abelson murine leukemia viral oncogene 2 (arg, Abelson-related gene)	-1.342	0.033921958
ENSMUSG00000034032	Rpap1	RNA polymerase II associated protein 1	-1.338	0.020331456
ENSMUSG00000028991	Mtor	mechanistic target of rapamycin kinase	-1.337	0.028485298

ENSMUSG00000039844	Rapgef1	Rap guanine nucleotide exchange factor (GEF) 1	-1.334	0.014274716
ENSMUSG00000036327	Qsox2	quiescin Q6 sulfhydryl oxidase 2	-1.333	0.015689371
ENSMUSG00000038342	Mlxip	MLX interacting protein	-1.332	0.001065304
ENSMUSG00000044447	Dock5	dedicator of cytokinesis 5	-1.330	0.033039296
ENSMUSG00000048154	Kmt2d	lysine (K)-specific methyltransferase 2D	-1.330	0.022895677
ENSMUSG00000030554	Synm	synemin, intermediate filament protein	-1.328	0.010152561
ENSMUSG00000108693	Gm45153	predicted gene 45153	-1.328	0.043450045
ENSMUSG00000002808	Epdr1	ependymin related protein 1 (zebrafish)	-1.328	0.001277155
ENSMUSG00000020455	Trim11	tripartite motif-containing 11	-1.326	0.002631623
ENSMUSG00000055707	Klhl26	kelch-like 26	-1.324	0.005912394
ENSMUSG00000039262	Prrc2b	proline-rich coiled-coil 2B	-1.324	0.006994434
ENSMUSG00000027086	Fastkd1	FAST kinase domains 1	-1.318	0.029686392
ENSMUSG00000032531	Amotl2	angiomotin-like 2	-1.318	0.026514368
ENSMUSG00000029714	Gigyf1	GRB10 interacting GYF protein 1	-1.317	0.011085893
ENSMUSG00000030533	Unc45a	unc-45 myosin chaperone A	-1.312	0.027893391
ENSMUSG00000020601	Trib2	tribbles pseudokinase 2	-1.311	0.013311148
ENSMUSG00000026932	Nacc2	nucleus accumbens associated 2, BEN and BTB (POZ) domain containing	-1.310	0.022630385
ENSMUSG00000038914	Dido1	death inducer-obliterator 1	-1.310	0.013523547
ENSMUSG00000053950	Adnp2	ADNP homeobox 2	-1.309	0.033820258
ENSMUSG00000101013	A630072M18Rik	RIKEN cDNA A630072M18 gene	-1.308	0.01587434
ENSMUSG00000032840	2410131K14Rik	RIKEN cDNA 2410131K14 gene	-1.308	0.012947378
ENSMUSG00000039087	Rreb1	ras responsive element binding protein 1	-1.307	0.040928626
ENSMUSG00000006205	Htra1	HtrA serine peptidase 1	-1.306	0.017820814
ENSMUSG00000002812	Flii	flightless I actin binding protein	-1.305	0.019769214
ENSMUSG00000075028	Prdm11	PR domain containing 11	-1.304	0.014781755
ENSMUSG00000040506	Ambra1	autophagy/beclin 1 regulator 1	-1.301	0.018766333
ENSMUSG00000042625	Safb2	scaffold attachment factor B2	-1.299	0.002331575
ENSMUSG00000020646	Mboat2	membrane bound O-acyltransferase domain containing 2	-1.296	0.019475913
ENSMUSG00000016128	Stard13	StAR-related lipid transfer (START) domain containing 13	-1.295	0.00114926
ENSMUSG00000025036	Sfxn2	sideroflexin 2	-1.289	0.044344943
ENSMUSG00000030956	Fam53b	family with sequence similarity 53, member B	-1.287	0.013730632
ENSMUSG00000028184	Adgrl2	adhesion G protein-coupled receptor L2	-1.286	0.003129886

ENSMUSG00000029338	Antxr2	anthrax toxin receptor 2	-1.286	0.019549946
ENSMUSG00000032842	Abcc10	ATP-binding cassette, sub-family C (CFTR/MRP), member 10	-1.282	0.040847326
ENSMUSG00000042650	Alkbh5	alkB homolog 5, RNA demethylase	-1.276	0.005609534
ENSMUSG00000067851	Arfgef1	ADP-ribosylation factor guanine nucleotide-exchange factor 1(brefeldin A-inhibited)	-1.273	0.028906556
ENSMUSG00000021820	Camk2g	calcium/calmodulin-dependent protein kinase II gamma	-1.270	0.004832668
ENSMUSG00000028849	Map7d1	MAP7 domain containing 1	-1.270	0.02508671
ENSMUSG00000025810	Nrp1	neuropilin 1	-1.268	0.009119515
ENSMUSG00000018340	Anxa6	annexin A6	-1.267	0.042218827
ENSMUSG00000027244	Atg13	autophagy related 13	-1.266	0.018441822
ENSMUSG00000022272	Myo10	myosin X	-1.261	0.009249254
ENSMUSG00000021140	Pcnx	pecanex homolog	-1.257	0.018401285
ENSMUSG00000040865	Ino80d	INO80 complex subunit D	-1.257	0.019931678
ENSMUSG00000037415	Ranbp10	RAN binding protein 10	-1.257	0.036486354
ENSMUSG00000025571	Tnrc6c	trinucleotide repeat containing 6C	-1.257	0.039788808
ENSMUSG00000034613	Ppm1h	protein phosphatase 1H (PP2C domain containing)	-1.257	0.030154464
ENSMUSG00000020658	Efr3b	EFR3 homolog B	-1.256	0.010171466
ENSMUSG00000031706	Rfx1	regulatory factor X, 1 (influences HLA class II expression)	-1.252	0.01373381
ENSMUSG00000060261	Gtf2i	general transcription factor II I	-1.250	0.042809377
ENSMUSG00000005886	Ncoa2	nuclear receptor coactivator 2	-1.250	0.046290319
ENSMUSG00000044730	9930104L06Rik	RIKEN cDNA 9930104L06 gene	-1.249	0.041354012
ENSMUSG00000037434	Slc30a1	solute carrier family 30 (zinc transporter), member 1	-1.248	0.007636238
ENSMUSG00000047417	Rexo1	REX1, RNA exonuclease 1	-1.247	0.036802484
ENSMUSG00000049800	Sertad2	SERTA domain containing 2	-1.242	0.031040953
ENSMUSG00000039671	Zmynd8	zinc finger, MYND-type containing 8	-1.242	0.036707162
ENSMUSG00000033004	Mycbp2	MYC binding protein 2	-1.239	0.01949292
ENSMUSG00000020740	Gga3	golgi associated, gamma adaptin ear containing, ARF binding protein 3	-1.239	0.033074519
ENSMUSG00000028430	Nol6	nucleolar protein family 6 (RNA-associated)	-1.239	0.033115147
ENSMUSG00000044864	Ankrd50	ankyrin repeat domain 50	-1.238	0.021717755
ENSMUSG00000040842	Szrd1	SUZ RNA binding domain containing 1	-1.236	0.003849545
ENSMUSG00000040390	Map3k10	mitogen-activated protein kinase kinase kinase 10	-1.234	0.036309799
ENSMUSG00000068747	Sort1	sortilin 1	-1.234	0.041074676

ENSMUSG00000032187	Smarca4	SWI/SNF related, matrix associated, actin dependent regulator of chromatin, subfamily a, member 4	-1.233	0.013518941
ENSMUSG00000034290	Nek9	NIMA (never in mitosis gene a)-related expressed kinase 9	-1.231	0.039867807
ENSMUSG00000033565	Rbfox2	RNA binding protein, fox-1 homolog (C. elegans) 2	-1.231	0.02892909
ENSMUSG00000015501	Hivep2	human immunodeficiency virus type I enhancer binding protein 2	-1.230	0.022128343
ENSMUSG00000027309	4930402H24Rik	RIKEN cDNA 4930402H24 gene	-1.229	0.011758499
ENSMUSG00000024969	Mark2	MAP/microtubule affinity regulating kinase 2	-1.228	0.027505558
ENSMUSG00000038855	Itpkb	inositol 1,4,5-trisphosphate 3-kinase B	-1.227	0.015936983
ENSMUSG00000063870	Chd4	chromodomain helicase DNA binding protein 4	-1.225	0.007570909
ENSMUSG00000053110	Yap1	yes-associated protein 1	-1.222	0.023768234
ENSMUSG00000002052	Supt6	suppressor of Ty 6	-1.221	0.045412925
ENSMUSG00000035139	Secisbp2	SECIS binding protein 2	-1.221	0.043327107
ENSMUSG00000035877	Zhx3	zinc fingers and homeoboxes 3	-1.218	0.019859254
ENSMUSG00000038025	Phf2	PHD finger protein 2	-1.218	0.023558038
ENSMUSG00000019132	BC005537	cDNA sequence BC005537	-1.213	0.004105095
ENSMUSG00000066406	Akap13	A kinase (PRKA) anchor protein 13	-1.212	0.023036568
ENSMUSG00000038023	Atp6v0a2	ATPase, H+ transporting, lysosomal V0 subunit A2	-1.209	0.023865607
ENSMUSG00000024400	Wdr33	WD repeat domain 33	-1.207	0.032029031
ENSMUSG00000043279	Trim56	tripartite motif-containing 56	-1.207	0.007355922
ENSMUSG00000008730	Hipk1	homeodomain interacting protein kinase 1	-1.202	0.041526701
ENSMUSG00000024096	Ralbp1	ralA binding protein 1	-1.201	0.029657635
ENSMUSG00000032194	Kank2	KN motif and ankyrin repeat domains 2	-1.200	0.008864475
ENSMUSG00000021738	Atxn7	ataxin 7	-1.199	0.033593909
ENSMUSG00000038002	Cramp1l	cramped chromatin regulator homolog 1	-1.197	0.048228337
ENSMUSG00000068036	Afdn	afadin, adherens junction formation factor	-1.193	0.008431711
ENSMUSG00000059273	Zc3h4	zinc finger CCCH-type containing 4	-1.193	0.016183524
ENSMUSG00000035569	Ankrd11	ankyrin repeat domain 11	-1.191	0.021762447
ENSMUSG00000035168	Tanc1	tetratricopeptide repeat, ankyrin repeat and coiled-coil containing 1	-1.191	0.033102345
ENSMUSG00000029439	Sfswap	splicing factor SWAP	-1.191	0.014915443
ENSMUSG00000038290	Smg6	Smg-6 homolog, nonsense mediated mRNA decay factor (C. elegans)	-1.190	0.038590815
ENSMUSG00000038773	Kdm3b	KDM3B lysine (K)-specific demethylase 3B	-1.185	0.027166442
ENSMUSG00000040054	Baz2a	bromodomain adjacent to zinc finger domain, 2A	-1.184	0.049606307

ENSMUSG00000024597	Slc12a2	solute carrier family 12, member 2	-1.178	0.03646555
ENSMUSG00000015165	Hnrnpl	heterogeneous nuclear ribonucleoprotein L	-1.149	0.031595294

Suppl. Table 4:

	BASELINE				4 WEEKS				8 WEEKS			
	**NLC-Sham**	**TgGRK2-Sham**	**NLC-MI**	**TgGRK2-MI**	**NLC-Sham**	**TgGRK2-Sham**	**NLC-MI**	**TgGRK2-MI**	**NLC-Sham**	**TgGRK2-Sham**	**NLC-MI**	**TgGRK2-MI**
**HR (bpm)**	479.9±23.35	511.1±17.34	467.2±9.8	493.80±7.97	555.1±17.9	569.1±18.2	515.7±17.86	516.6±13.10	523.0±27.4	533.2±25.0	502.4±19.2	521.2±15.7
**LVIDs (mm)**	2.49±0.12	2.48±0.21	2.57±0.11	2.44±0.11	2.16±0.17	1.97±0.16	3.66±0.23*,‡	4.61±0.28†,*,§	2.62±0.22	2.46±0.24	3.76±0.25‖,#	5.21±0.31*,†,**
**LVIDd (mm)**	3.59±0.14	3.55±0.19	3.69±0.09	3.51±0.12	3.35±0.18	3.35±0.14	4.48±0.20##,#,	5.31±0.23†,*,§	3.74±0.20	3.63±0.21	4.54±0.24††	5.75±0.28*,†,**
**Vol s (uL)**	22.49±2.78	23.31±4.57	24.94±2.44	22.03±2.40	16.38±3.25	13.01±2.43	60.73±10.21‖,††	104.30±13.65†,*, §	26.63±5.41	23.21±4.69	64.34±10.24	137.41±16.66*,†,**
**Vol d (uL)**	54.61±5.11	53.84±6.54	58.63±3.41	52.28±4.02	46.92±6.22	46.34±4.57	94.93±11.00‖,††	140.24±13.56†,*,§	60.99±8.11	57.12±7.50	98.14±12.37	169.34±17.60*,†,**
**EF (%)**	58.95±2.61	58.55±3.93	58.68±2.23	59.23±1.84	66.36±2.11	73.15±2.80	39.01±2.61*,†	29.02±3.59†,*,‡‡	58.15±3.60	61.88±4.59	36.82±2.86*,†	21.15±2.81*,†,§§
**FS (%)**	30.71±1.78	30.56±2.59	30.73±1.56	30.85±1.23	35.79±1.46	41.52±2.34	18.94±1.37*,†	13.93±1.85†,*	30.33±2.38	33.24±3.47	17.75±1.50*,‡	9.88±1.38*,†,§

Echocardiographic data from TgGRK2 and NLC mice subjected to MI or Sham operation. Left ventricular (LV) ejection fraction (EF), fractional shortening (FS), internal diameter in systole (LVIDs) and diastole (LVIDd), volume in systole (Vol s) and diastole (Vol d), and heart rate (HR) were evaluated in NLC and TgGRK2 mice at baseline and at 4 and 8 weeks post-myocardial infarction (MI) or sham operation. Data are shown as mean±S.E.M. *$p<0.0001$ vs TgGRK2-Sham; †$p<0.0001$ vs NLC-Sham; ‡$p<0.001$ vs NLC-Sham; §$p<0.01$ vs NLC-MI; ‖$p<0.05$ vs NLC-Sham; #$p<0.01$ vs TgGRK2-Sham; **$p<0.0001$ vs NLC-MI; ##$p<0.01$ vs NLC-Sham; ††$p<0.05$ vs TgGRK2-Sham; ‡‡$p<0.05$ vs NLC-MI; §§$p<0.001$ vs NLC-MI. Two-way ANOVA with Tukey's post hoc test was used between groups.

Suppl. Table 5:

	BASELINE				4 WEEKS				8 WEEKS			
	**WT-Sham**	**GRK5cKO-Sham**	**WT-MI**	**GRK5cKO-MI**	**WT-Sham**	**GRK5cKO-Sham**	**WT-MI**	**GRK5cKO-MI**	**WT-Sham**	**GRK5cKO-Sham**	**WT-MI**	**GRK5cKO-MI**
**HR (bpm)**	517.7±21.1	504.0±18.9	472.9±8.9	477.2±9.72	512.4±7.7	496.5±18.1	469.0±9.0	477.4±11.0	527.9±18.6	507.9±22.7	478.9±12.0	506.7±14.2
**LVIDs (mm)**	2.56±0.11	2.37±0.18	2.14±0.10	2.26±0.10	2.52±0.11	2.61±0.06	4.19±0.16*	3.92±0.11*	2.54±0.13	2.49±0.10	4.64±0.18*	4.24±0.16*
**LVIDd (mm)**	3.77±0.10	3.47±0.18	3.38±0.07	3.55±0.09	3.73±0.12	3.66±0.10	4.96±0.15*	4.81±0.11*	3.63±0.11	3.54±0.08	5.33±0.17*	5.16±0.15*
**Vol s (uL)**	24.39±2.67	20.77±3.20	16.40±1.78	18.35±1.95	23.40±2.39	24.98±1.43	81.52±7.69*	68.26±4.53§	24.12±3.27	22.44±2.08	103.96±9.42*	83.37±6.89*†
**Vol d (uL)**	61.30±3.78	51.54±5.75	47.65±2.40	53.60±3.35	60.10±4.72	56.97±3.49	118.95±8.46*	109.40±5.97*	56.21±4.24	52.77±2.92	140.85±10.00*	129.81±8.28*
**EF (%)**	60.86±2.57	61.50±2.83	67.46±2.47	66.97±1.97	61.44±2.18	55.67±2.29	33.25±1.80*	38.11±1.53*	58.07±3.06	58.09±2.00	28.13±2.01*	37.10±2.31*‡
**FS (%)**	32.25±1.79	32.49±2.00	37.46±1.92	36.74±1.49	32.55±1.61	28.54±1.53	15.92±0.92*	18.51±0.83§	30.30±2.12	30.03±1.33	13.35±1.02*	18.17±1.26*†

Echocardiographic data from GRK5cKO and WT mice subjected to MI or Sham operation. Left ventricular (LV) ejection fraction (EF), fractional shortening (FS), internal diameter in systole (LVIDs) and diastole (LVIDd), volume in systole (Vol s) and diastole (Vol d), and heart rate (HR) were evaluated in WT and GRK5cKO mice at baseline and at 4 and 8 weeks post-myocardial infarction (MI) or sham operation. Data are shown as mean±S.E.M. *$p<0.0001$ vs WT-Sham and GRK5cKO-Sham; †$p<0.05$ vs WT-MI; ‡$p<0.01$ vs WT-MI; §$p<0.0001$ vs WT-Sham and $p<0.001$ vs GRK5cKO-Sham. Two-way ANOVA with Tukey's post hoc test was used between groups.

Suppl. Table 6. RT-PCR Primers:

Gene name	Sequence
Mouse Nppa	Forward 5’-TGCCGGTAGAAGATGAGGTC-3’<br>Reverse 5’-TGCTTTTCAAGAGGGCAGAT-3’
Mouse Myh7	Forward 5’-GCCAACACCAACCTGTCCAAGTTC-3’<br>Reverse 5’-TGCAAAGGCTCCAGGTCTGAGGGC-3’
Mouse Nppb	Forward 5’-CTGAAGGTGCTGTCCCAGAT-3’<br>Reverse 5’-CCTTGGTCCTTCAAGAGCTG-3’
Mouse Ctgf	Forward 5’-GAGTGGAGCGCCTGTTCTAAG-3’<br>Reverse 5’-GCAGAAGGTATTGTCATTGGTAAC-3’
Mouse Col1a1	Forward 5’-ACGGCTGCACGAGTCACAC-3’<br>Reverse 5’-GGCAGGCGGGAGGTCTT-3’
Mouse Col3a1	Forward 5’-GTTCTAGAGGATGGCTGTACTAAACACA-3’<br>Reverse 5’-TTGCCTTGCGTGTTTGATATTC-3’
Mouse Mmp2	Forward 5’-CAGGGAATGAGTACTGGGTCTATT-3’<br>Reverse 5’-ACTCCAGTTAAAGGCAGCATCTAC-3’
Mouse Ifnγ	Forward 5’-TTGGCTTTGCAGCTCTTCCT-3’<br>Reverse 5’-TGACTGTGCCGTGGCAGTA-3’
Mouse Il1β	Forward 5’-GCTCATCTGGGATCCTCTCC-3’<br>Reverse 5’-CCTGCCTGAAGCTCTTGTTG-3’
Mouse Il6	Forward 5’-ACGGCCTTCCCTACTTCACA-3’<br>Reverse 5’-CATTTCCACGATTTCCCAGA-3’
Mouse Il18	Forward 5’-CACATGCGCCTTGTGATGAC-3’<br>Reverse 5’-TGCAGCCTCGGGTATTCTGT-3’
Mouse Il33	Forward 5’-ACCAGGTGCTACTACGCTACTATGAG-3’<br>Reverse 5’-CACACCGTCGCCTGATTG-3’
Mouse Cxcl1	Forward 5’-CACCTCAAGAACATCCAGAGCT-3’<br>Reverse 5’-CAAGCAGAACTGAACTACCATCG-3’
Mouse Cxcl2	Forward 5’-CCCAGACAGAAGTCATAGCCAC-3’<br>Reverse 5’-CTTCCGTTGAGGGACAGCAG-3’

Mouse Cxcl5	Forward 5'-CTCAGTCATAGCCGCAACCGAGC-3'<br>Reverse 5'-CGCTTCTTTCCACTGCGAGTGC-3'
Mouse Ccl2	Forward 5'-GCATCCACGTGTTGGCTCA-3'<br>Reverse 5'-CTCCAGCCTACTCATTGGGATCA-3'
Mouse Ccl3	Forward 5'-GCGCCATATGGAGCTGACAC -3'<br>Reverse 5'- TCAGGCATTCAGTTCCAGGT-3'
Mouse Ccl5	Forward 5'-CAGCAGCAAGTGCTCCAATCTT-3'<br>Reverse 5'-TTCTTGAACCCACTTCTTCTCTGG-3'
Mouse Ccl11	Forward 5'-TGCTCACGGTCACTTCCTTC-3'<br>Reverse 5'-CTTGAAGACTATGGCTTTCAGGGTG-3'
Mouse Tnfα	Forward 5'-GCCTCTTCTCATTCCTGCTTG-3'<br>Reverse 5'-CTGATGAGAGGGAGGCCATT-3'
Mouse Icam1	Forward 5'-CAATTTCTCATGCCGCACAG-3'<br>Reverse 5'-AGCTGGAAGATCGAAAGTCCG-3'
Mouse Vcam1	Forward 5'-TGAACCCAAACAGAGGCAGAGT-3'<br>Reverse 5'-GGTATCCCATCACTTGAGCAGG-3'
Mouse Rcan1	Forward 5'-TCCAGCTTGGGCTTGACTGAG-3'<br>Reverse 5'-ACTGGAAGGTGGTGTCCTTGT-3'
Mouse 18s	Forward 5'-TCAAGAACGAAAGTCGGAGG-3'<br>Reverse 5'-GGACATCTAAGGGCATCAC-3'

SUPPLEMENTARY METHODS

Experimental Animals

All animal procedures were approved by the Institutional Animal Care and Use Committee of Temple University and were performed in accordance with the NIH Guide for the Care and Use of Laboratory Animals. Transgenic mice with cardiomyocyte-specific GRK5 overexpression (TgGRK5 mice), transgenic mice with cardiomyocyte-specific GRK2 overexpression (TgGRK2 mice) and GRK5 cardiomyocyte-specific KO mice (GRK5cKO mice) have been used for our study and have all been previously described[1-3]. Of note, an initial cross between GRK5flox/flox and αMHC-Cre mice led to a cardiac-specific GRK5 deletion. This conditional deletion was maintained by continued breeding of the GRK5flox/flox with GRK5flox/flox/αMHC-Cre+/-[1]. TgGRK5, TgGRK2 and GRK5cKO mice were all backcrossed for > 12 generations. For all in vivo experiments, appropriate C57/Bl6 non-transgenic littermate control (NLC) mice or wild type (WT, specifically GRK5floxed) mice were used as controls. Male and female mice between 8-12 weeks of age were utilized in the studies presented herein. Mice were bred in a conventional animal facility under standard conditions with a 12h:12h light-dark cycle, and access to food (standard rodent diet) and water ad libitum. There was a 5% mortality rate during myocardial infarction (MI) surgery regardless of the genotype. Mice that died within 24h after MI surgery were not included neither in echocardiographic data nor in the analysis of survival rate. Statistical powering was performed using the nQuery Advisor 3.0 software (Statistical Solutions) for estimation of sample size. For all experiments, the calculations use $\alpha = 0.05$ and $\beta = 0.2$ (power, 0.80). On the basis of these calculations, our target was a minimum of 5 animals (powered for indices of myocardial function) per group to attain statistical significance, with a preference for 10 or more. Mice were euthanized with the following methods: carbon dioxide

100% (route: inhalation), followed by cervical dislocation; exsanguination under isoflurane 3% anesthesia (route: inhalation); isoflurane 3% (route: inhalation) followed by heart excision.

Coronary Artery Occlusion Surgery

MI was induced as previously described[4]. Mice were anesthetized with 3% isoflurane inhalation. A small skin incision was made, and the pectoral muscles were retracted to expose the fourth intercostal space. A small hole was made, and the heart was popped out. Left main descending coronary artery (LCA) was sutured ≈3 mm from its origin to assure a permanent occlusion, and the heart was placed back into the intrathoracic space, followed by closure of muscle and skin. The sham group underwent the same surgical procedure except that the LCA was not occluded. Animals were randomly assigned to MI or sham operation and, received a single dose (0.1 mg/kg) of buprenorphine immediately after surgery.

Echocardiography

To assess cardiac structure and function, transthoracic echocardiography was performed using a VisualSonics Vevo 2100 system (VisualSonics, Toronto, Ontario, Canada) with a MS400 (30-MHz centerline frequency) probe, as previously described[5, 6]. In brief, mice were anesthetized with isoflurane inhalation (induction 3% and maintenance 1.5–2%) and hair was removed from the chest (from the neckline to mid-chest level). Then, mice were placed in a supine position on a heated table (core temperature was maintained at 37 °C) with embedded ECG leads. B-and M-mode images were acquired from a parasternal short-axis view to evaluate left ventricular (LV) ejection fraction, fractional shortening, internal diameter in diastole and systole, volume in systole and diastole. Echocardiographic speckle-tracking based strain analyses were performed

on parasternal long axis B-mode loops as previously described[5]. Image depth, width and gain settings were optimized to improve image quality. B-mode loops with a frame rate ≥ 200 frame/sec were utilized. All images were digitally stored in cine loops consisting of 300 frames. All images were acquired and then analyzed using the Vevo Strain software (Vevo LAB 1.7.1) under their coded numbers in a blinded fashion, then the code was broken and statistical analysis were performed. Regional (6 LV segments: basal, mid, and apical anterior; basal, mid, and apical posterior) and global (average of the six LV segments) LV endocardial longitudinal and radial strain as well as strain rate were evaluated either in the radial or longitudinal axis. Echocardiographic images of poor quality were excluded from analyses.

Hemodynamic analysis

Hemodynamic analysis in vivo was performed as previously described[6, 7]. Mice were anesthetized with Tribromoethanol (Avertin, intraperitoneal dose of 2.5%, 20 µl/g body weight; used only for terminal hemodynamics). Avertin was chosen for anesthesia over other compounds because it has been shown to have less side effects on cardiovascular and hemodynamic stability and, therefore interferes less with the analysis of heart function and inotropic reserve during in vivo hemodynamic analysis[8, 9]. A recent review of the literature indicates that Avertin exerts less depressant effects on LV function and heart rate (HR) compared to isoflurane[9]. Moreover, compared with Avertin, a combination of xylazine and ketamine was demonstrated to produce profound bradycardia with effects on loading conditions and ventricular function[8]. At LV catheterization, systolic and diastolic function was particularly impaired in mice that showed HR <300 beats/min after xylazine and ketamine anesthesia[8]. The use of Avertin for the hemodynamic analysis was approved by the Institutional Animal Care and Use Committee of

Temple University. A 1.4-French micromanometer-tipped catheter (Millar Instruments) was inserted into the right carotid artery and then advanced into the LV. A polyethylene-50 catheter was placed in the left external jugular vein for incremental infusion of isoproterenol. LV maximum and minimum dP/dt as well as HR were recorded at baseline and after incremental doses of isoproterenol in closed-chest mode throughout the experiment with PowerLab System (Millar Instruments). Following the conductance catheter recordings, mice were injected with 200 units of heparin, IP. After a five-minute period to allow the heparin to circulate, the mouse was euthanized by carbon dioxide asphyxiation. After absence of respiratory movements and lack of response to painful stimulus were observed, death was confirmed by cervical dislocation.

RNA extraction and Real-time PCR

RNA extraction and Real-time PCR were performed as previously described[6, 10]. Total RNA was isolated from LV specimens with TRizol (Life Technologies, USA) accordingly to the company's instructions. Real-time PCR was performed on a CFX96 Real-Time PCR detection system (BioRad Lab. USA) or StepOnePlus Real-Time PCR System (Applied Biosystems) using appropriate SYBR green mix and specific primers (Suppl. Table 6). Real-time PCR was also performed using TaqMan Gene Expression Assays (ThermoFisher Scientific) and the StepOnePlus Real-Time PCR System (Applied Biosystems) for the following genes: Bex1 (Mm07295933_g1), Cxcl11 (Mm00444662_m1), Cxcl13 (Mm04214185_s1), IL-15 (Mm00434210_m1) and 18S (Mm03928990_g1).

Infarct size measurements and immunohistochemistry of cardiac sections

Twenty-four hours after myocardial infarction hearts were stained with triphenyltetrazolium chloride (TTC) to determine infarct size, as previously described[11].

Mouse hearts were fixed at least for 48h with 4% Paraformaldehyde and embedded in paraffin. Cardiac tissues were cut into 5.5 µm-thick sections. Following deparaffinization and rehydration mouse samples were mounted on glass slides and stained:

1) With Picrosirius Red Stain (#24901, Polyscience) to quantify cardiac fibrosis, as previously described[12]. Images were acquired with Nikon Eclipse Ni microscope using 10x objective. Quantitative image analysis was performed with NIH ImageJ.
2) With Trichrome Stain (Masson) (#HT15-1KT, Sigma-Aldrich) to measure infarct length, as previously described[13]. Images were acquired with SMZ1000 stereomicroscope. Quantitative image analysis was performed with NIH ImageJ.
3) For the Terminal deoxynucleotidyl transferase-mediated dUTP nick end-labelling (TUNEL) staining in red (Roche, 12156792910); the assay was performed according to manufacturer's instruction. Nuclei were counterstained with 4',6-diamidino-2-phenylindole dihydrochloride (DAPI; Sigma-Aldrich) while cardiomyocytes were counterstained with Cardiac Troponin T (MA5-12960, ThermoFisher, 1:100) followed by goat anti-mouse Alexa Fluor-488 1:100 (ThermoFisher)[14]. Multiple sections from the hearts were examined. Images were acquired with Nikon Eclipse Ti microscope or Nikon Eclipse Ni microscope using 20x objective. Quantitative image analysis was performed with NIS Elements software by scoring multiple imaging fields.
4) To examine vessels/cardiomyocyte ratio. Antigens were retrieved with 10mM citrate buffer pH=6 plus 0.05% of Tween. Hearts were incubated with antibodies against CD31 (R&D

systems AF3628, dilution 1:30), followed by donkey anti-goat Alexa Fluor-555 (1:100, Life technologies). Nuclei were counterstained with DAPI (Sigma-Aldrich) while cardiomyocytes were counterstained with Cardiac Troponin T (MA5-12960, ThermoFisher, 1:100) followed by goat anti-mouse Alexa Fluor-488 (1:100, ThermoFisher). Multiple sections from the hearts were examined. Images were acquired with Nikon Eclipse Ni microscope using 20x objective. Quantitative image analysis was performed with NIH ImageJ by scoring multiple imaging fields.

5) To examine the infiltration of various immune cell types[15]. Antigens were retrieved with a citrate-based antigen-unmasking solution (Vector Laboratories). Hearts were then blocked (10% FBS/PBS). A 0.3% H_2O_2 solution was also used to block endogenous peroxide activity (for DAB staining). Hearts were incubated with antibodies against CD45 (AF114; R&D Systems, dilution 1:100), CD3 (ab5690; Abcam, dilution 1:100), CD68 (ab31630; Abcam, dilution 1:100), mast cell tryptase (ab2378, Abcam, dilution 1:100), myeloperoxidase (sc-16129; Santa Cruz, dilution 1:100). Washed slides were incubated with the appropriate secondary antibodies, anti-mouse horseradish peroxidase (GE Healthcare, dilution 1:1000), anti-goat horseradish peroxidase (Santa Cruz, 1:1000), anti-goat Alexa Fluor 647 (Invitrogen, 1:1000), and anti-rabbit Alexa Fluor 647 (Invitrogen, 1:1000), followed by staining with DAPI for immunofluorescence. Immunofluorescent stained hearts were mounted with the Prolong Gold Antifade Reagent (Invitrogen). Immunohistochemically stained hearts were developed with a DAB Substrate Kit (Vector Laboratories) and mounted with Permount Mounting Media (Thermo Scientific). Staining was visualized on a Nikon Eclipse microscope at 20x magnification, and NIS Elements software was used for recording images and image analysis. Images were quantified as the number of positive cells per area.

Cardiomyocyte isolation and cell fractionation

Cardiomyocyte isolation was performed as previously described[10]. Adult ventricular cardiac myocytes were isolated from LV-free wall and septum of mice. Mice were injected with 200 units of heparin, intraperitoneally. After a 5 minute period to allow the heparin to circulate, the mouse was euthanized by being placed in a CO_2 chamber. After absence of respiratory movements and lack of response to painful stimulus was observed, death was confirmed by cervical dislocation. After removal from thoracic cavity, hearts were washed in cold perfusion buffer (NaCl 120.4 mmol l^{-1}, KCl 14.7 mmol l^{-1}, KH_2PO_4 0.6 mmol l^{-1}, Na_2HPO_4 0.6 mmol l^{-1}, $MgSO_4$-7H2O 1.2 mmol l^{-1}, Na-HEPES 10 mmol l^{-1}, $NaHCO_3$ 4.6 mmol l^{-1}, taurine 30 mmol l^{-1}, butanedione monoxime (BDM) 10 mmol l^{-1} and glucose 5.5 mmol l^{-1}, pH 7.0). Followed cannulation on a Langendorff system, they were perfused with perfusion buffer at 37 ^{0}C for 2–3 min and then digested with digestion solution (50 ml perfusion solution with collagenase 364 U ml^{-1}, 5 mg BD Difco-trypsin 250 and 20 $umol^{-1}$ $CaCl_2$) for 6–7 min. The atria were removed and the ventricles were gently minced with plastic pipettes in a 100 mm dish containing 2.5 ml of digestion solution. Once the ventricles are almost completely dissolved, the digestion is stopped adding 7 ml of stopping buffer. The resulting post-digestion solutions were filtered through a 100 um filter. The cardiomyocytes were separated from fibroblasts and smaller cells by 5-min gravity sedimentation and followed by centrifuging at 100g for 30 s. The supernatant was discarded and the pellet containing adult myocytes was resuspended in a stopping solution (perfusion buffer contains 250 mg BSA and 125 umol l^{-1} $CaCl_2$) and allowed to rest for 10 min. Different cell fractionations were obtained from isolated cardiomyocytes using BioVision FractionPREP™ Cell Fractionation Kit (Catalog #K270-50).

Western blot analysis

Western blotting was performed as previously described[10]. LV samples were lysed in a RIPA buffer with protease (cOmplete, Roche, Indianapolis, IN, USA) inhibitor cocktail. Protein content (from lysed LV samples or cell fractions from isolated cardiomyocyte) was quantified with the Bio-Rad BCA protein assay (Bio-Rad Laboratories, Richmond, California, USA). Protein samples were separated by 4–20% SDS–polyacrylamide gel electrophoresis (Thermo Fisher Scientific), and then transferred to nitrocellulose membrane (Bio-Rad Laboratories). After blocking, with a specific blocking buffer (Odyssey, LI-COR, Lincoln, Nebraska, USA), the membranes were incubated and probed with the first antibody at 4 ^{0}C overnight according to manufacturer's instructions. Then, the proteins were stained with a corresponding Alexa Fluor-680 (Thermo Fisher, dilution 1:5,000 or 1:10,000), IRDye 800CW (LI-COR, 1:5,000) or DyLigh-800 (Cell signaling, 1:5,000 or 1:10,000) secondary antibody, followed by visualization of the proteins with a LI-COR infrared imager (Odyssey), and quantitative densitometric analysis was performed applying Image Studio Software (LI-COR).

Protein levels of GRK5 (05-466; EMD Millipore, dilution 1:2,000; sc-518005; Santa Cruz; dilution 1:1,000), GRK2 (05-465; EMD Millipore, dilution 1:1,000), GAPDH (sc-32233 or sc-25778; Santa Cruz; dilution 1:2,000), Na^+/K^+ ATPase α -(sc-48345; Santa Cruz, dilution 1:2,000), Histone H1 (PA5-30055; Invitrogen, dilution 1:1,000) were assessed. Total Protein Stain (REVERT, LI-COR) or GAPDH have been used for western blot normalization.

FACS analysis

Immune cell isolation from the heart.and FACS analysis were performed as previously described[16, 17].

Immune cell isolation from heart. Non-myocyte cells were obtained from hearts after manual and enzymatic digestion. Briefly, whole hearts were isolated and flushed with PBS to remove the blood. Hearts were then manually digested into 1 mm^3 pieces, and incubated in a collagenase II (450 U/ml), MEM solution (Worthington Biochemical, Thermo Fisher Scientific). Digestion was performed for 1 hour in a shaking 37°C water bath. Cell suspensions were then collected and strained through an HBSS primed 70-µm cell strainer to filter debris and floating tissue pieces. Filtered cell suspensions were centrifuged at 1000 rpm for 5 minutes at 4°C and the supernatant was discarded. The cell pellets were resuspended in 1 ml of ACK lysing buffer (Thermo Fisher Scientific), incubated in a 37°C water bath for 5 minutes, and rinsed with 2 ml of HBSS. Cell suspensions were centrifuged once more at 1000 rpm for 5 minutes, 4°C before cell pellets were suspended in 2 ml of HBSS, counted with trypan blue, and used for flow cytometry analysis.

Fluorescence activated cell sorting (FACS) analysis. At least 1 million live cells were stained in FACS buffer (10% FBS in PBS) or binding buffer (Annexin V) (2.5 mM $CaCl_2$, 10 mM HEPES in FACS buffer). Each experiment included unstained cells from each heart and single-color compensations using UltraComp eBeads, ArC™ Amine Reactive Compensation Bead Kit (Thermo Fisher Scientific) where appropriate. All cells were stained with 2 independent antibody cocktails using CD45-BUV395 (1:100, BD Biosciences 564279), CD68-PE (1:100, BioLegend 137014), F4|80-AlexaFluor647 (1:100, Invitrogen MF48021), Ly6G-BV421 (1:100, BD Biosciences 562737), Ly6C-PE-Cy7 (1:500, BioLegend 128017), CD206-BV650 (1:100, Biolegend 141723), Annexin V-FITC (1:50, BioLegend 640905), CD3-BV421 (1:50, Biolegend 100227), CD117-APC-H7 (1:50, BD Biosciences 560185), CCR2-FITC (1:100, BioLegend 150608), CD19-BV650 (1:50, BD Biosciences 563235), Annexin V- AlexaFluor647 (1:50, Invitrogen, A23204), LIVE/DEAD Fixable Aqua Dead Cell Stain Kit (1:40, Invitrogen L34957).

Cells were stained for 1 hour in the dark, on ice, and were washed 3 times with PBS. Stained cell pellets were obtained after centrifugation for 5 minutes at 1000 rpm, 4°C. Gating strategy has been shown in Suppl. Figure 15. Data were acquired on an LSRII flow cytometer and analyzed using FlowJo software (Tree Star Inc.). In our flow cytometric quantitation, we have expressed specific immune cell populations as % of single cells or % of $CD45^+$ cells to understand their levels within the cardiac tissue or in the contest of the whole leukocyte population. Moreover, we have also normalized each sub-population over the appropriate immune cell population.

RNA-seq data analysis

Data was aligned using bowtie2 against mm10 genome and gene-level read counts were estimated using RSEM v1.2.12 software for ensemble transcriptome[18, 19]. DESeq2 was used to estimate significance between any two experimental groups and only genes passing $p<0.05$ thresholds**,** that had expression of at least 4 of 5 samples from one group higher or lower than expression of all samples from another group were considered (False Discovery Rate FDR<10% was used to filter results**)**[20]. Gene set enrichment analysis for a set of $p<0.05$ genes was done using QIAGEN's Ingenuity® Pathway Analysis software (IPA®, QIAGEN Redwood City, www.qiagen.com/ingenuity) using “Canonical Pathways” and “Network” options. Pathways that passed FDR<5% significance threshold and selected from top 25 networks were reported. RNA-seq data was deposited to GEO (https://www.ncbi.nlm.nih.gov/geo/) under accession number GSE132701.

Supplemental References

1. Gold JI, Gao E, Shang X, Premont RT, Koch WJ. Determining the absolute requirement of g protein-coupled receptor kinase 5 for pathological cardiac hypertrophy: Short communication. *Circ Res*. 2012;111:1048-1053
2. Koch WJ, Rockman HA, Samama P, Hamilton RA, Bond RA, Milano CA, Lefkowitz RJ. Cardiac function in mice overexpressing the beta-adrenergic receptor kinase or a beta ark inhibitor. *Science*. 1995;268:1350-1353
3. Rockman HA, Choi DJ, Rahman NU, Akhter SA, Lefkowitz RJ, Koch WJ. Receptor-specific in vivo desensitization by the g protein-coupled receptor kinase-5 in transgenic mice. *Proc Natl Acad Sci U S A*. 1996;93:9954-9959
4. Gao E, Lei YH, Shang X, Huang ZM, Zuo L, Boucher M, Fan Q, Chuprun JK, Ma XL, Koch WJ. A novel and efficient model of coronary artery ligation and myocardial infarction in the mouse. *Circ Res*. 2010;107:1445-1453
5. de Lucia C, Wallner M, Eaton DM, Zhao H, Houser SR, Koch WJ. Echocardiographic strain analysis for the early detection of left ventricular systolic/diastolic dysfunction and dyssynchrony in a mouse model of physiological aging. *J Gerontol A Biol Sci Med Sci*. 2019;74:455-461
6. Lieu M, Traynham CJ, de Lucia C, Pfleger J, Piedepalumbo M, Roy R, Petovic J, Landesberg G, Forrester SJ, Hoffman M, Grisanti LA, Yuan A, Gao E, Drosatos K, Eguchi S, Scalia R, Tilley DG, Koch WJ. Loss of dynamic regulation of g protein-coupled receptor kinase 2 by nitric oxide leads to cardiovascular dysfunction with aging. *Am J Physiol Heart Circ Physiol*. 2020;318:H1162-H1175

7. Sato PY, Chuprun JK, Grisanti LA, Woodall MC, Brown BR, Roy R, Traynham CJ, Ibetti J, Lucchese AM, Yuan A, Drosatos K, Tilley DG, Gao E, Koch WJ. Restricting mitochondrial grk2 post-ischemia confers cardioprotection by reducing myocyte death and maintaining glucose oxidation. *Sci Signal*. 2018;11

8. Hart CY, Burnett JC, Redfield MM. Effects of avertin versus xylazine-ketamine anesthesia on cardiac function in normal mice. *Am J Physiol Heart Circ Physiol*. 2001;281:H1938-1945

9. Pachon RE, Scharf BA, Vatner DE, Vatner SF. Best anesthetics for assessing left ventricular systolic function by echocardiography in mice. *Am J Physiol Heart Circ Physiol*. 2015;308:H1525-1529

10. Cannavo A, Liccardo D, Eguchi A, Elliott KJ, Traynham CJ, Ibetti J, Eguchi S, Leosco D, Ferrara N, Rengo G, Koch WJ. Myocardial pathology induced by aldosterone is dependent on non-canonical activities of g protein-coupled receptor kinases. *Nat Commun*. 2016;7:10877

11. Bathgate-Siryk A, Dabul S, Pandya K, Walklett K, Rengo G, Cannavo A, De Lucia C, Liccardo D, Gao E, Leosco D, Koch WJ, Lymperopoulos A. Negative impact of β-arrestin-1 on post-myocardial infarction heart failure via cardiac and adrenal-dependent neurohormonal mechanisms. *Hypertension*. 2014;63:404-412

12. Cannavo A, Rengo G, Liccardo D, Pun A, Gao E, George AJ, Gambino G, Rapacciuolo A, Leosco D, Ibanez B, Ferrara N, Paolocci N, Koch WJ. Β1-blockade prevents post-ischemic myocardial decompensation via β3ar-dependent protective sphingosine-1 phosphate signaling. *J Am Coll Cardiol*. 2017;70:182-192

13. Schumacher SM, Gao E, Zhu W, Chen X, Chuprun JK, Feldman AM, Tesmer JJ, Koch WJ. Paroxetine-mediated grk2 inhibition reverses cardiac dysfunction and remodeling after myocardial infarction. *Sci Transl Med*. 2015;7:277ra231

14. Cimini M, Garikipati VNS, de Lucia C, Cheng Z, Wang C, Truongcao MM, Lucchese AM, Roy R, Benedict C, Goukassian DA, Koch WJ, Kishore R. Podoplanin neutralization improves cardiac remodeling and function after acute myocardial infarction. *JCI Insight*. 2019;5

15. Grisanti LA, Gumpert AM, Traynham CJ, Gorsky JE, Repas AA, Gao E, Carter RL, Yu D, Calvert JW, García AP, Ibáñez B, Rabinowitz JE, Koch WJ, Tilley DG. Leukocyte-expressed β2-adrenergic receptors are essential for survival after acute myocardial injury. *Circulation*. 2016;134:153-167

16. Grisanti LA, de Lucia C, Thomas TP, Stark A, Strony JT, Myers VD, Beretta R, Yu D, Sardu C, Marfella R, Gao E, Houser SR, Koch WJ, Hamad EA, Tilley DG. Prior β-blocker treatment decreases leukocyte responsiveness to injury. *JCI Insight*. 2019;5

17. Ma Y, Mouton AJ, Lindsey ML. Cardiac macrophage biology in the steady-state heart, the aging heart, and following myocardial infarction. *Transl Res*. 2018;191:15-28

18. Langmead B, Salzberg SL. Fast gapped-read alignment with bowtie 2. *Nat Methods*. 2012;9:357-359

19. Li B, Dewey CN. Rsem: Accurate transcript quantification from rna-seq data with or without a reference genome. *BMC Bioinformatics*. 2011;12:323

20. Love MI, Huber W, Anders S. Moderated estimation of fold change and dispersion for rna-seq data with deseq2. *Genome Biol*. 2014;15:550

FULL WESTERN BLOTS

Full Western blot – Figure 1A

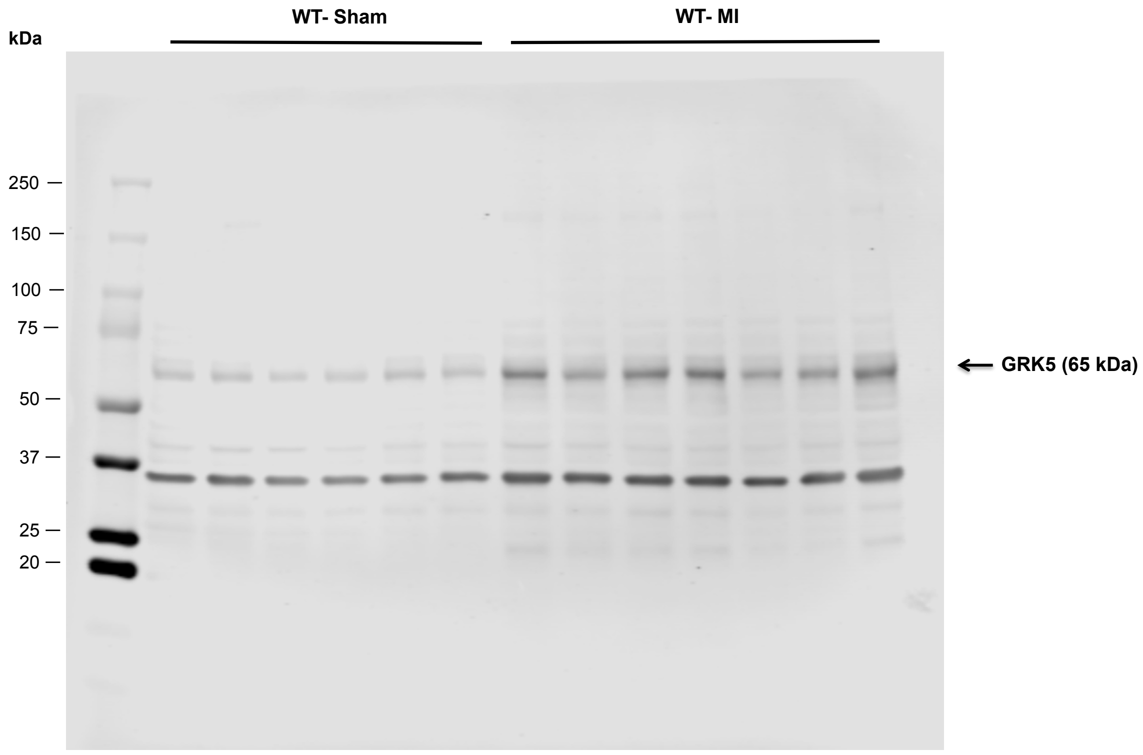

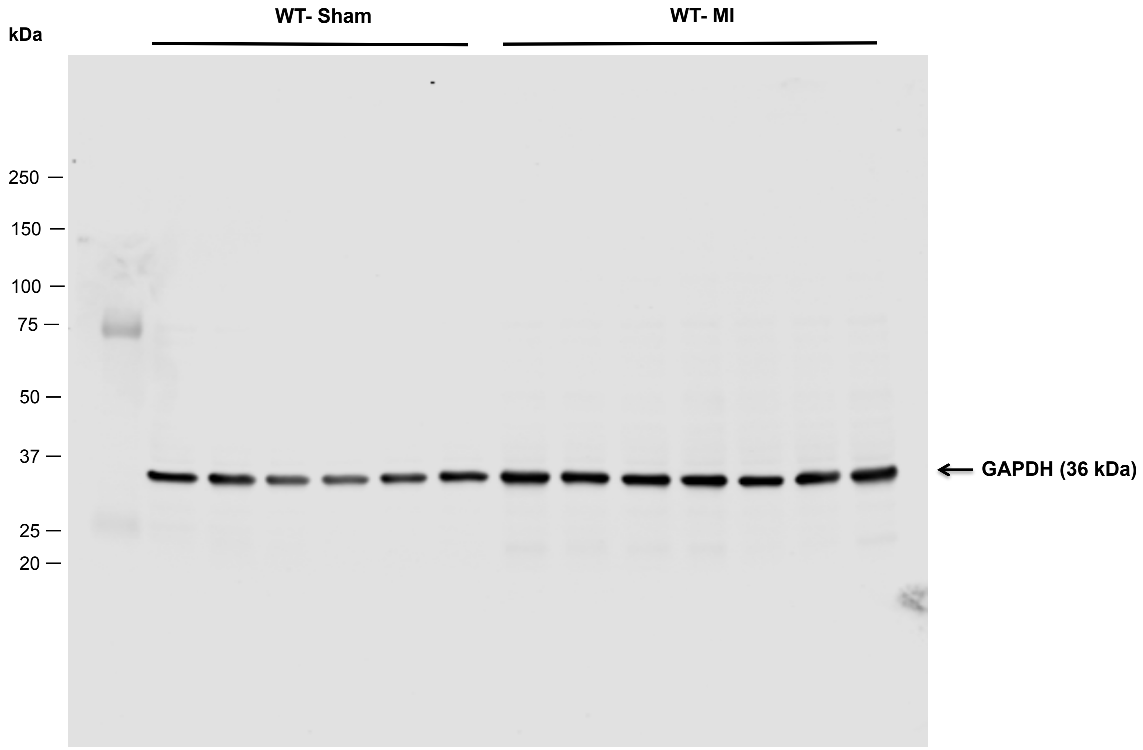

Full Western blot – Suppl. Figure 1A

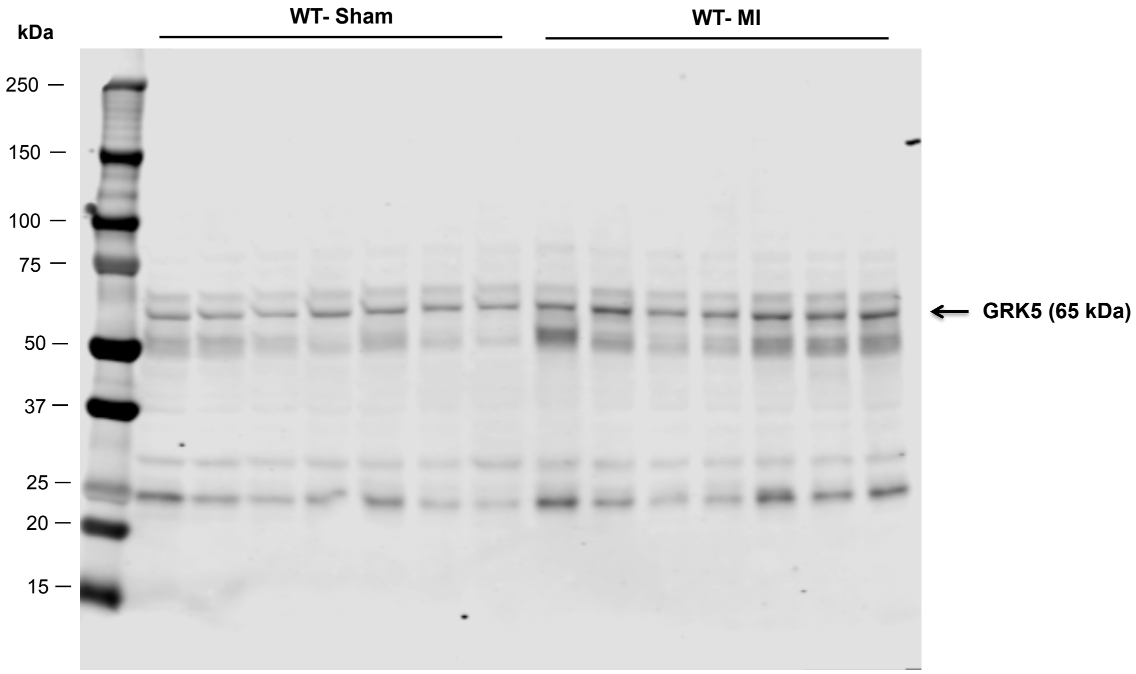

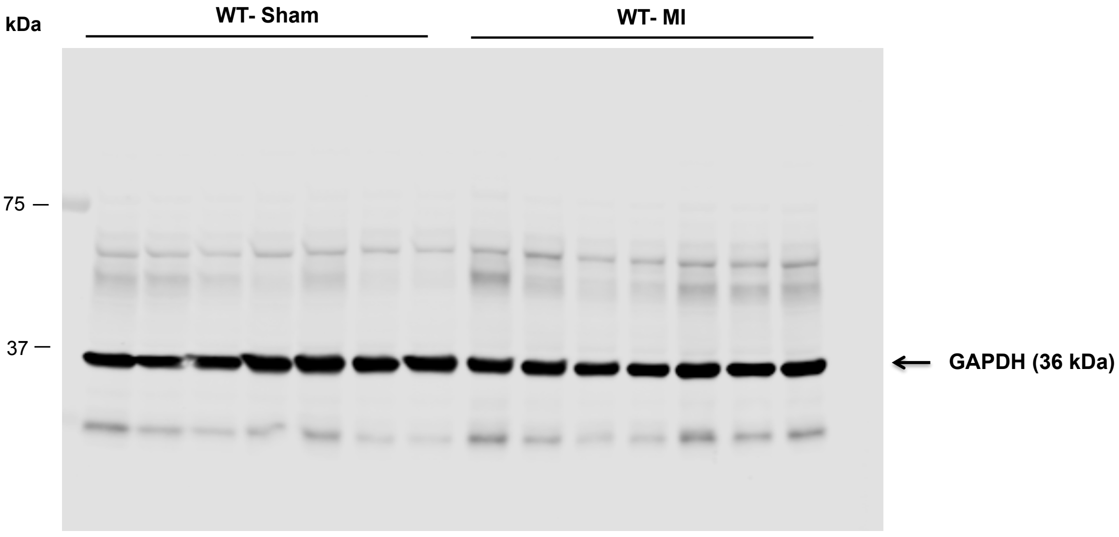

Full Western blot – Suppl. Figure 1C

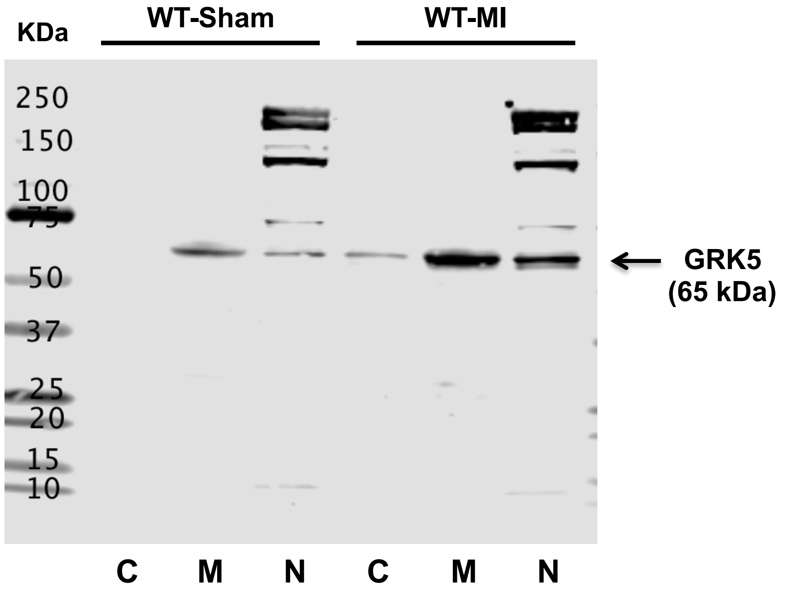

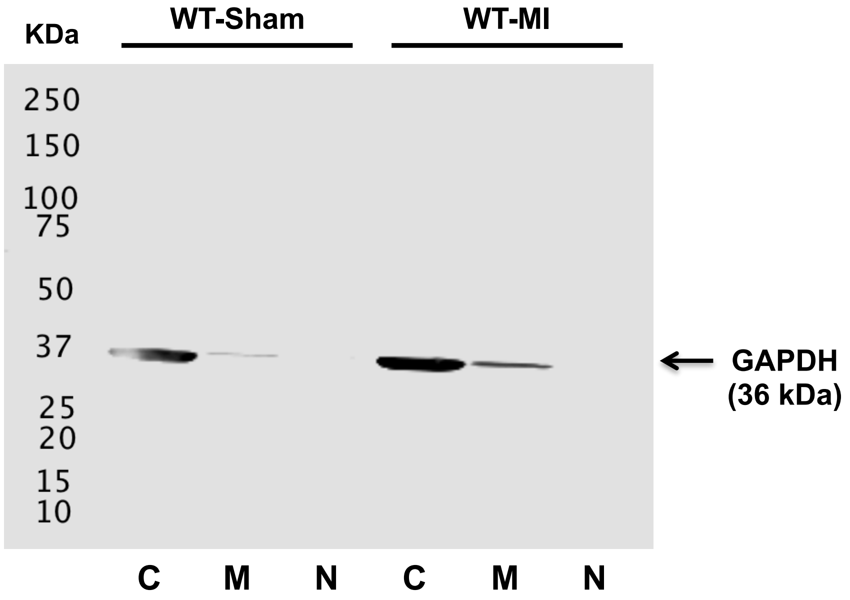

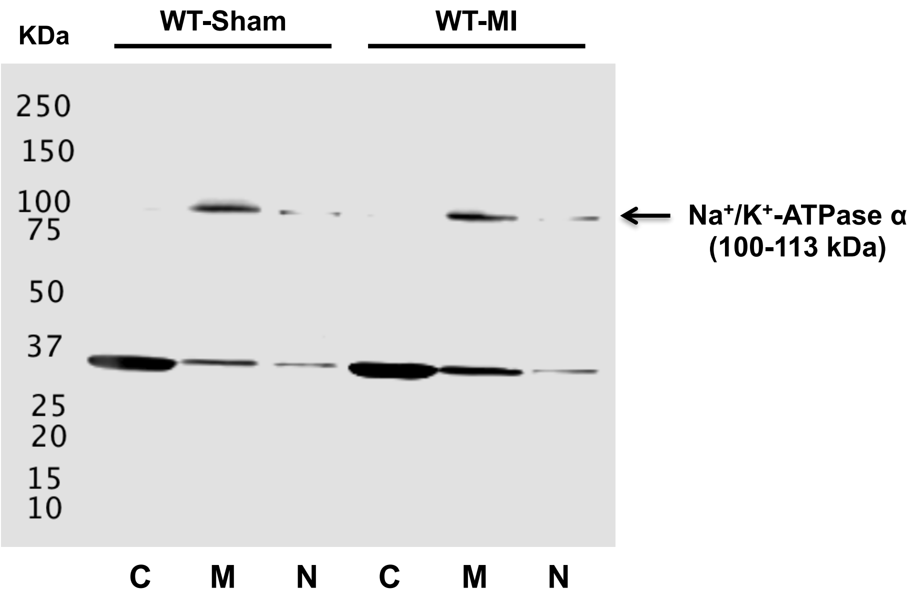
KDa
WT-Sham
WT-MI
250
150
100
75
50
37
25
20
15
10
Na+/K+-ATPase α
(100-113 kDa)
C
M
N
C
M
N

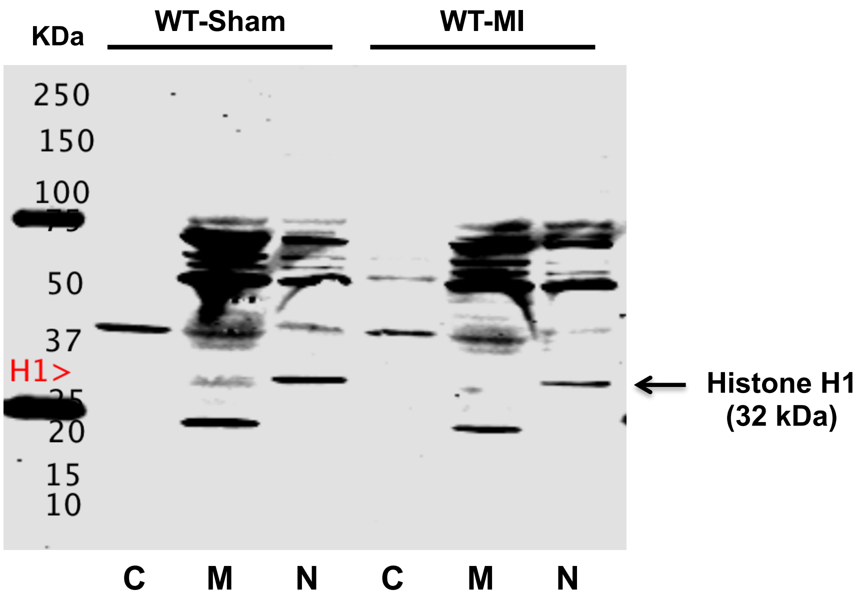
KDa
WT-Sham
WT-MI
250
150
100
50
37
H1>
20
15
10
Histone H1
(32 kDa)
C
M
N
C
M
N

Full Western blot – Suppl. Figure 7B

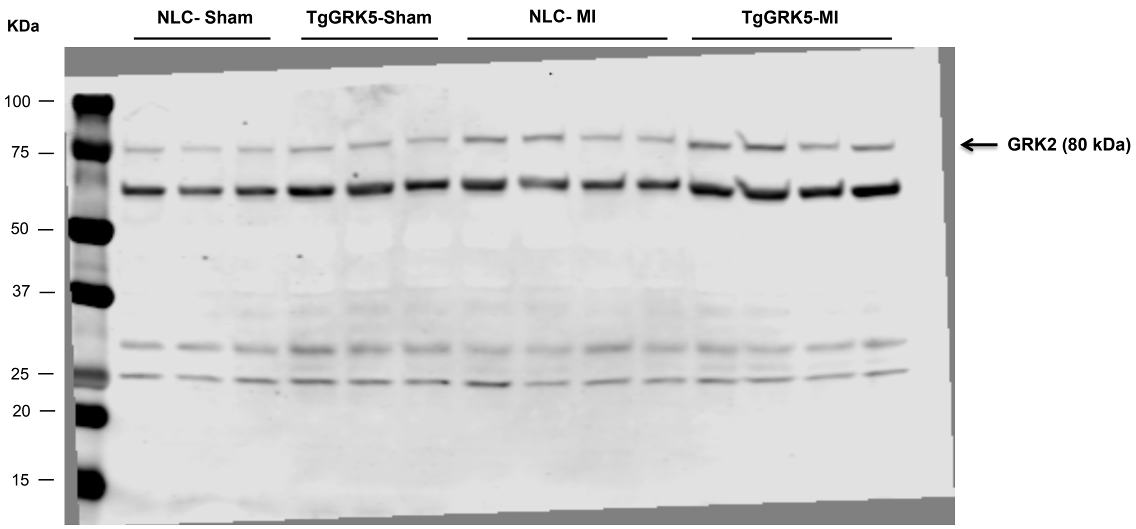

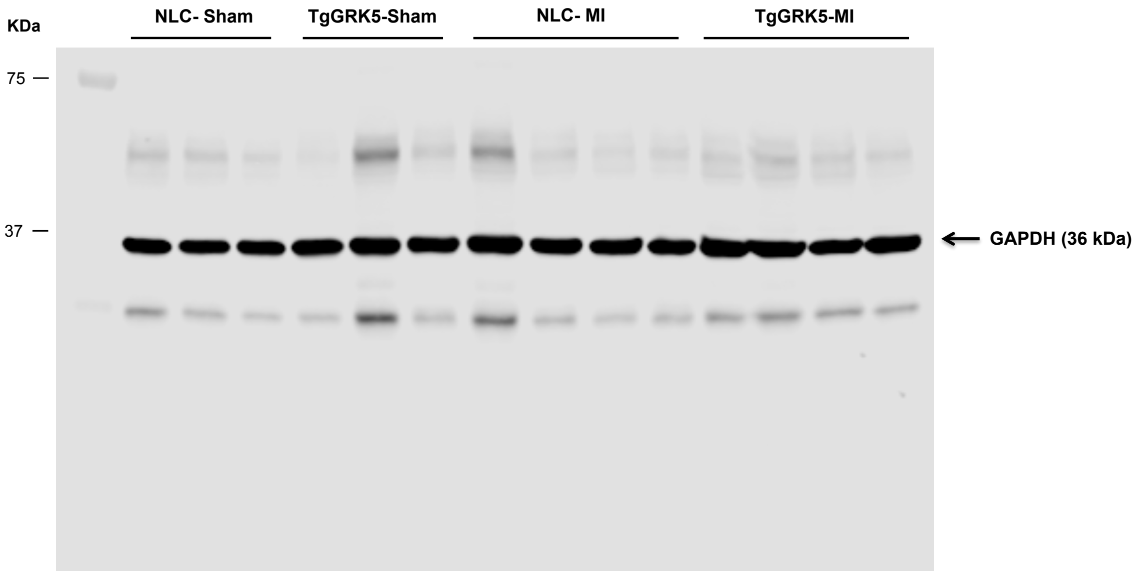

Supplement: cvab044_Supplementary_Data [file cvab044_supplementary_data.pdf]
